# Genetic diversity and association mapping in the Colombian Central Collection of *Solanum tuberosum* L. Andigenum group using SNPs markers

**DOI:** 10.1371/journal.pone.0173039

**Published:** 2017-03-03

**Authors:** Jhon Berdugo-Cely, Raúl Iván Valbuena, Erika Sánchez-Betancourt, Luz Stella Barrero, Roxana Yockteng

**Affiliations:** 1 Colombian Agricultural Research Corporation (CORPOICA)-Mosquera, Cundinamarca, Colombia; 2 Muséum National d’Histoire Naturelle, UMR-CNRS 7205, Paris, France; Agriculture and Agri-Food Canada, CANADA

## Abstract

The potato (*Solanum tuberosum* L.) is the fourth most important crop food in the world and Colombia has one of the most important collections of potato germplasm in the world (the Colombian Central Collection-CCC). Little is known about its potential as a source of genetic diversity for molecular breeding programs. In this study, we analyzed 809 Andigenum group accessions from the CCC using 5968 SNPs to determine: 1) the genetic diversity and population structure of the Andigenum germplasm and 2) the usefulness of this collection to map qualitative traits across the potato genome. The genetic structure analysis based on principal components, cluster analyses, and Bayesian inference revealed that the CCC can be subdivided into two main groups associated with their ploidy level: Phureja (diploid) and Andigena (tetraploid). The Andigena population was more genetically diverse but less genetically substructured than the Phureja population (three vs. five subpopulations, respectively). The association mapping analysis of qualitative morphological data using 4666 SNPs showed 23 markers significantly associated with nine morphological traits. The present study showed that the CCC is a highly diverse germplasm collection genetically and phenotypically, useful to implement association mapping in order to identify genes related to traits of interest and to assist future potato genetic breeding programs.

## Introduction

*Solanum tuberosum* L. is a herbaceous species that reproduces mainly vegetatively by tubers, distributed from the Southwestern United States to South-central Chile, with centers of diversity located in Central Mexico and in the high Andes from Peru to Northwestern Argentina [1]. Potato is the fourth most important crop food in the world after corn, rice and wheat [2]. It is consumed by people worldwide either as a non-grain staple or as a vegetable. It has high nutrient value providing carbohydrates, proteins, vitamins and minerals [3]. *Solanum tuberosum* contains two cultivar groups, the Chilotanum group comprising lowland tetraploid Chilean landraces [4] and the Andigenum group comprising upland Andean genotypes. Andigenum group varies in its ploidy level, going from diploids with 24 chromosomes to hexaploids with 72 [4]. Within the Andigenum group, the most important potatoes are commonly known as “Andigenas”, which are autotetraploid (2n = 4x = 48), highly heterozygous with tetrasomic inheritance, adapted to tuberization under short days and have tuber dormancy [5, 6]. In Andigenum, a group of diploids (2n = 2x = 24) known as “Phurejas” can also be distinguished. These potatoes have a short vegetative period, form small tubers and lack dormancy [5, 7]. They were cultivated from central Peru to Ecuador, Colombia, and Venezuela [8]. Another group in Andigenum, known as “Chauchas”, are triploid potatoes (2n = 3x = 36) generated by natural hybridization between the species *S*. *tuberosum* subsp. *andigena* and *S*. *stenotonum*, and they are cultivated in Peru, with lower frequency in Bolivia, Ecuador and Colombia [8].

The conservation of cultivated potato species and their wild relatives in germplasm banks provides long-term availability of crop genetic diversity. The characterization of these collections are essential to identify alleles/genes associated with traits of interest for plant breeding such as resistance to pathogens and insect pests, tolerance of abiotic stresses (e.g. salinity and frost) and tuber quality [9, 10]. In Colombia, part of the diversity of potato genetic resources (2069 accessions) are maintained in the Potato Germplasm Bank located at the Colombian Agricultural Research Corporation (CORPOICA). Within this germplasm bank, a subset of potatoes (826 accessions) known as Colombian Central Collection (CCC), is recognized as one of the most diverse potato germplasm in the world, after the CIP (International Potato Center) collection that has over 6000 accessions including cultivated species and potato wild relatives [11, 12, 13]. The Universidad Nacional de Colombia conserves also a Phureja potato collection (Colombian Core Collection-CCC). Hence, the CCC-CORPOICA is a potential source of novel alleles of agronomic value that could help to generate new potato cultivars with increased productivity. However, the appropriate use of genetic resources conserved in the CCC, depends on the understanding of their phenotypic and genetic diversity.

Genetic diversity could be analyzed from agronomic traits data, but the results obtained are not always robust because the environment often affects phenotypic traits [14]. In addition, the phenotypic variability would be the result of the interaction and segregation of few major genes widely distributed in a germplasm collection. Rare alleles cannot be generally detected or preserved [13]. Therefore, the combination of phenotypic and molecular data could provide a better estimation of the genetic diversity [15]. Molecular markers have been successfully used in the analysis of genetic diversity and population structure, linkage disequilibrium and localization of monogenic or polygenic traits [16]. The genetic diversity in potato has been studied through different molecular markers as random amplified polymorphic DNA (RAPD), amplified fragment length polymorphism (AFLP), inter simple sequence repeats (ISSR), and simple sequence repeat (SSR) [5, 7, 17]. So far, only one study using 42 SSRs, analyzed 97 diploid accessions (Phurejas) of the CCC-Universidad Nacional de Colombia has been reported [18]. However, the genetic diversity of Andigenum group of the CCC-CORPOICA has not been yet characterized with molecular markers.

Currently, two beadchips with SNP array technology for genotyping potato at high-density genome-wide level are available, the Infinium 8K potato SNP array [19] and the 20K SNP array [20]. The 8K SolCAP array contains a subset of 8303 SNPs selected from transcriptome data and Sanger EST (Expressed Sequence Tag) database with 69.011 high confidence SNPs identified among six North American cultivars [21]. The 8K array has been used to study the genetic diversity of American [9] and European potatoes [22], to infer phylogenetic relationship among species of *Solanum* section *Petota* [23] and to identify candidate genes through linkage mapping [19, 24, 25] and association mapping [26–28].

By combining molecular and morphological data from the potato germplasm of CCC is possible to map simple or complex traits and subsequently to identify candidate genes through Genome-Wide Association Studies (GWAS) or Association mapping (AM). Such studies provide an efficient way to map quantitative trait loci (QTL) in natural populations or germplasm collections because they can detect historical recombination events and provide high mapping resolution [29–31]. The number of molecular markers required for implementing GWAS and the resolution for QTL mapping, is determined by the rate of LD decay between loci through the genome [32]. Although the LD decay in potato populations has been previously calculated, all reports differ: 265 bp (base pairs) [22], 1 cM (centiMorgan) [33], 5 cM [34] and 10 cM [35]. The incongruence between studies is probably due to differences in number, type and origin of samples and the type and number of molecular markers used. It is then necessary to calculate the LD background in this study.

In the present study, a genetic analysis of the CCC of *S*. *tuberosum* Andigenum group was conducted based on SNPs markers in order to evaluate its population structure and genetic diversity. Also, the extent of the linkage disequilibrium between pairs of SNPs markers was estimated in order to determine the utility of this germplasm and the molecular markers used to implement association-mapping studies. Accordingly, association mapping in tetraploid potatoes was conducted using morphological traits related with stem, berry, tuber and flower variables.

## Materials and methods

### Plant material

**Fig 1:**
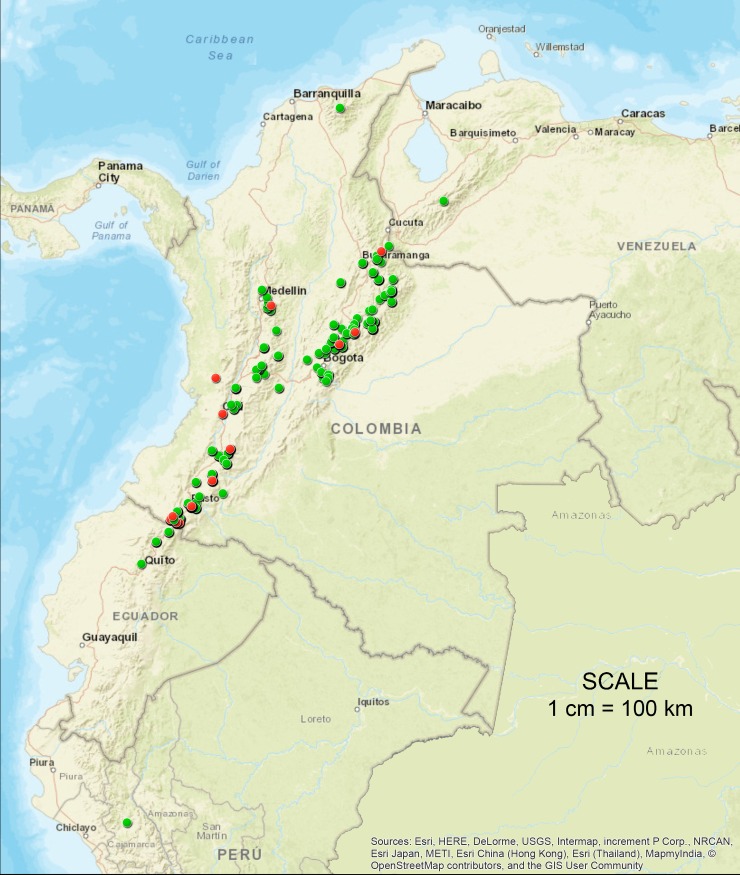
Map of geographical distribution of potato accessions from the Colombian Central Collection with passport data. Each accession is represented by a circle in which color indicates their classification in a particular population based on the results of software Structure (Red: Phureja, Green: Andigena).

A total of 809 accessions (one clone randomly selected from 16 clones grown per accession) of the CCC-CORPOICA of *S*. *tuberosum* group Andigenum conserved under field conditions in Zipaquira, Cundinamarca, Colombia (5° 03” 34.36” N, 74° 03” 29.61 W, 2.950 m altitude, average temperature 15°C and relative humidity of 75%) were characterized. Six hundred seventy-five accessions are classified from passport data as Andigena (83.5%), 85 as Phureja (10.5%) and 49 as Chaucha (6.0%). Six hundred and sixteen accessions were collected from different Colombia regions (76.1%), 75 accessions from other countries (9.3%) and 118 accessions do not have passport data (14.6%) (Fig 1, Table 1). The information of each accession is presented in the S1 Table.

**Table 1:** Summary of the 809 accessions of the Colombian Central Collection of *S*. *tuberosum* Andigenum group used in this study.

CCC	Country	Department	Number of accessions
**Andigena**	Colombia	Nariño	201
		Boyacá	110
		Cauca	63
		Cundinamarca	62
		Santander	21
		N. de Santander	19
		V. del Cauca	16
		Antioquia	12
		Quindio	10
		Caldas	8
		Tolima	8
		Magdalena	1
		Unknown	25
	Peru	-	39
	Bolivia	-	11
	Ecuador	-	11
	United States	-	3
	Netherlands	-	1
	Venezuela	-	1
	Unknown	-	53
	**Total Andigena**		**675**
**Chaucha**	Colombia	Nariño	32
	Peru	-	1
	Unknown	-	16
	**Total Chaucha**		**49**
**Phureja**	Colombia	Nariño	40
		Cauca	6
		V. del Cauca	2
		Antioquia	1
		Boyacá	1
		Cundinamarca	1
		Quindio	1
		N. de Santander	1
		Unknown	1
	Peru	-	6
	Bolivia	-	1
	United States	-	1
	Unknown	-	23
	**Total Phureja**		**85**
**TOTAL Colombian Central Collection**			**809**

### DNA extraction, genotyping and SNP markers selection

Fresh young leaves were collected from one plant randomly selected per accession. The material was lyophilized during two days at -50°C and 0.20 mBar. The genomic DNA was extracted using the DNeasy Plant Mini Kit (Qiagen, Valencia, CA, USA). DNA concentration and quality were checked by visualization in a 1% (w/v) agarose gel and a NanoDrop 2000 Spectrophotometer (Thermo Fisher Scientific, Wilmington, USA). Genotyping was performed using the array available in 2013, the Infinium 8303 potato SNP array [19, 21]. The array was read in the Illumina HiScan SQ system (Illumina, San Diego, CA) at CORPOICA. The software GenomeStudio version diploids and polyploids (Illumina, San Diego CA) was used to assign the genotype to each locus; five possible genotypes (AAAA, AAAB, AABB, ABBB or BBBB) in tetraploid potatoes and three possible genotypes (AA, AB and BB) in diploid potatoes. The assignation of samples as diploids through molecular markers was confirmed with the available information of cytogenetic analysis made in Phureja and Chaucha samples of the CCC reported by Guevara [36] and Uribe [37]. The SNPs that could not be called or were monomorphic were discarded. The remaining SNPs were filtered for up to 20% missing data and a Minor Allele Frequency (MAF) lower than 0.05. Genotypic data is provided in the S2 Table.

### Population structure and genetic differentiation

The population structure analysis was performed using a Bayesian model implemented in the software Structure [38] without *a priori* population information using a tetraploid model (Andigena: 1 = AAAA, 2 = AAAB, 3 = AABB, 4 = ABBB, 5 = BBBB; Phureja: 1 = AA, 3 = AB, 5 = BB). The analyses were conducted by varying the number of possible subpopulations (K) from 1 to 10, with five independent repetitions, assuming an admixture model with correlated allele frequencies and a burn-in of 50.000 and 150.000 iterations. The optimal number of subpopulations was established using the Evanno method [39] in Structure Harvester [40]. The number of subpopulations was confirmed with a Discriminant Analysis of Principal Component (DAPC) [41] conducted in the package Adegenet [42] in the R software [43] and a Principal Component Analysis (PCA) in the Tassel software [44]. Coefficients of genetic differentiation among subpopulations (F_ST_) and population inbreeding (F_IS_) within subpopulations were estimated by an analysis of molecular variance (AMOVA) with 1023 permutations in the Arlequin software [45]. The gene flow or number of migrants (*Nm*) was estimated through the equation: *Nm* = (1-F_ST_)/4F_ST_.

### Genetic diversity and cluster analysis

The genetic indexes, Observed Heterozygosity (Ho) and Expected Heterozygosity (He) were calculated using Genalex software [46]. The Polymorphic Information Content (PIC) was calculated using PowerMarker software [47] and the deviation from the Hardy-Weinberg equilibrium (HWE) was calculated using Genepop software [48]. Nei’s distances matrices [49] were calculated using the package StAMPP [50] in the R software [43] and the dendograms were constructed using the software PHYLIP [51] selecting the Neighbor-Joining (NJ) method with 1000 bootstrap replicates.

### Morphological characterization and correlations among morphological, geographical and genetic data

Phenotypic data from fifteen qualitative characteristics of stem, berry, tuber, and flower were used for the morphological analysis (Table 2). The Plant Genetics Resources team of CORPOICA recorded this information in eight different years (1995, 1996, 1997, 2004, 2006, 2009, 2010 and 2012) in 624 Andigena accessions using the descriptors of the CIP to characterize native potatoes [52]. The collection was evaluated in three different locations over field condition in Zipaquira (5° 03” 34.36” N, 74° 03” 29.61 W), Tibaitata (4° 41” 43.2” N, 74° 12” 13.3 W), and San Jorge (6° 01” 50.74” N, 74° 02” 40.65 W). Sixteen plants per accession were grown during eight months, time required to present structures to characterize. One plant per accession was randomly selected, and data was registered for each descriptor in five different berries, stems and flowers. Finally, tuber descriptors of five tubers per accession were registered after harvest. Phenotypic data for 624 Andigena accessions are presented in the S3 Table. The mode values of all variables for each accession were used to conduct a Multiple Correspondence Analysis (MCA) and a cluster analysis based on the Gower’s distance and the Ward method implemented in the software InfoStat [53]. The available passport data of 691 accessions of the CCC was used to generate the geographical distances between accessions in the Geographical Distances Matrix Generator software (http://biodiversityinformatics.amnh.org). The correlation between geographical, morphological and genetic distances was estimated by a Mantel test [54] with 1000 permutations in the software Genalex [46]. The correlations between morphological and genetic data were independently estimated for each variable. Subsequently, the global correlation was first calculated using the total of variables, and then using only positive and significant correlated variables.

**Table 2:** Results of morphological analysis of qualitative characters of Andigena population of the CCC.

Variable	Variable abbreviation	Coding	Multiple Correspondence Analysis (MCA)			Correlation with genetic distance	
			Dimension 1 (4.59%)	Dimension 2 (4.18%)	Dimension 3 (3.53%)	Percentage (%)	*p-value*
Primary Flower Intensity Color	PFIC	0–3	0.081	0.155	0.019	18	0.001*
Primary Flower Color	PFC	1–8	0.084	0.159	0.016	12.9	0.001*
Primary Tuber Skin Intensity Color	PTSIC	0–3	0.166	0.023	0.001	12.8	0.001*
General Tuber Shape	GTS	1–8	0.035	0.018	0.027	9.4	0.001*
Distribution of Secondary Flower Color	DSFC	0–9	0.075	0.119	0.059	9	0.01*
Primary Tuber Skin Color	PTSC	1–9	0.128	0.089	0.086	8.5	0.001*
Distribution of Secondary Tuber Skin Color	DSTSC	0–7	0.097	0.023	0.269	7.5	0.001*
Secondary Tuber Skin Color	STSC	0–9	0.094	0.059	0.271	7.1	0.002*
Berry Color	BC	1–7	0.014	0.056	0.022	5.7	0.001*
Distribution of Secondary Tuber Flesh Color	DSTFC	0–7	0.024	0.064	0.023	-5.3	0.026*
Stem Color	ST	1–7	0.094	0.04	0.112	-6.4	0.001*
Secondary Tuber Flesh Color	STFC	0–8	0.029	0.082	0.006	-8.4	0.003*
Primary Tuber Flesh Color	PTFC	1–8	0.005	0.002	0.033	3.5	0.114
Berry Shape	BS	1–7	0.003	0.007	0.014	0.4	0.431
Secondary Flower Color	SFC	0–8	0.071	0.103	0.04	-0.8	0.425

* Significance at *p* < 0.05

### Linkage disequilibrium

The linkage disequilibrium (LD) was calculated in each inferred population. The SNPs used presented the physical position (mapped) on the potato genome version 4.03 [55]. To include all SNP dosage (heterozygous genotypes), diploid and tetraploid data were analyzed following the report by Vos et al. [56], using the Pearson correlation coefficient between each pair of SNP marker. The LD decay was estimated using a combination of SNP markers in significant correlation (*p <* 0.001) with a threshold of *r*^*2*^ that corresponded to 90^th^ percentile [56] of pairwise correlations of each population.

### Association mapping analyses

Phenotypic data corresponding to 15 qualitative variables (Table 2, S3 Table) of 466 tetraploid accessions of Andigena accessions were used to identify marker-trait association using Mixed Linear Model (MLMs) analyses accounting for the population structure and kinship as fixed effects using the package GWASpoly [27] in the R software [43]. Additionally, the Andigena genotypic data was filtered with the default parameters of GWASpoly [27] (5% of missing data and a MAF of 0.10). To identify the SNPs with significant associations, the *p* values were corrected with the Bonferroni method [57] at *p* values of 0.05, 0.01 and 0.001.

## Results

### Genetic molecular analyses

The 809 accessions of the CCC were genotyped with 8303 SNPs using the Infinium SolCAP, 1584 markers were removed from the dataset since 1174 were monomorphic (14.1%), 405 SNPs could not be called (4.9%), and five presented more than 20% of missing data (0.1%). Genotype calling inferred 6719 high confidence SNPs (81%), from which 751 SNPs presenting a MAF less than 0.05, were also excluded, giving a total of 5968 useful markers (72%) (Table 3). Of these markers, 5790 were mapped on 12 chromosomes of the potato genome and 97 mapped on unanchored scaffolds (Chr. 0). Therefore, an average of 483 markers mapped on potato chromosomes ranging from 347 markers for Chr. 12 to 646 for Chr. 4.

**Table 3:** Genetic diversity statistics of the Colombian Central Collection of *S*. *tuberosum* group Andigenum.

Population	Subpopulation	N	Polymorphic markers	Ho (Mean +/- SD)	He (Mean +/- SD)	PIC (Mean +/- SD)	HWE
**CCC**	**Phureja**	133	3950 (66.2%)	0.194 (0.003)	0.167 (0.002)	-	266 (7.88%)
	**Andigena**	676	5951 (99.7%)	0.516 (0.004)	0.337 (0.002)	-	73 (1.22%)
	**Total**	**809**	**5968 (100%)**	**0.355 (0.003)**	**0.252 (0.002)**	**0.437 (0.191)**	**-**
							
**Phureja**	**Phureja_1**	90	2775 (99.86%)	0.387 (0.004)	0.339 (0.003)	-	5 (0.18%)
	**Phureja_2**	19	1516 (54.55%)	0.545 (0.009)	0.272 (0.005)	-	0 (0%)
	**Phureja_3**	24	1064 (38.29%)	0.380 (0.008)	0.190 (0.005)	-	0 (0%)
	**Total**	**133**	**2779 (100%)**	**0.437 (0.005)**	**0.267 (0.002)**	**0.279 (0.090)**	**-**
** **	** **						
**Andigena**	**Andigena_1**	24	3742 (63.41%)	0.628 (0.006)	0.315 (0.003)	-	50 (0.84%)
	**Andigena_2**	64	5144 (87.17%)	0.498 (0.005)	0.304 (0.003)	-	1 (0.03%)
	**Andigena_3**	170	4670 (79.14%)	0.471 (0.005)	0.285 (0.003)	-	1 (0.03%)
	**Andigena_4**	138	5900 (99.98%)	0.601 (0.003)	0.389 (0.002)	-	14 (0.32%)
	**Andigena_5**	280	5758 (97.58%)	0.476 (0.004)	0.304 (0.002)	-	7 (0.15%)
	**Total**	**676**	**5901 (100%)**	**0.535 (0.002)**	**0.319 (0.001)**	**0.269 (0.108)**	**-**

N: Number of samples, Ho: Observed Heterozygosity, He: Expected Heterozygosity, PIC: Polymorphic Index Content, HWE: Hardy-Weinberg equilibrium: SNPs not in HWE, SD: Standard deviation.

### Population structure and genetic diversity in the Colombian Central Collection

**Fig 2:**
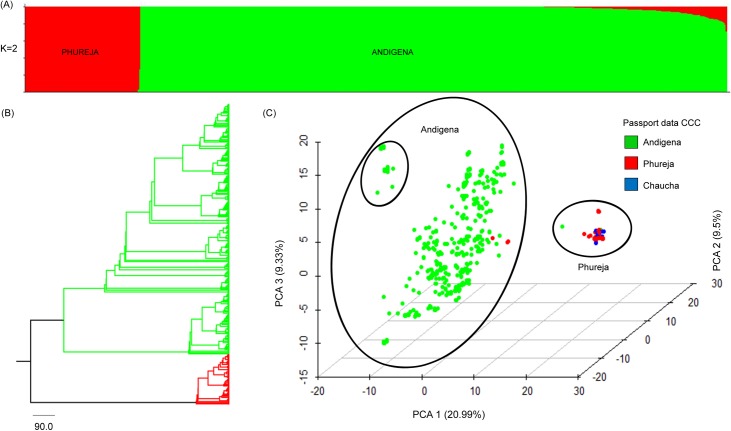
Population structure of 809 accessions of *S*. *tuberosum* group Andigenum. (A) Clustering Structure analysis. (B) NJ-tree based on Nei´s genetic distances. (C) Principal Component Analysis.

The population structure analysis of the CCC using the software Structure discriminated two main populations (K = 2) (Fig 2A, S1A Fig). The previous result was supported by Neighbor-Joining clustering analysis (Fig 2B) and the Principal Component Analysis in which 25.5% of variability was explained by the three first components (Fig 2C). The first population, named as Phureja, contains 133 accessions (16.4% of the CCC) from which 82 accessions have passport data and are classified as Phureja, two as Andigena (And_4 and And_183) and 49 as Chaucha. The majority of accessions of the CCC (83.6%) constituted the second population, named as Andigena, which regrouped 673 accessions with passport data of Andigena and three of Phureja (Phu_47, Phu_119 and Phu_122) (Table 3, S1 Table). The percentage of polymorphic SNPs was 66.2% and 99.7% for Phureja and Andigena populations, respectively (Table 3).

The genetic differentiation between Phureja and Andigena populations was high (F_ST_ = 0.203, *p* = 0.000), and the percentage of genetic variation was higher within populations (81%) than among populations (19%) (Table 4). High values of genetic variation within populations imply high genetic diversity. The CCC presented an excess of heterozygosity (F_IS_ = -0.517, *p* = 1.000) and a low gene flow (*Nm* = 0.98) (Table 4). High genetic diversity was found in the CCC (Ho _CCC_ = 0.355, He _CCC_ = 0.252), where the genetic diversity was higher in Andigena (Ho = 0.516, He = 0.337) than Phureja (Ho = 0.194, He = 0.167) (Table 3). Observed population structure supported the passport data that differentiates two main groups, Andigena and Phureja. Samples included in the Phureja population were characterized by presenting a chromosome number of 24 (2n = 2x) determinate previously by Guevara [36] and Uribe [37]. Because of the difference in ploidy level, the two populations were analyzed independently.

**Table 4:** Analysis of Molecular Variance (AMOVA) based on SNP markers for each population of *S*. *tuberosum* of the Colombian Central Collection.

Population	Source of variation	Degrees of freedom	Sum of squares	Mean square	Variance component	Percentage of variation (%)	F-statistics	*p-value*	*Nm*
**CCC**	**Among populations**	1	99887.3	99887.3	94.295	19	**F**_**ST**_ = 0.203	0.000	-
	**Within populations**	1616	1569442.1	917.1	1011.791	81	**F**_**IS**_ = -0.517	1.000	-
	**Total**	**1617**	**1669329.4**		**1106.086**	**100**	-	**-**	0.98
**Phureja**	**Among populations**	2	17322.3	8661.1	126.446	23	**F**_**ST**_ = 0.225	0.000	-
	**Within populations**	263	114539.9	435.5	435.513	77	**F**_**IS**_ = -0.342	1.000	-
	**Total**	**265**	**131862.3**		**561.959**	**100**	-	**-**	0.86
**Andigena**	**Among populations**	4	66274.5	15639.3	65.7	6.5	**F**_**ST**_ = 0.06	0.000	-
	**Within populations**	1161	1240882.0	990.0	938.7	93.5	**F**_**IS**_ = -0.59	1.000	-
	**Total**	**1165**	**1307156.5**		**1004.4**	**100**	-	**-**	3.91

*Nm*: Gene flow or Number of migrants.

#### Phureja population

**Fig 3:**
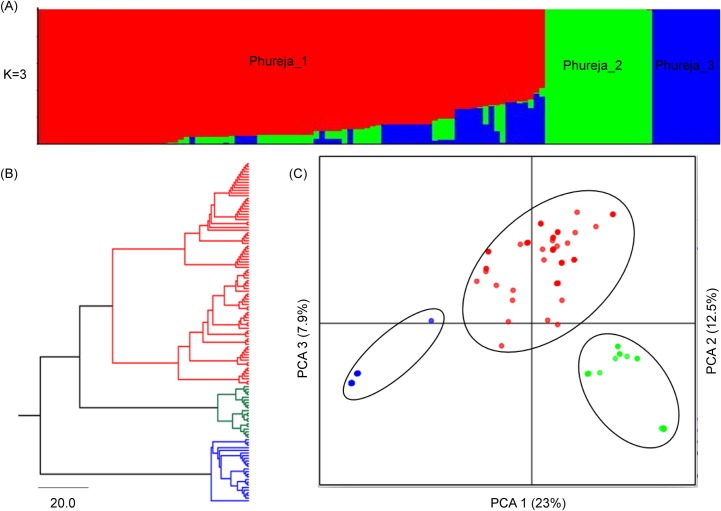
Population structure of 133 diploid accessions (Phureja population) of Colombian Central Collection of *S*. *tuberosum*. (A) Clustering Structure analysis. (B) NJ-tree based on Nei´s genetic distances. (C) Discriminant Analysis Principal Component.

In the Phureja population, 2779 SNPs had a MAF value higher than 0.05 and passed missing data filters. The accessions of Phureja were clustered in three subpopulations (K = 3) (Phureja_1, Phureja_2 and Phureja_3) (Fig 3A, S1B Fig). The simulations from the software Structure were consistent with the NJ-tree (Fig 3B) and the DAPC analysis (Fig 3C), where 43.5% of the variation was explained by the three first components of the PCA. The three Phureja subpopulations differed genetically among them (F_ST_ = 0.225, *p* = 0.000), and were characterized by presenting an excess of heterozygotes (F_IS_ = -0.342, *p* = 1.000) and low gene flow (*Nm* = 0.86) (Table 4). The genetic differentiation was supported by significant F_ST_ values (*p* = 0.000) observed among the subpopulations that ranged from 0.161 (Phureja_1 vs. Phureja_2) to 0.435 (Phureja_2 vs. Phureja_3) (S4 Table). The distribution of genetic variation within and among subpopulations estimated by AMOVA indicated that 77% of the total genetic variation was found within subpopulations and 23% among subpopulations (Table 4). The population Phureja presented high genetic diversity with an average Ho of 0.437, He of 0.267 and PIC of 0.279 (Table 3).

#### Andigena population

**Fig 4:**
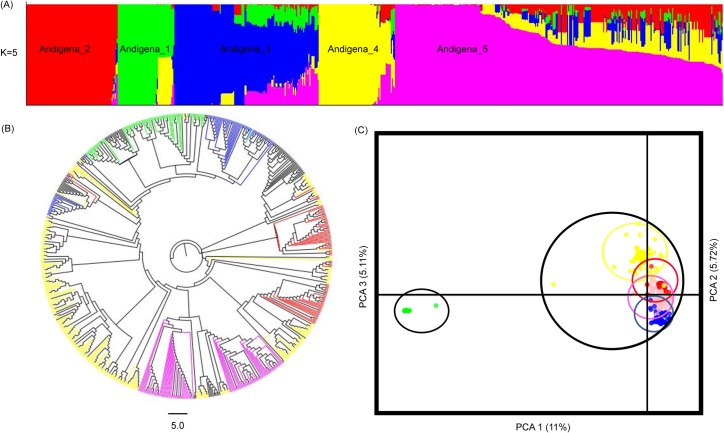
Population structure of 676 tetraploid accessions (Andigena population) of Colombian Central Collection of *S*. *tuberosum*. (A) Clustering Structure analysis. (B) NJ-tree based on Nei´s genetic distance. (C) Discriminant Analysis Principal Component.

A total of 5901 SNPs (MAF > 0.05) were polymorphic in Andigena population and the analyses conducted on these data subdivided the Andigena population in five groups (K = 5) (Andigena_1—Andigena_5) (Fig 4 and S1C Fig). The inferred groups in the structure analysis were not clearly separated by the cluster analysis (Fig 4B) and the DAPC, where the three first components of the PCA only explained the 20.7% of the variation. An unique subpopulation (Andigena_1) was genetically differentiated of the other four subpopulations (Fig 4C; S2 Table). The AMOVA showed that genetic variation was higher within subpopulations (93.5%) than among subpopulations (6.5%), with a population with low genetic structure (F_ST_ = 0.06, *p* = 0.000), excess of heterozygosity (F_IS_ = -0.59, *p* = 1.000) and high gene flow (*Nm* = 3.91) (Table 4). The F_ST_ values (*p =* 0.000) of Andigena_2 to Andigena_5 subpopulations were low, ranging from 0.031 (Andigena_3 vs. Andigena_5) to 0.080 (Andigena_2 vs. Andigena_3), and high among these subpopulations with Andigena_1 that ranged from 0.122 (Andigena_1 vs. Andigena_5) to 0.216 (Andigena_1 vs. Andigena_3) (S4 Table). The Andigena population presented a high genetic diversity with averages of Ho = 0.535, He = 0.319 and PIC = 0.269 (Table 3).

### Morphological characterization of Andigena population

The MCA based on morphological traits among 624 Andigena accessions showed that the total morphological variation was distributed in 73 dimensions, from which the three first dimensions explained the 12.3% of the variation (Table 2). The first dimension was provided by tuber variables as shape (GTS), color (PTSC) and primary skin intensity color (PTSIC). The second by berry color (BC), secondary color (STFC) and distribution of tuber flesh (DSTFC) and all variables related with flower (PFC, PFIC, SFC and DSFC). Finally, the primary tuber flesh color (PTFC), secondary color (STSC) and distribution of skin tuber (DSTSC) and variables related to stem color (SC) and berry shape (BS) contributed to the variation of the third dimension (Table 2).

**Fig 5:**
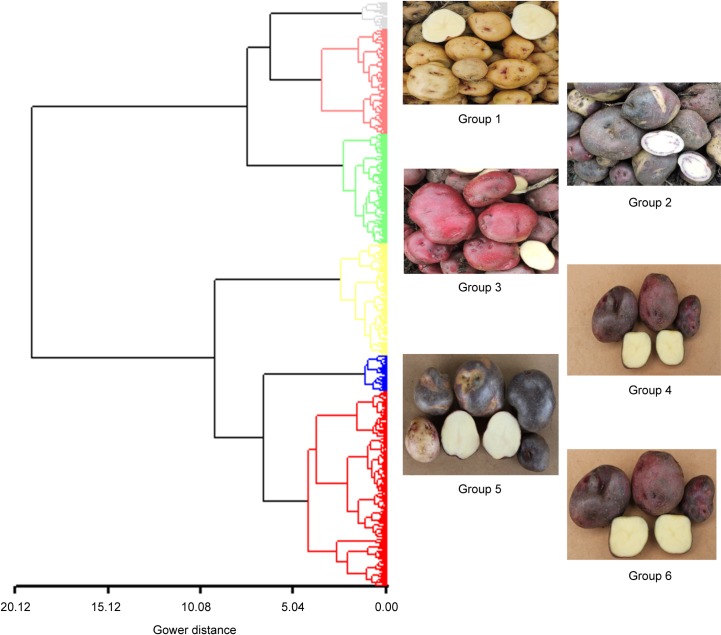
Dendrogram generated by the Ward method and Gower distances between 624 tetraploid accessions (population Andigena) of the Colombian Central Collection of *S*. *tuberosum* based on qualitative morphological data.

The cluster analysis discriminated six morphological groups within the Andigena population (Fig 5). Although all the groups presented flesh tubers cream, in every group the largest proportion of accessions was characterized by specific tuber traits (S5 Table). Group 1 (108 accessions) is characterized to present compressed tubers with pale yellow skin and purple dots. Group 2 (59 accessions) had compressed tubers with dark purple skin, sometimes with scattered yellow spots and flesh cream color with secondary purple color distributed in narrow vascular ring. Group 3 (119 accessions) had compressed tubers with dark red skin. Group 4 (32 accessions) had compressed tubers with pale purple skin. Group 5 (159 accessions) had round tubers with dark purple skin with scattered yellow spots. Finally, compressed tubers with pale purple skin and yellow scattered spots are characteristics of group 6 (147 accessions). Group 4 presented white flowers while the other groups presented dark purple flowers.

### Correlations among morphological, geographical and genetic data

The Mantel test showed no correlation between geographical distribution and morphological (1.2%, *p* = 0.311), and geographical distribution and genetic data (4.2%, *p* = 0.111). However, a low but significant correlation (13.2%, *p* = 0.001) was identified between all morphological variables analyzed and the genetic data (Table 5). Additionally, the correlation analysis was implemented for each morphological variable, independently. Within the 15 variables used, three (BS, PTFC and SFC) were not correlated (*p* > 0.05), three (SC, STFC and DSTFC) were negatively correlated (*p* < 0.05) and the remaining nine were positively correlated (*p* < 0.05). The variables with higher correlation were those related with flower variables (PFIC: 18.0%, PFC: 12.9%, DSFC: 12.8%), general tuber shape (GTS: 9.4%) and primary (PTSIC: 12.8%, PTSC: 8.5%) and secondary color of skin tuber (DSTSC: 7.5%, STSC: 7.1%) (Table 2). The global correlation between morphological and genetic data using only variables significantly correlated was of 21.6% (*p* = 0.001) (Table 5). Although this correlation was low, the subpopulations identified using molecular markers were characterized by presenting tuber traits in common. For instance, the tuber skin primary color of Group 1 and group 2 is dark purple, group 3 is pale yellow, group 4 is pale purple and group 5 is dark red. However, morphological and genetic groups did not completely match.

**Table 5:** Correlations between genetic, geographical and morphological distances in populations of the Colombian Central Collection of *S*. *tuberosum*.

Analyses	Percentage of correlation (%)	*p-value*
Phenotypic_distance_Andigena/Geographic_distance_Andigena	1.20	0.311
Genetic_distance_CCC/Geographic_distance_CCC	4.20	0.111
Genetic_distance_Andigena/Phenotypic_distance_Andigena (All variables)	13.2	0.001*
Genetic_distance_Andigena/Phenotypic_distance_Andigena (variables correlated)	21.6	0.001*

* Significance at *p* ≤ 0.05 at 1000 permutations.

### Linkage disequilibrium

The linkage disequilibrium between pairwise SNPs was estimated for Phureja and Andigena populations; the analysis showed that the amount of SNPs in LD and the extent of LD differed among these.

#### Linkage disequilibrium in Phureja

The LD in Phureja was estimated using data from the entire population (133 accessions) and separately for the subpopulation Phureja_1. The analysis was not conducted in the subpopulations Phureja_2 and 3, because they presented a low number of samples. In this analysis the 2555 markers used, mapped on the 12 chromosomes of the genome, with a mean distance between markers of 22.7 Mb, ranging from 11.5 Mb (Chr. 2) to 34.2 Mb (Chr. 1). The Pearson *r*^*2*^ values for the 133 Phureja accessions were 0.463 for linked markers with 49.8% of the markers in significant LD. The *r*^*2*^ values ranged from 0.440 (Chr. 1) to 0.496 (Chr. 12) (Table 6). The pairwise correlations among linked markers in significant LD (*p* < 0.001) were used to assess the extension of LD decay. The threshold for *r*^*2*^ was 0.45 representing the 90^th^ percentile of all pairwise correlations in the Phureja population. Using this threshold, the LD declined to 3.5 Mb for linked markers in the population Phureja. For each chromosome of the potato genome the LD decay was estimated and ranged from 2 Mb (Chr. 1, 4, 11) to up to 9 Mb (Chr. 3, 12) (Table 6).

**Table 6:** Linkage disequilibrium in populations of the Colombian Central Collection of potato.

	Phureja population					Andigena population				
Chr^A^	Number of Markers	Mean distance (Mb)	*r*^*2*^	Significant LD (%)^B^	LD decay (Mb)^C^	Number of markers	Mean distance (Mb)	*r*^*2*^	Significant LD (%)^B^	LD decay (Mb)^C^
**1**	340	34.2	0.440	49.4	2	553	31.6	0.242	50.5	0.8
**2**	192	11.5	0.464	49.2	4	403	9.5	0.257	50.3	1.8
**3**	167	21.7	0.484	49.1	9	351	11.9	0.247	50.7	1
**4**	354	24.9	0.446	49.4	2	537	17	0.234	50.7	0.3
**5**	156	21.6	0.487	50.1	5.5	350	16.5	0.252	50.3	0.4
**6**	225	23.5	0.486	50.2	8	411	18.6	0.280	50.0	4
**7**	141	28.2	0.477	49.0	4.5	432	23.5	0.277	49.9	4
**8**	186	19.2	0.469	49.2	6	349	15.1	0.283	50.4	8
**9**	252	24.8	0.468	48.7	7	439	20.3	0.239	50.1	0.4
**10**	202	23.3	0.468	49.9	3.5	318	16.6	0.263	50.3	1.7
**11**	192	17.8	0.466	49.8	2	309	13.1	0.267	50.8	0.8
**12**	148	21.4	0.496	49.7	9	291	21.6	0.255	50.0	0.5
**Linked markers**	2555	22.7	0.463	49.8	3.5	4743	17.9	0.256	50.1	0.8

^A^ Chromosome Number

^B^ Significant threshold is set to *p* < 0.001

^C^ Threshold in 90^th^ percentile (Phureja: *r*^2^ = 0.45; Andigena: *r*^2^ = 0.25).

#### Linkage disequilibrium in Andigena

The LD of the Andigena population was calculated using 4743 molecular markers distributed over the 12 chromosomes identified in 652 accessions corresponding to the Andigena subpopulations except for subpopulation Andigena_1. In Andigena population the SNPs mapped on the chromosomes had a mean distance between markers of 17.9 Mb ranging from 9.5 Mb (Chr. 2) to 31.6 Mb (Chr. 1). The LD was not estimated for each subpopulation independently, because these groups did not differ genetically. The subpopulation Andigena_1 was excluded of the analyses because it presented a high genetic differentiation from the others. In addition, the LD in this subpopulation was not independently assessed because it was represented by a low number of samples. The average Pearson *r*^*2*^ values obtained was 0.256 for linked markers with 50.1% combinations of markers in significant LD. The mean *r*^*2*^ value in the 12 chromosomes ranged from 0.234 (Chr. 4) to 0.283 (Chr. 8). To estimate the LD decay in the Andigena population, the *r*^*2*^ threshold was 0.25 representing the 90^th^ percentile of the all pairwise Pearson correlations. The extent of LD was 0.8 Mb in linked markers and every chromosome ranged from 0.3 Mb in chromosome 4 to 8 Mb in chromosome 8 (Table 6).

### Association mapping analyses

The marker-phenotype association analysis was implemented using 4666 polymorphic SNPs of 463 tetraploid accessions of the CCC. A complete dataset of the phenotypic variables was available for these accessions. A total of 23 markers with log_10_ (*p-value*) ranging between 4.6 for STFC (solcap_snp_c1_12945) and 9.36 for PFC and PFIC (solcap_snp_c2_43970), were significantly associated with 9 of the 15 evaluated variables (Table 7). In addition, seven markers presented significant *p* values less than 0.01 and four had *p* values less than 0.001. Of these four markers, three (solcap_snp_c2_45693, solcap_snp_c2_23347 and solcap_snp_c2_43970) were associated with PFC and PFIC and one (solcap_snp_c2_45235) with STFC (Table 7).

**Table 7:** List of SNPs associated to qualitative data in tetraploid accessions of *S*. *tuberosum* of the Colombian Central Collection.

Variable	Variable abbreviation	Marker	Chromosome	Position (bp)	log_10_(*p-value*)	Model^1^	Significance
**Stem Color**	**ST**	solcap_snp_c2_46710	7	4874743	5.16	SD	*
		solcap_snp_c2_36061	4	58752313	5.09	DD	*
**Primary Tuber Skin Intensity Color**	**PTSIC**	solcap_snp_c2_21750	2	25053972	5.29	AD	*
		solcap_snp_c2_51533	7	53656225	5.05	DD	*
**Distribution of Secondary Tuber Skin Color**	**DSTSC**	solcap_snp_c2_45235	10	58437496	6.62	AD	***
**Primary Tuber Flesh Color**	**PTFC**	solcap_snp_c2_12578	7	53077509	4.75	SD	*
**Secondary Tuber Flesh Color**	**STFC**	solcap_snp_c2_35705	2	47327646	5.33	SD	**
		solcap_snp_c1_12945	4	57899405	4.6	SD	*
**Distribution of Secondary Tuber Flesh Color**	**DSTFC**	solcap_snp_c2_15070	2	45695772	4.64	SD	*
**General Tuber Shape**	**GTS**	solcap_snp_c2_26014	7	50155390	4.74	DD	*
**Primary Flower Color**	**PFC**	solcap_snp_c2_24563	12	243727	4.76	SD	*
		solcap_snp_c1_5388	3	3161621	4.94	DD	*
		solcap_snp_c2_23355	7	42119766	5.5	DD	**
		solcap_snp_c2_46329	7	48224240	6.36	DD	**
		solcap_snp_c1_9878	7	50960978	5.86	DD	**
		solcap_snp_c2_45693	10	51357092	6.53	DD	***
		solcap_snp_c1_16169	1	44194124	5.29	DD	*
		solcap_snp_c2_23347	7	42120645	7.01	DD	***
**Primary Flower Intensity Color**	**PFIC**	solcap_snp_c1_4464	5	32104744	5.53	DA	**
		solcap_snp_c2_45701	3	43326802	5.19	DD	*
		solcap_snp_c2_42348	6	36881223	5.88	DD	**
**Primary Flower Color / Primary Flower Intensity Color**	**PFC/PFIC**	solcap_snp_c2_36468	3	38153740	5.87/4.85	DA/SD	**
		solcap_snp_c2_43970	1	13545189	9.36/5.29	DD	***

^1^ Model with the most significant marker is listed. AD = additive, SD = simplex dominant, DD = duplex dominant, DA = diplo—additive. Significant

* < 0.05

** < 0.01

*** <0.001

## Discussion

The growth in food demand and climate change raised the necessity to generate crop varieties having higher yield and adapted to a changing environment [58]. It is fundamental to plant breeding to characterize the genebank collections because the genetic improvement of economically important traits depends on the genetic diversity available within the crop species and its wild relatives [59, 60]. Modern elite gene pools could be created exploring the genetic resources conserved in large ex situ germplasm collections to identify genes of interest and allelic diversity [61, 62]. Highly polymorphic molecular markers could be identified in diverse germplasm that could be effectively used for mapping genes or QTLs [62] to assist plant breeding programs.

In Colombia, the CCC contains potato accessions coming from different Colombian regions and several countries. Researchers from CORPOICA had selected accessions from the CCC presenting valuable traits such as resistance to drought, to several diseases and to insect pests. Information about the genetic diversity and population structure of the CCC and the identification of molecular markers related to traits of interest for potato breeding could speed up the selection for desirable traits. So far, only one study of the genetic diversity and population structure of the CCC-Universidad Nacional de Colombia has been published [18]. The analysis included only 97 diploid accessions, from which few are in common with the CCC-CORPOICA [18]. The accession numbers of the CCC-Universidad Nacional de Colombia were modified and do not correspond to the accessions numbers of the CCC-CORPOICA, difficulting the comparison between studies. The present study is the first report using the majority of accessions of the CCC to assess its genetic variability, population structure and linkage disequilibrium. The information obtained will allow the implementation of association-mapping studies to this collection.

### Genetic analyses

The development of SNP arrays using high-throughput technology has allowed to genotype germplasm of crops such as potato [20, 19], tomato [63], barley [64], rice [65] among others. In this study, the Infinium SolCAP 8K was used to genotype accessions of the CCC, providing informative data with 72% of polymorphic loci. Previous studies in potato germplasm of other collections reported similar level of polymorphism using the same array: 77% [9], 74% [22], 61% [23], 67% [25], and 76% [66]. A degree of ascertainment bias could be expected when the SolCAP 8K is used to analyze populations such as the Colombian potato germplasm because it was designed based on transcriptome data and EST databases of North American cultivars [19, 21]. However, the high percentage of polymorphism suggested that the array provided enough markers representing the allelic composition of the CCC compared to previous works in other germplasm using the same array [22, 23]. A high number of polymorphic markers was expected due to the significant number of samples included [20].

This paper presents a robust analysis of the genetic diversity of CCC using a high number of molecular markers distributed on the 12 chromosomes of the potato genome. A previous genetic study using only 97 diploid accessions and 42 SSR covered a small amount of the potato genome, with a mean coverage of three markers per chromosome [18]. In general, the highest proportion of genetic studies in potato have used techniques that produced few molecular markers such as SSR [67–69], AFLPs [34, 59, 70] and RAPDs [71–73]. Each type of molecular marker provides information not always comparable because some have a biallelic and others a multiallelic nature [7, 74]. However, the estimation of the genetic variability of a population improves as the number of markers increase [75]; the SolCAP 8K could then provide a better assessment of the genetic variability of the CCC.

### Population structure and genetic diversity in the Colombian Central Collection

In this study, the molecular markers were useful to identify mislabeled accessions [7, 23]; some accessions of Andigena and Phureja did not clustered according to their passport data. The impossibility to identify two different populations of Phureja and Chaucha suggested an error of classification in the CCC. According to Guevara [36], accessions of the CCC labeled as Chaucha are not triploids as expected but diploids (2n = 2x = 24) as Phureja accessions [37]. Hence these accessions were probably misclassified as Chaucha, being in fact Phureja. The misclassification of accessions and errors in the assignment of samples to corresponding group in the CCC could have several explanations. The common name used by farmers for the same type of potato probably changes from region to region. For example, in the state of Nariño in Colombia farmers use the name Chauchas for potatoes similar to Phurejas. Another explanation could be a hybrid origin of these accessions; natural hybridization occurs between varieties in cultivated areas because potato farmers do not cultivate the varieties separately [4, 76].

#### Population structure and genetic diversity in Phureja and Andigena populations

The two inferred populations of CCC present high genetic diversity and were genetically differentiated with low gene flow among them, probably due to the difference in ploidy level [35]. The SolCAP array was also able to differentiate European [22] and American [23] potatoes by their ploidy level. The diploid population (Phureja) had high genetic differentiation, all the multivariate analyses supported the presence of the three subgroups and genetic admixture was no identified. In fact, the results showed a low gene flow, suggesting a strong genetic differentiation, given that *Nm* is inversely proportional to the genetic differentiation among populations [77]. Human selection (e.g. breeders, farmers) to color and quality of tuber probably played an important role shaping the current population structure of group Phureja. However, it is necessary to conduct a morphological evaluation of Phureja potatoes of the CCC in order to support this hypothesis. The results obtained from Phureja population contrasted to the reported in the study of Juyó et al. [18], who identified a moderate population structure (F_ST_ = 0.09), a high gene flow (*Nm* = 1.61) and only 9.64% of the variation among populations in diploid accessions of CCC-Universidad Nacional de Colombia. These two studies differed in the molecular markers (number and type) and samples (number and origin) evaluated. Samples analyzed in the two works were not exactly the same. Although the CCC-Phureja from the Universidad Nacional de Colombia conserves part of the accessions of the CCC-CORPOICA, the ID numbers did not match. In addition, some accessions of the CCC-Universidad Nacional were recently collected. Juyó et al. [18] used SSR markers, which are considered more efficient than SNP markers to identify subpopulations, because they are neutral and more alleles can be identified [78–79]. However, the high number of SNPs markers used in this study allowed to identify three populations in Phureja accessions. The population structure is influenced by the joint effects of many factors including the mating system, natural and artificial selection, mutation, migration and dispersal mechanism, drift, etc. [80, 81]. In potato, the selection of potatoes by farmers and breeders presenting characteristics such as high yield, large tubers, low glycoalkaloid levels, desirable flavor, short cooking times and high nutritional value could affect the genetic structure [82–84].

Andigena population presents a genetic admixture supported by a high gene flow among populations [85]. The lack of population structure in tetraploid potatoes has been previously reported in other studies [35, 86–88] and has been explained by sexual polyploidization, intervarietal introgressive hybridization and long-distance dispersion [5, 89]. Although the whole Andigena population did not show a population structure, a cluster (Andigena_1) with samples probably belonging to the Tuberosum group could be identified. The *S*. *tuberosum* group *tuberosum* of CCC were probably originated from landraces and breeding material from United States and Europe [12]. Tuberosum potatoes differentiate from other Andigena potatoes by the formation of tubers in long days and by their adaptation of medium altitudes and subtropical weather from Europe, United States and Asia [8, 90].

High genetic diversity was found in both populations according with other studies [18, 68, 89]. In this work, the observed heterozygosity was higher than expected heterozygosity. Potato is an outcrossing species thus the proportion of inbreeding is expected to be low, thus the heterozygosity is higher than expected. The high diversity in potato is explained by its evolution shaped by selection, migration, mutation, hybridization, polyploidization and introgression. In the case of diploid potatoes, wild and cultivated species are often self-incompatible (SI) [91, 92]. Thus, potato genetics allow the production of heterozygote plants increasing the genetic variability [1, 35, 81]. The PIC values suggested that the SNPs of SolCAP are useful to analyze diploid and tetraploid accessions and could support the suggestion that genetic diversity in tetraploid potatoes has not been narrowed in spite of the commercial breeding efforts [10, 34]. Based on PIC values, the CCC (PIC = 0.437) is more diverse than European potatoes (PIC = 0.35), supporting the idea that South American potato populations are more diverse than European potatoes reported by Bornet et al. [93] and Esfahani et al. [94]. According to these results, the CCC has a broad genetic basis with alleles that could be profitable for plant breeding [21]. In fact, studies in diploid accessions of the CCC-Universidad Nacional de Colombia have already detected markers related to resistance to *Phytophthora infestans* [95], sugar content and frying color [96].

### Morphological characterization of Andigena population

The accessions of Andigena population showed wide phenotypic diversity based on fifteen morphological traits, in which shape, skin color and color intensity of tuber and flower attributes were the most informative variables to discriminate the six groups of Andigena. Previous works reported that the same variables were useful to differentiate potato accessions [97–99]. Variables describing the tuber are the most useful descriptors to select potatoes for breeding programs [100]. The dark color in skin and flesh tuber is an indicator of the presence of phenolic compounds which are considered health-promoting phytochemicals because of their antioxidant properties [101]. The CCC presents a wide variability in tuber colors indicating a potential source of accessions with high phenolic compounds levels; further characterization of content of biochemical compounds of the CCC is needed. Previously, Bernal et al. [97] analyzed morphologically 464 accessions of the CCC of potato. They found seven different groups instead of six groups and they identified higher morphological variability than the present study. However, the same traits were reported as informative in the two studies, and the samples were regrouped based on the same characters of tuber and flower. The difference in results between studies could be due to a smaller number of variables used in this study. Additionally, the data analysis made by Bernal et al. [97] was based on one year of morphological records. In contrast, the present study used morphological data recorded on eight different years. Our analyses identified that some descriptors changed over the years such as color and intensities of tubers and flowers. The lack of stability of morphological characters has been also reported in the evaluation of the CIP collection [5] suggesting that the selection of potato materials could not be only based on morphological data. The characterization and selection of potato accessions should be complemented with molecular data, reported to be more informative and neutral than phenotypic traits in establishing potato relationships [62].

### Correlations among morphological, geographical and genetic data

In this work, geographic distance was not correlated between genetic and morphological distances. Similar results were obtained in previous studies using potato collections for morphological data [102, 103] and molecular data [7, 104–106]. The lack of correlation is probably the result of tuber transportation by humans [107], caused by historical migrations of wild potato germplasm away from their regions of origin [23]. Morphological and genetic data were weakly correlated; similar results were found in other populations of potato [108–110]. The low correlation between genetic and morphological data is probably due to differences in selection pressure. Non-adaptive molecular markers are usually not subjected to natural or artificial selection while phenotypic characters are subjected to selection pressure and influenced by the environment [106, 111]. This result could explain why groups identified through molecular and morphological markers did not match.

### Linkage disequilibrium and association mapping analyses

The linkage between molecular markers and phenotypic polymorphisms is required for the association mapping of genes or QTLs underlying traits of interest [112]. The extent of LD can be affected by factors such as genetic drift, population structure and selection [113]. In association mapping studies, a key factor is to know the population structure in order to improve the statistical power and decrease the false positive rate in gene discovery [76]. The analysis of LD was independently conducted for Phureja and Andigena, where the LD levels varied between these. High levels of *r*^*2*^ and SNP pairs with significant LD in Phureja and Andigena were identified. These results contrasted with the study of Juyó et al. [18] in diploid potatoes in which no molecular markers in significant LD were detected, probably due to the number and type of markers used (SSRs). Additionally, the number of linked markers in LD was higher than unlinked markers as expected, thus physical linkage strongly influences LD. The results indicate that molecular markers found in CCC in this study are suitable for an association analysis [114].

To estimate the LD decay in Phureja and Andigena populations, a *r*^*2*^ threshold of 0.45 (Phureja) and of 0.25 (Andigena) were used. Those values corresponded to the 90^th^ percentile of the distribution of all pairwise Pearson correlation in each population. Vos et al. [56] found that percentiles of 90 or 95 are useful to estimate the LD in potato. The difference in cutoff used in previous studies (*r*^*2*^ = 0.1) did not allow the comparison among studies [22, 34, 35, 95]. However, the LD decay values obtained in this work in tetraploid potatoes were similar to the reported in the potato germplasm (0.6–1.5 Mb) analyzed by Vos et al. [56]. The *r*^*2*^ values and extent of LD through the genome differ among studies because of differences in population size, number and type of markers [115] and the regression methods used to measure the LD [116]. The polyploidy and outcrossing species generally exhibit low LD because of the recombination events, which occur more frequently in large and highly heterozygous populations [117]. In contrast, the self-pollinated crops usually display LD over larger distances as a consequence of their mating system [34]. Based on its LD value, potato behaves as a self-pollinated crop even if it is an outcrossing species. The clonal propagation of potato limits the number of meiotic generations and in consequence the recombination events [33–35, 118]. The LD in Andigena and Phureja decayed slowly, previous works also reported a slow LD decay for potato populations: 1 cM [33], 10 cM [35] and 5 cM [34]. It is not rare to found differences in values of LD decay among populations that have suffered different breeding history and human selection [119, 120].

The LD decay value is useful to design future GWAS studies; it makes possible to estimate the minimum number of SNPs required to have a successful GWAS [115]. Since Phureja and Andigena populations have a long range LD through the genome, with a physical genome length of 844 Mb [121, 122] and a genetic map length of 800 cM [123], association studies can be performed with a modest number of markers per unit of genetic distance, this inference in potato has been reported previously by D’hoop et al. [34] and Simko et al. [35]. The inferences about the association mapping in the CCC of potato were validated with the identification of molecular markers associated with the morphological traits. In a GWAS analyzing North American potatoes using the same array, molecular markers with minor effects were identified to be related to morphological data such as total yield, eye depth, tuber shape and tuber length [27]. In the present work, four of 23 associated markers presented *p* values less than 0.001. Of these four markers, three were associated with flower primary color and one with secondary color distribution in tuber skin. The marker solcap_snp_c2_45235 (Chr. 10, position: 58437496) was associated to secondary color and was mapped to the gene *Sotub10g021050*.*1*.*1* (PGSC0003DMG400008137) which has a glucosyltransferase function. Some glucosyltransferase enzymes are implicated in the production of anthocyanin, pigment compound of skin and flesh tubers [124]. In addition, the same SNP (solcap_snp_c2_45235) is located closed to two genes (PGSC0003DMG400013965, PGSC0003DMG400012891) associated to skin and flesh color of potato tuber, reported recently by Endelman and Jansky [125].

The SNP dataset produced in this study and the germplasm analyzed would allow the implementation of association-mapping studies and to detect markers or genes associated to traits of interest useful for potato breeding such as resistance to pathogens and insect pests, tolerance of abiotic stresses and tuber quality. The function of the associated markers should be validated through genetic transformation. Additionally, conventional potato plant breeding programs could be supported using the genetic information through marker-assisted selection (MAS) and genomic selection (GS), and thus to accelerate the selection of potato materials and reduce the cost and time to develop new potato varieties.

## Conclusion

The present study is the first report of phenotypic and genotypic evaluations of the Colombian Central Collection of *Solanum tuberosum* using morphological and SNP molecular markers. The study identified high levels of genetic diversity and genetic differentiation in diploid and tetraploid potatoes. CCC constitutes a potential source of variable traits useful for a genetic breeding program. Additionally, the linkage disequilibrium study of the CCC indicated that the genomes of Phureja and Andigena presented an elevated number of SNP pairs in significant LD and a slow LD decay, suggesting that with a modest number of molecular markers, a marker-phenotype association could be detected. The information obtained in this work allowed to conclude that the CCC is a germplasm with a broad genetic base and is useful to conduct association mapping studies suitable for the identification of QTLs/genes associated to quality traits and biotic and abiotic stress tolerance traits.

## Supporting information

S1 Fig Delta K inferred in each analyzed population. (A) Overall Colombian Central Collection. (B) Phureja population. (C) Andigena Population.
(TIF)

S1 Table List of accessions of the Colombian Central Collection of *S*. *tuberosum* group Andigenum and information of sample collection sites. (DOC)

S2 Table Genotypic data of 809 accessions of Colombian Central Collection of *S*. *tuberosum* group Andigenum obtained through Infinium technology. (XLSX)

S3 Table Phenotypic data of Colombian Central Collection of *S*. *tuberosum* group Andigenum (Andigena population). (XLSX)

S4 Table Pairwise genetic differentiation (F_ST_) values between populations of *S*. *tuberosum* in the Colombian Central Collection. (DOC)

S5 Table Summary of characteristics for the six groups identified by the morphological analysis in tetraploid accessions of the Colombian Central Collection of *S*. *tuberosum*. (DOC)
