# Supplementary figures and images for: Genetic diversity and association mapping in the Colombian Central Collection of Solanum tuberosum L. Andigenum group using SNPs markers
Source: PLoS One. 2017 Mar 3;12(3):e0173039. doi: 10.1371/journal.pone.0173039 (PMC5336250; doi:10.1371/journal.pone.0173039)

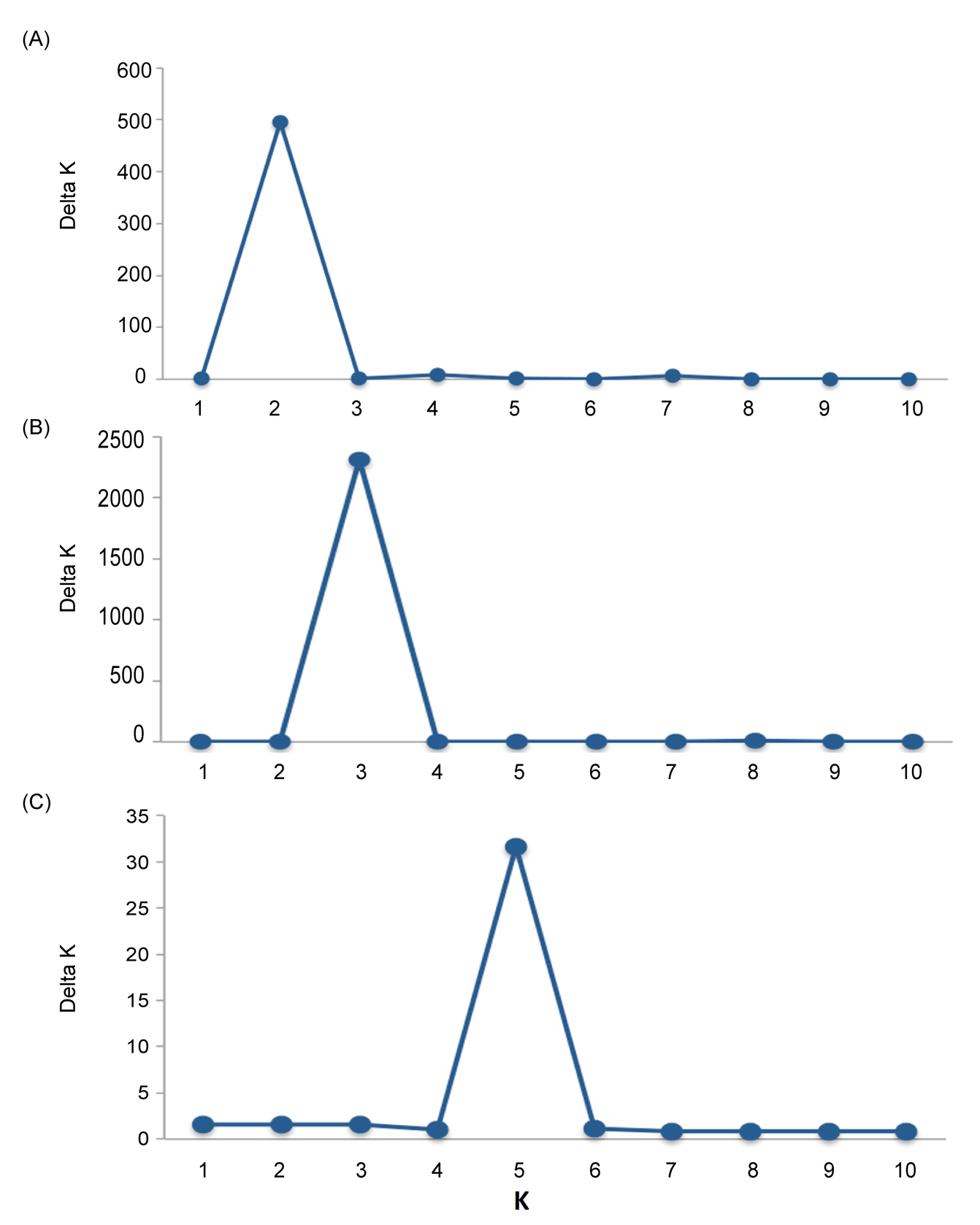

Supplement: S1 Fig — (A) Overall Colombian Central Collection. (B) Phureja population. (C) Andigena Population. (TIF) [file pone.0173039.s001.tif]
