# Supplementary material for: Genetic diversity and association mapping in the Colombian Central Collection of Solanum tuberosum L. Andigenum group using SNPs markers
Source: PLoS One. 2017 Mar 3;12(3):e0173039. doi: 10.1371/journal.pone.0173039 (PMC5336250; doi:10.1371/journal.pone.0173039)
Supplement: S1 Table — (DOC) [file pone.0173039.s002.doc]

**S1 Table. List of accessions of the Colombian Central Collection of *S. tuberosum* Andigenum** group and information of sample collection sites.

| **N°** | **Parcel** | **Number of Register CCC** | **Country** | **Department** | **Town** | **Latitude*** | **Longitude*** | **Altitude (MASL)** | **Population by Structure** | **Subpopulation by Structure** |
| --- | --- | --- | --- | --- | --- | --- | --- | --- | --- | --- |
| 1 | And_1 | 15061903 | COLOMBIA | BOYACA | SOTAQUIRA | 5.77 | -73.25 | 2689.64 | Andigena | Andigena_4 |
| 2 | And_2 | 15060006 | COLOMBIA | BOYACA | PUEBLO VIEJO | 5.52 | -73.63 | 2335.77 | Andigena | Andigena_2 |
| 3 | And_3 | 15060013 | COLOMBIA | BOYACA | FIRAVITOBA | 5.65 | -73.00 | 2497.64 | Andigena | Andigena_3 |
| 4 | And_4 | 15061904 | COLOMBIA | BOYACA | FIRAVITOBA | 5.65 | -73.00 | 2497.64 | Phureja | Phureja_1 |
| 5 | And_5 | 15060016 | COLOMBIA | BOYACA | FIRAVITOBA | 5.65 | -73.00 | 2497.64 | Andigena | Andigena_2 |
| 6 | And_6 | 15060021 | COLOMBIA | CUNDINAMARCA | SUBACHOQUE | 4.93 | -74.18 | 2695.21 | Andigena | Andigena_2 |
| 7 | And_7 | 15060025 | COLOMBIA | CUNDINAMARCA | CAJICA | 4.92 | -74.03 | 2558.00 | Andigena | Andigena_5 |
| 8 | And_8 | 15060027 | COLOMBIA | CUNDINAMARCA | SUBACHOQUE | 4.93 | -74.18 | 2695.21 | Andigena | Andigena_3 |
| 9 | And_9 | 15062333 | COLOMBIA | BOYACA | TUNJA | 5.53 | -73.37 | 2796.80 | Andigena | Andigena_3 |
| 10 | And_10 | 15060039 | COLOMBIA | NARIÑO | PASTO | 1.21 | -77.28 | 2541.46 | Andigena | Andigena_4 |
| 11 | And_11 | 15062036 | COLOMBIA | NARIÑO | PASTO | 1.21 | -77.28 | 2541.46 | Andigena | Andigena_5 |
| 12 | And_12 | 15060042 | COLOMBIA | NARIÑO | PASTO | 1.21 | -77.28 | 2541.46 | Andigena | Andigena_5 |
| 13 | And_13 | 15062038 | COLOMBIA | NARIÑO | PASTO | 1.21 | -77.28 | 2541.46 | Andigena | Andigena_3 |
| 14 | And_14 | 15060050 | COLOMBIA | NARIÑO | PASTO | 1.21 | -77.28 | 2541.46 | Andigena | Andigena_1 |
| 15 | And_15 | 15060056 | COLOMBIA | NARIÑO | TUQUERRES | 1.09 | -77.62 | 3068.29 | Andigena | Andigena_5 |
| 16 | And_16 | 15061905 | COLOMBIA | NARIÑO | TUQUERRES | 1.09 | -77.62 | 3068.29 | Andigena | Andigena_4 |
| 17 | And_17 | 15060063 | COLOMBIA | NARIÑO | TUQUERRES | 1.09 | -77.62 | 3068.29 | Andigena | Andigena_5 |
| 18 | And_18 | 15060070 | COLOMBIA | NARIÑO | PUPIALES | 0.90 | -77.62 | 2931.83 | Andigena | Andigena_5 |
| 19 | And_19 | 15062039 | COLOMBIA | NARIÑO | IPIALES | 0.82 | -77.64 | 2868.92 | Andigena | Andigena_4 |
| 20 | And_20 | 15060072 | COLOMBIA | NARIÑO | CUMBAL | 0.90 | -77.78 | 3090.99 | Andigena | Andigena_4 |
| 21 | And_21 | 15061906 | COLOMBIA | NARIÑO | CUMBAL | 0.90 | -77.78 | 3090.99 | Andigena | Andigena_3 |
| 22 | And_22 | 15061907 | ECUADOR | CARCHI | SAN GABRIEL | 0.59 | -77.83 | 2815.87 | Andigena | Andigena_4 |
| 23 | And_23 | 15060081 | ECUADOR | CARCHI | SAN GABRIEL | 0.59 | -77.83 | 2815.87 | Andigena | Andigena_5 |
| 24 | And_24 | 15062331 | ECUADOR | CARCHI | SAN GABRIEL | 0.59 | -77.83 | 2815.87 | Andigena | Andigena_1 |
| 25 | And_25 | 15060082 | ECUADOR | CARCHI | SAN GABRIEL | 0.59 | -77.83 | 2815.87 | Andigena | Andigena_5 |
| 26 | And_26 | 15062332 | ECUADOR | IMBABURA | IBARRA | 0.36 | -78.13 | 2232.03 | Andigena | Andigena_1 |
| 27 | And_27 | 15061908 | ECUADOR | IMBABURA | IBARRA | 0.36 | -78.13 | 2232.03 | Andigena | Andigena_5 |
| 28 | And_28 | 15060085 | ECUADOR | IMBABURA | IBARRA | 0.36 | -78.13 | 2232.03 | Andigena | Andigena_5 |
| 29 | And_29 | 15061909 | ECUADOR | IMBABURA | IBARRA | 0.36 | -78.13 | 2232.03 | Andigena | Andigena_5 |
| 30 | And_30 | 15060087 | ECUADOR | IMBABURA | IBARRA | 0.36 | -78.13 | 2232.03 | Andigena | Andigena_5 |
| 31 | And_31 | 15060090 | COLOMBIA | VALLE DEL CAUCA | PALMIRA | 3.58 | -76.25 | 1060.55 | Andigena | Andigena_1 |
| 32 | And_32 | 15060097 | COLOMBIA | CAUCA | SILVIA | 2.61 | -76.34 | 3091.81 | Andigena | Andigena_4 |
| 33 | And_34 | 15060101 | COLOMBIA | CAUCA | SILVIA | 2.61 | -76.34 | 3091.81 | Andigena | Andigena_5 |
| 34 | And_35 | 15060102 | COLOMBIA | CAUCA | SAN PEDRO | 1.53 | -76.51 | 1637.08 | Andigena | Andigena_5 |
| 35 | And_36 | 15061910 | COLOMBIA | TOLIMA | MURILLO | 4.09 | -75.15 | 410.93 | Andigena | Andigena_4 |
| 36 | And_37 | 15060110 | COLOMBIA | TOLIMA | MURILLO | 4.09 | -75.15 | 410.93 | Andigena | Andigena_5 |
| 37 | And_38 | 15060112 | COLOMBIA | CALDAS | MANIZALES | 5.07 | -75.52 | 2138.07 | Andigena | Andigena_5 |
| 38 | And_39 | 15060114 | COLOMBIA | CALDAS | MANIZALES | 5.07 | -75.52 | 2138.07 | Andigena | Andigena_4 |
| 39 | And_40 | 15060117 | Unknown | Unknown | Unknown | Unknown | Unknown | Unknown | Andigena | Andigena_5 |
| 40 | And_41 | 15060120 | COLOMBIA | NORTE DE SANTANDER | SILOS | 7.20 | -72.77 | 2472.37 | Andigena | Andigena_5 |
| 41 | And_42 | 15060121 | COLOMBIA | SANTANDER | BUCARAMANGA | 7.11 | -73.12 | 948.10 | Andigena | Andigena_3 |
| 42 | And_43 | 15060122 | COLOMBIA | SANTANDER | BUCARAMANGA | 7.11 | -73.12 | 948.10 | Andigena | Andigena_1 |
| 43 | And_44 | 15061911 | VENEZUELA | MERIDA | Unknown | 8.60 | -71.15 | 1611.75 | Andigena | Andigena_4 |
| 44 | And_45 | 15060133 | ESTADOS UNIDOS | CAMBRIDGE | Unknown | 42.37 | -71.11 | 4.74 | Andigena | Andigena_5 |
| 45 | And_46 | 15060138 | COLOMBIA | MAGDALENA | SANTA MARTA | 10.83 | -73.67 | 5245.32 | Andigena | Andigena_5 |
| 46 | And_47 | 15062046 | COLOMBIA | NARIÑO | PASTO | 1.21 | -77.28 | 2541.46 | Andigena | Andigena_5 |
| 47 | And_48 | 15061912 | COLOMBIA | NARIÑO | PASTO | 1.21 | -77.28 | 2541.46 | Andigena | Andigena_5 |
| 48 | And_49 | 15060155 | Unknown | Unknown | Unknown | Unknown | Unknown | Unknown | Andigena | Andigena_2 |
| 49 | And_50 | 15061913 | Unknown | Unknown | Unknown | Unknown | Unknown | Unknown | Andigena | Andigena_5 |
| 50 | And_51 | 15061914 | ESTADOS UNIDOS | Unknown | Unknown | 37.43 | -78.66 | 213.64 | Andigena | Andigena_3 |
| 51 | And_52 | 15060160 | PERÚ | Unknown | Unknown | -9.99 | -75.96 | 3654.76 | Andigena | Andigena_1 |
| 52 | And_53 | 15061915 | PERÚ | Unknown | Unknown | -9.99 | -75.96 | 3654.76 | Andigena | Andigena_4 |
| 53 | And_54 | 15062334 | PERÚ | HUANCAYO | Unknown | -12.07 | -75.21 | 3255.33 | Andigena | Andigena_1 |
| 54 | And_55 | 15062050 | PERÚ | LIMA | Unknown | -12.05 | -77.04 | 138.30 | Andigena | Andigena_2 |
| 55 | And_56 | 15060187 | BOLIVIA | Unknown | Unknown | -16.85 | -65.74 | 1225.58 | Andigena | Andigena_2 |
| 56 | And_57 | 15061916 | BOLIVIA | Unknown | Unknown | -16.85 | -65.74 | 1225.58 | Andigena | Andigena_5 |
| 57 | And_58 | 15062335 | BOLIVIA | Unknown | Unknown | -16.85 | -65.74 | 1225.58 | Andigena | Andigena_3 |
| 58 | And_59 | 15060188 | COLOMBIA | CUNDIMANARCA | UNE | 4.40 | -74.02 | 2370.46 | Andigena | Andigena_5 |
| 59 | And_60 | 15060190 | COLOMBIA | CUNDINAMARCA | UNE | 4.33 | -74.08 | 3606.06 | Andigena | Andigena_5 |
| 60 | And_61 | 15060191 | COLOMBIA | CUNDINAMARCA | UNE | 4.33 | -74.08 | 3606.06 | Andigena | Andigena_5 |
| 61 | And_62 | 15060214 | COLOMBIA | NARIÑO | CUMBAL | 0.90 | -77.78 | 3090.99 | Andigena | Andigena_5 |
| 62 | And_63 | 15060215 | ECUADOR | IBARRA | Unknown | 0.36 | -78.13 | 2232.03 | Andigena | Andigena_4 |
| 63 | And_65 | 15060313 | PERÚ | RENACIMIENTO | LA MOLINA | -12.08 | -76.93 | 267.94 | Andigena | Andigena_4 |
| 64 | And_66 | 15060320 | PERÚ | Unknown | Unknown | -9.99 | -75.96 | 3654.76 | Andigena | Andigena_3 |
| 65 | And_67 | 15060345 | PERÚ | Unknown | Unknown | -9.99 | -75.96 | 3654.76 | Andigena | Andigena_3 |
| 66 | And_68 | 15062337 | PERÚ | Unknown | Unknown | -9.99 | -75.96 | 3654.76 | Andigena | Andigena_3 |
| 67 | And_69 | 15062051 | PERÚ | Unknown | Unknown | -9.99 | -75.96 | 3654.76 | Andigena | Andigena_3 |
| 68 | And_70 | 15062052 | PERÚ | Unknown | Unknown | -9.99 | -75.96 | 3654.76 | Andigena | Andigena_3 |
| 69 | And_71 | 15061917 | PERÚ | Unknown | Unknown | -9.99 | -75.96 | 3654.76 | Andigena | Andigena_3 |
| 70 | And_72 | 15060383 | BOLIVIA | LA PAZ | Unknown | -16.50 | -68.15 | 3781.17 | Andigena | Andigena_4 |
| 71 | And_73 | 15062338 | BOLIVIA | LA PAZ | Unknown | -16.50 | -68.15 | 3781.17 | Andigena | Andigena_3 |
| 72 | And_74 | 15062339 | BOLIVIA | ORURO | Unknown | -17.97 | -67.12 | 3738.08 | Andigena | Andigena_5 |
| 73 | And_75 | 15062340 | BOLIVIA | POTOSI | Unknown | -19.57 | -65.76 | 3887.65 | Andigena | Andigena_3 |
| 74 | And_76 | 15060420 | BOLIVIA | POTOSI | SAAVEDRA | -17.23 | -63.22 | 278.06 | Andigena | Andigena_3 |
| 75 | And_77 | 15062053 | PERÚ | PUNO | Unknown | -15.23 | -70.05 | 3831.16 | Andigena | Andigena_4 |
| 76 | And_78 | 15062363 | Unknown | Unknown | Unknown | Unknown | Unknown | Unknown | Andigena | Andigena_4 |
| 77 | And_80 | 15060536 | PERÚ | LA LIBERTAD | SANTIAGO DE CHUCO | -8.22 | -78.20 | 3755.42 | Andigena | Andigena_4 |
| 78 | And_81 | 15060543 | PERÚ | CAJAMARCA | Unknown | -6.45 | -78.84 | 2390.93 | Andigena | Andigena_5 |
| 79 | And_82 | 15060570 | PERÚ | LA LIBERTAD | SANTIAGO DE CHUCO | -8.22 | -78.20 | 3755.42 | Andigena | Andigena_5 |
| 80 | And_83 | 15060667 | PERÚ | LA LIBERTAD | AGALLPAMPA | -7.98 | -78.54 | 3124.95 | Andigena | Andigena_4 |
| 81 | And_84 | 15060797 | PERÚ | JUNIN | TARMA | -11.42 | -75.69 | 3056.41 | Andigena | Andigena_5 |
| 82 | And_85 | 15061918 | PERÚ | Unknown | Unknown | -9.99 | -75.96 | 3654.76 | Andigena | Andigena_1 |
| 83 | And_86 | 15060846 | PERÚ | Unknown | Unknown | -9.99 | -75.96 | 3654.76 | Andigena | Andigena_1 |
| 84 | And_87 | 15060907 | PERÚ | JUNIN | JAGUA | -11.33 | -75.34 | 1713.57 | Andigena | Andigena_4 |
| 85 | And_88 | 15060909 | PERÚ | JUNIN | JAGUA | -11.33 | -75.34 | 1713.57 | Andigena | Andigena_4 |
| 86 | And_89 | 15060933 | PERÚ | JUNIN | JAGUA | -11.33 | -75.34 | 1713.57 | Andigena | Andigena_5 |
| 87 | And_90 | 15060964 | PERÚ | JUNIN | JAGUA | -11.33 | -75.34 | 1713.57 | Andigena | Andigena_5 |
| 88 | And_91 | 15061007 | PERÚ | JUNIN | JAGUA | -11.33 | -75.34 | 1713.57 | Andigena | Andigena_5 |
| 89 | And_92 | 15061011 | PERÚ | JUNIN | JAGUA | -11.33 | -75.34 | 1713.57 | Andigena | Andigena_5 |
| 90 | And_93 | 15061122 | BOLIVIA | POTOSI | POTOSI | -19.57 | -65.76 | 3887.65 | Andigena | Andigena_1 |
| 91 | And_94 | 15061140 | PERÚ | JUNIN | TARMA | -11.42 | -75.69 | 3056.41 | Andigena | Andigena_4 |
| 92 | And_95 | 15061204 | BOLIVIA | TARIJA | Unknown | -21.53 | -64.73 | 1874.63 | Andigena | Andigena_4 |
| 93 | And_96 | 15061213 | PERÚ | PURINA | ANDAHUAYLAS | -13.66 | -73.38 | 2897.25 | Andigena | Andigena_4 |
| 94 | And_97 | 15061215 | PERÚ | ABANCAY | Unknown | -13.63 | -72.88 | 2407.24 | Andigena | Andigena_4 |
| 95 | And_98 | 15061216 | PERÚ | CURAHUASIJ | PURINAC | -14.05 | -73.09 | 3701.76 | Andigena | Andigena_5 |
| 96 | And_99 | 15061225 | PERÚ | HUANCA VELICA | TAYA | -16.02 | -71.93 | 3358.87 | Andigena | Andigena_4 |
| 97 | And_100 | 15061226 | PERÚ | HUANCA VELICA | TAYA | -16.02 | -71.93 | 3358.87 | Andigena | Andigena_5 |
| 98 | And_101 | 15061228 | PERÚ | AYACUCHO | QUINUA | -13.05 | -74.14 | 3223.16 | Andigena | Andigena_4 |
| 99 | And_104 | 15061237 | PERÚ | HUANCAVELICA | Unknown | -12.79 | -74.98 | 3679.97 | Andigena | Andigena_5 |
| 100 | And_107 | 15061922 | PERU | CUSCO | CUSCO | -13.53 | -71.96 | 3200.00 | Andigena | Andigena_5 |
| 101 | And_108 | 15061250 | COLOMBIA | NARIÑO | PASTO | 1.21 | -77.28 | 2541.46 | Andigena | Andigena_5 |
| 102 | And_109 | 15061923 | Unknown | Unknown | Unknown | Unknown | Unknown | Unknown | Andigena | Andigena_4 |
| 103 | And_110 | PALA ROJA | BOLIVIA | LA PAZ | LA PAZ | -16.50 | -68.14 | 3600.00 | Andigena | Andigena_1 |
| 104 | And_112 | 15062343 | COLOMBIA | NARIÑO | PASTO | 1.21 | -77.28 | 2541.46 | Andigena | Andigena_5 |
| 105 | And_113 | 15061256 | COLOMBIA | NARIÑO | PASTO | 1.21 | -77.28 | 2541.46 | Andigena | Andigena_5 |
| 106 | And_114 | 15061257 | COLOMBIA | NARIÑO | PASTO | 1.21 | -77.28 | 2541.46 | Andigena | Andigena_5 |
| 107 | And_115 | 15062059 | COLOMBIA | NARIÑO | PASTO | 1.21 | -77.28 | 2541.46 | Andigena | Andigena_5 |
| 108 | And_116 | 15061258 | COLOMBIA | NARIÑO | IPIALES | 0.82 | -77.64 | 2868.92 | Andigena | Andigena_5 |
| 109 | And_117 | 15062060 | COLOMBIA | NARIÑO | IPIALES | 0.82 | -77.64 | 2868.92 | Andigena | Andigena_5 |
| 110 | And_118 | 15061924 | COLOMBIA | NARIÑO | TUQUERRES | 1.09 | -77.62 | 3068.29 | Andigena | Andigena_5 |
| 111 | And_119 | 15061259 | COLOMBIA | NARIÑO | TUQUERRES | 1.09 | -77.62 | 3068.29 | Andigena | Andigena_5 |
| 112 | And_120 | 15061925 | COLOMBIA | NARIÑO | Unknown | 1.29 | -77.36 | 2356.38 | Andigena | Andigena_5 |
| 113 | And_121 | 15062063 | COLOMBIA | CUNDINAMARCA | VILLAPINZON | 5.22 | -73.60 | 2723.82 | Andigena | Andigena_5 |
| 114 | And_122 | 15061926 | COLOMBIA | CUNDINAMARCA | SESQUILE | 5.04 | -73.80 | 2570.70 | Andigena | Andigena_4 |
| 115 | And_123 | 15061927 | COLOMBIA | CUNDINAMARCA | SESQUILE | 5.04 | -73.80 | 2570.70 | Andigena | Andigena_5 |
| 116 | And_124 | 15062344 | COLOMBIA | CUNDINAMARCA | SESQUILE | 5.04 | -73.80 | 2570.70 | Andigena | Andigena_2 |
| 117 | And_125 | 15061928 | COLOMBIA | CUNDINAMARCA | SESQUILE | 5.04 | -73.80 | 2570.70 | Andigena | Andigena_2 |
| 118 | And_126 | 15062067 | COLOMBIA | CUNDINAMARCA | SESQUILE | 5.04 | -73.80 | 2570.70 | Andigena | Andigena_4 |
| 119 | And_127 | 15061929 | COLOMBIA | CUNDINAMARCA | MACHETÁ | 5.08 | -73.61 | 2100.27 | Andigena | Andigena_2 |
| 120 | And_127_A | 15061929_A | COLOMBIA | CUNDINAMARCA | MACHETA | 5.08 | -73.61 | 2103.56 | Andigena | Andigena_3 |
| 121 | And_128 | 15061930 | COLOMBIA | CUNDINAMARCA | MACHETÁ | 5.08 | -73.61 | 2100.27 | Andigena | Andigena_1 |
| 122 | And_129 | 15062069 | COLOMBIA | CUNDINAMARCA | MACHETÁ | 5.08 | -73.61 | 2100.27 | Andigena | Andigena_2 |
| 123 | And_130 | 15061931 | COLOMBIA | CUNDINAMARCA | CHOCONTA | 5.14 | -73.69 | 2652.31 | Andigena | Andigena_5 |
| 124 | And_131 | 15061260 | COLOMBIA | CUNDINAMARCA | CHOCONTA | 5.14 | -73.69 | 2652.31 | Andigena | Andigena_5 |
| 125 | And_132 | 15061261 | COLOMBIA | CUNDINAMARCA | CHOCONTA | 5.14 | -73.69 | 2652.31 | Andigena | Andigena_5 |
| 126 | And_133 | 15061262 | COLOMBIA | BOYACA | MONGUI | 5.69 | -72.84 | 3411.82 | Andigena | Andigena_5 |
| 127 | And_134 | 15062071 | COLOMBIA | BOYACA | MONGUI | 5.69 | -72.84 | 3411.82 | Andigena | Andigena_5 |
| 128 | And_135 | 15061263 | COLOMBIA | BOYACA | MONGUI | 5.69 | -72.84 | 3411.82 | Andigena | Andigena_5 |
| 129 | And_136 | 15061932 | COLOMBIA | BOYACA | MONGUI | 5.69 | -72.84 | 3411.82 | Andigena | Andigena_4 |
| 130 | And_137 | 15061264 | COLOMBIA | BOYACA | MONGUI | 5.69 | -72.84 | 3411.82 | Andigena | Andigena_5 |
| 131 | And_138 | 15061933 | COLOMBIA | BOYACA | MONGUI | 5.69 | -72.84 | 3411.82 | Andigena | Andigena_5 |
| 132 | And_139 | 15061265 | COLOMBIA | BOYACA | MONGUI | 5.69 | -72.84 | 3411.82 | Andigena | Andigena_3 |
| 133 | And_140 | 15061270 | COLOMBIA | BOYACA | MONGUI | 5.69 | -72.84 | 3411.82 | Andigena | Andigena_3 |
| 134 | And_141 | 15062074 | COLOMBIA | BOYACA | MONGUI | 5.69 | -72.84 | 3411.82 | Andigena | Andigena_3 |
| 135 | And_142 | 15061934 | COLOMBIA | BOYACA | MONGUI | 5.69 | -72.84 | 3411.82 | Andigena | Andigena_5 |
| 136 | And_143 | 15061935 | COLOMBIA | BOYACA | MONGUI | 5.69 | -72.84 | 3411.82 | Andigena | Andigena_4 |
| 137 | And_144 | 15061271 | COLOMBIA | BOYACA | MONGUI | 5.69 | -72.84 | 3411.82 | Andigena | Andigena_3 |
| 138 | And_146 | 15061273 | COLOMBIA | BOYACA | SOGAMOSO | 5.72 | -72.92 | 2584.94 | Andigena | Andigena_3 |
| 139 | And_147 | 15061274 | COLOMBIA | BOYACA | SOGAMOSO | 5.72 | -72.92 | 2584.94 | Andigena | Andigena_5 |
| 140 | And_148 | 15061937 | COLOMBIA | TOLIMA | CAJAMARCA | 4.42 | -75.50 | 3127.12 | Andigena | Andigena_3 |
| 141 | And_149 | 15061277 | COLOMBIA | QUINDIO | SALENTO | 4.63 | -75.57 | 1934.53 | Andigena | Andigena_4 |
| 142 | And_150 | 15062080 | COLOMBIA | QUINDIO | CALARCA | 4.53 | -75.64 | 1515.85 | Andigena | Andigena_3 |
| 143 | And_151 | 15062081 | COLOMBIA | QUINDIO | ARMENIA | 4.53 | -75.67 | 1490.56 | Andigena | Andigena_3 |
| 144 | And_152 | 15061279 | COLOMBIA | QUINDIO | SALENTO | 4.63 | -75.57 | 2520.00 | Andigena | Andigena_5 |
| 145 | And_154 | 15061281 | COLOMBIA | QUINDIO | CALARCA | 4.53 | -75.64 | 1515.85 | Andigena | Andigena_4 |
| 146 | And_155 | 15061939 | COLOMBIA | CALDAS | MANIZALES | 5.07 | -75.52 | 2138.07 | Andigena | Andigena_3 |
| 147 | And_156 | 15061282 | COLOMBIA | CALDAS | Unknown | 5.48 | -75.21 | 1573.36 | Andigena | Andigena_4 |
| 148 | And_157 | 15060910 | COLOMBIA | VALLE DEL CAUCA | CALI | 3.46 | -76.52 | 982.58 | Andigena | Andigena_4 |
| 149 | And_158 | 15062365 | COLOMBIA | QUINDIO | ARMENIA | 4.53 | -75.67 | 1490.56 | Andigena | Andigena_5 |
| 150 | And_160 | 15061283 | COLOMBIA | QUINDIO | ARMENIA | 4.53 | -75.70 | 1366.03 | Andigena | Andigena_5 |
| 151 | And_163 | 15062346 | COLOMBIA | QUINDIO | ARMENIA | 4.53 | -75.70 | 1366.03 | Andigena | Andigena_5 |
| 152 | And_164 | 15061286 | COLOMBIA | BOYACA | SOGAMOSO | 5.72 | -72.92 | 2584.94 | Andigena | Andigena_3 |
| 153 | And_166 | 15061288 | COLOMBIA | BOYACA | SOGAMOSO | 5.72 | -72.92 | 2584.94 | Andigena | Andigena_4 |
| 154 | And_167 | 15061289 | COLOMBIA | BOYACA | SOGAMOSO | 5.72 | -72.92 | 2584.94 | Andigena | Andigena_4 |
| 155 | And_168 | 15061290 | COLOMBIA | BOYACA | SOGAMOSO | 5.72 | -72.92 | 2584.94 | Andigena | Andigena_4 |
| 156 | And_169 | 15061291 | COLOMBIA | BOYACA | SOGAMOSO | 5.72 | -72.92 | 2584.94 | Andigena | Andigena_5 |
| 157 | And_171 | 15061293 | COLOMBIA | BOYACA | SOGAMOSO | 5.72 | -72.92 | 2584.94 | Andigena | Andigena_5 |
| 158 | And_172 | 15061295 | COLOMBIA | BOYACA | AQUITANIA | 5.52 | -72.88 | 3047.38 | Andigena | Andigena_2 |
| 159 | And_173 | 15061941 | COLOMBIA | BOYACA | AQUITANIA | 5.52 | -72.88 | 3047.38 | Andigena | Andigena_3 |
| 160 | And_174 | 15061296 | COLOMBIA | BOYACA | AQUITANIA | 5.52 | -72.88 | 3047.38 | Andigena | Andigena_5 |
| 161 | And_175 | 15061297 | COLOMBIA | BOYACA | AQUITANIA | 5.52 | -72.88 | 3047.38 | Andigena | Andigena_5 |
| 162 | And_176 | 15061942 | COLOMBIA | BOYACA | AQUITANIA | 5.52 | -72.88 | 3047.38 | Andigena | Andigena_5 |
| 163 | And_177 | 15061298 | COLOMBIA | BOYACA | AQUITANIA | 5.52 | -72.88 | 3047.38 | Andigena | Andigena_5 |
| 164 | And_178 | 15061299 | COLOMBIA | BOYACA | AQUITANIA | 5.52 | -72.88 | 3047.38 | Andigena | Andigena_4 |
| 165 | And_179 | 15061300 | COLOMBIA | BOYACA | AQUITANIA | 5.52 | -72.88 | 3047.38 | Andigena | Andigena_3 |
| 166 | And_180 | 15061301 | COLOMBIA | BOYACA | AQUITANIA | 5.52 | -72.88 | 3047.38 | Andigena | Andigena_5 |
| 167 | And_181 | 15061302 | COLOMBIA | BOYACA | AQUITANIA | 5.52 | -72.88 | 3047.38 | Andigena | Andigena_3 |
| 168 | And_182 | 15061303 | COLOMBIA | BOYACA | AQUITANIA | 5.52 | -72.88 | 3047.38 | Andigena | Andigena_4 |
| 169 | And_183 | 15061304 | COLOMBIA | BOYACA | AQUITANIA | 5.52 | -72.88 | 3040.99 | Phureja | Phureja_1 |
| 170 | And_184 | 15061305 | COLOMBIA | BOYACA | AQUITANIA | 5.52 | -72.88 | 3047.38 | Andigena | Andigena_5 |
| 171 | And_185 | 15061307 | COLOMBIA | BOYACA | AQUITANIA | 5.52 | -72.88 | 3047.38 | Andigena | Andigena_4 |
| 172 | And_186 | 15061308 | COLOMBIA | BOYACA | AQUITANIA | 5.52 | -72.88 | 3047.38 | Andigena | Andigena_3 |
| 173 | And_188 | 15061312 | COLOMBIA | BOYACA | AQUITANIA | 5.52 | -72.88 | 3047.38 | Andigena | Andigena_5 |
| 174 | And_189 | 15061313 | COLOMBIA | BOYACA | AQUITANIA | 5.52 | -72.88 | 3047.38 | Andigena | Andigena_5 |
| 175 | And_190 | 15061314 | COLOMBIA | BOYACA | AQUITANIA | 5.52 | -72.88 | 3047.38 | Andigena | Andigena_3 |
| 176 | And_191 | 15061944 | COLOMBIA | BOYACA | CERINZA | 5.95 | -72.95 | 2724.64 | Andigena | Andigena_3 |
| 177 | And_193 | 15061317 | COLOMBIA | BOYACA | BELEN | 5.98 | -72.88 | 2595.36 | Andigena | Andigena_5 |
| 178 | And_194 | 15061318 | COLOMBIA | BOYACA | SUSACON | 6.23 | -72.70 | 2375.65 | Andigena | Andigena_5 |
| 179 | And_195 | 15061319 | COLOMBIA | BOYACA | BOAVITA | 6.33 | -72.58 | 2218.32 | Andigena | Andigena_5 |
| 180 | And_196 | 15061320 | COLOMBIA | BOYACA | BOAVITA | 6.33 | -72.58 | 2218.32 | Andigena | Andigena_4 |
| 181 | And_198 | 15061323 | COLOMBIA | BOYACA | BOAVITA | 6.33 | -72.58 | 2218.32 | Andigena | Andigena_4 |
| 182 | And_199 | 15061324 | COLOMBIA | BOYACA | BOAVITA | 6.33 | -72.58 | 2218.32 | Andigena | Andigena_4 |
| 183 | And_200 | 15061327 | COLOMBIA | BOYACA | CHITA | 6.17 | -72.41 | 3587.50 | Andigena | Andigena_5 |
| 184 | And_201 | 15061329 | COLOMBIA | BOYACA | CHITA | 6.17 | -72.41 | 3587.50 | Andigena | Andigena_3 |
| 185 | And_202 | 15061330 | COLOMBIA | BOYACA | CHITA | 6.17 | -72.41 | 3587.50 | Andigena | Andigena_4 |
| 186 | And_203 | 15061331 | COLOMBIA | BOYACA | CHITA | 6.17 | -72.41 | 3587.50 | Andigena | Andigena_5 |
| 187 | And_204 | 15061333 | COLOMBIA | BOYACA | EL COCUY | 6.42 | -72.42 | 3479.20 | Andigena | Andigena_3 |
| 188 | And_205 | 15061334 | COLOMBIA | BOYACA | EL COCUY | 6.42 | -72.42 | 3479.20 | Andigena | Andigena_3 |
| 189 | And_206 | 15061335 | COLOMBIA | BOYACA | EL COCUY | 6.42 | -72.42 | 3479.20 | Andigena | Andigena_5 |
| 190 | And_207 | 15061336 | COLOMBIA | BOYACA | EL COCUY | 6.42 | -72.42 | 3479.20 | Andigena | Andigena_3 |
| 191 | And_208 | 15061337 | COLOMBIA | BOYACA | EL COCUY | 6.42 | -72.42 | 3479.20 | Andigena | Andigena_5 |
| 192 | And_209 | 15061338 | COLOMBIA | BOYACA | EL COCUY | 6.42 | -72.42 | 3479.20 | Andigena | Andigena_5 |
| 193 | And_210 | 15061340 | COLOMBIA | BOYACA | EL COCUY | 6.42 | -72.42 | 3479.20 | Andigena | Andigena_5 |
| 194 | And_211 | 15061342 | COLOMBIA | BOYACA | EL COCUY | 6.42 | -72.42 | 3479.20 | Andigena | Andigena_5 |
| 195 | And_212 | 15061344 | COLOMBIA | BOYACA | EL COCUY | 6.42 | -72.42 | 3479.20 | Andigena | Andigena_5 |
| 196 | And_213 | 15061345 | COLOMBIA | BOYACA | EL COCUY | 6.42 | -72.42 | 3479.20 | Andigena | Andigena_3 |
| 197 | And_214 | 15061945 | COLOMBIA | BOYACA | EL COCUY | 6.42 | -72.42 | 3479.20 | Andigena | Andigena_3 |
| 198 | And_215 | 15061346 | COLOMBIA | BOYACA | EL COCUY | 6.42 | -72.42 | 3479.20 | Andigena | Andigena_3 |
| 199 | And_216 | 15061347 | COLOMBIA | BOYACA | EL COCUY | 6.42 | -72.42 | 3479.20 | Andigena | Andigena_2 |
| 200 | And_217 | 15061348 | COLOMBIA | BOYACA | EL COCUY | 6.42 | -72.42 | 3479.20 | Andigena | Andigena_5 |
| 201 | And_218 | 15061349 | COLOMBIA | BOYACA | EL COCUY | 6.42 | -72.42 | 3479.20 | Andigena | Andigena_5 |
| 202 | And_219 | 15061350 | COLOMBIA | BOYACA | EL COCUY | 6.42 | -72.42 | 3479.20 | Andigena | Andigena_5 |
| 203 | And_220 | 15061352 | COLOMBIA | BOYACA | GUICAN | 6.47 | -72.42 | 2768.77 | Andigena | Andigena_3 |
| 204 | And_221 | 15061353 | COLOMBIA | BOYACA | GUICAN | 6.47 | -72.42 | 2768.77 | Andigena | Andigena_5 |
| 205 | And_222 | 15061946 | COLOMBIA | BOYACA | GUICAN | 6.47 | -72.42 | 2768.77 | Andigena | Andigena_2 |
| 206 | And_225 | 15061355 | COLOMBIA | BOYACA | GUICAN | 6.47 | -72.42 | 2768.77 | Andigena | Andigena_5 |
| 207 | And_226 | 15062092 | COLOMBIA | BOYACA | Unknown | 5.45 | -73.35 | 2379.10 | Andigena | Andigena_5 |
| 208 | And_227 | 15061356 | COLOMBIA | BOYACA | GUICAN | 6.47 | -72.42 | 2768.77 | Andigena | Andigena_1 |
| 209 | And_228 | 15061357 | COLOMBIA | BOYACA | GUICAN | 6.47 | -72.42 | 2768.77 | Andigena | Andigena_3 |
| 210 | And_229 | 15061358 | COLOMBIA | BOYACA | GUICAN | 6.47 | -72.42 | 2768.77 | Andigena | Andigena_5 |
| 211 | And_232 | 15061360 | COLOMBIA | BOYACA | CHISCAS | 6.72 | -72.38 | 3431.30 | Andigena | Andigena_5 |
| 212 | And_233 | 15061361 | COLOMBIA | BOYACA | CHISCAS | 6.72 | -72.38 | 3431.30 | Andigena | Andigena_5 |
| 213 | And_234 | 15062094 | COLOMBIA | NORTE DE SANTANDER | PAMPLONA | 7.39 | -72.66 | 2431.17 | Andigena | Andigena_5 |
| 214 | And_235 | 15061364 | COLOMBIA | NORTE DE SANTANDER | SILOS | 7.20 | -72.77 | 2472.37 | Andigena | Andigena_5 |
| 215 | And_236 | 15061365 | COLOMBIA | NORTE DE SANTANDER | SILOS | 7.20 | -72.77 | 2472.37 | Andigena | Andigena_4 |
| 216 | And_237 | 15061367 | COLOMBIA | NORTE DE SANTANDER | SILOS | 7.20 | -72.77 | 2472.37 | Andigena | Andigena_4 |
| 217 | And_238 | 15061948 | COLOMBIA | NORTE DE SANTANDER | SILOS | 7.20 | -72.77 | 2472.37 | Andigena | Andigena_5 |
| 218 | And_239 | 15061368 | COLOMBIA | NORTE DE SANTANDER | SILOS | 7.20 | -72.77 | 2472.37 | Andigena | Andigena_3 |
| 219 | And_240 | 15062347 | COLOMBIA | NORTE DE SANTANDER | SILOS | 7.20 | -72.77 | 2472.37 | Andigena | Andigena_3 |
| 220 | And_241 | 15061369 | COLOMBIA | SANTANDER | MALAGA | 6.71 | -72.73 | 2212.68 | Andigena | Andigena_3 |
| 221 | And_242 | 15062097 | COLOMBIA | SANTANDER | MALAGA | 6.71 | -72.73 | 2212.68 | Andigena | Andigena_5 |
| 222 | And_243 | 15061370 | COLOMBIA | SANTANDER | MALAGA | 6.71 | -72.73 | 2212.68 | Andigena | Andigena_3 |
| 223 | And_244 | 15062348 | COLOMBIA | SANTANDER | MALAGA | 6.71 | -72.73 | 2212.68 | Andigena | Andigena_3 |
| 224 | And_245 | 15062349 | COLOMBIA | SANTANDER | MALAGA | 6.71 | -72.73 | 2212.68 | Andigena | Andigena_3 |
| 225 | And_246 | 15062350 | COLOMBIA | SANTANDER | MALAGA | 6.71 | -72.73 | 2212.68 | Andigena | Andigena_3 |
| 226 | And_247 | 15061949 | COLOMBIA | SANTANDER | MALAGA | 6.71 | -72.73 | 2212.68 | Andigena | Andigena_3 |
| 227 | And_248 | 15062351 | COLOMBIA | SANTANDER | MALAGA | 6.71 | -72.73 | 2212.68 | Andigena | Andigena_2 |
| 228 | And_249 | 15062098 | COLOMBIA | SANTANDER | MALAGA | 6.71 | -72.73 | 2212.68 | Andigena | Andigena_3 |
| 229 | And_250 | 15061376 | COLOMBIA | SANTANDER | GUACA | 6.88 | -72.87 | 2762.49 | Andigena | Andigena_3 |
| 230 | And_251 | 15061377 | COLOMBIA | SANTANDER | GUACA | 6.88 | -72.87 | 2762.49 | Andigena | Andigena_5 |
| 231 | And_252 | 15061378 | COLOMBIA | SANTANDER | GUACA | 6.88 | -72.87 | 2762.49 | Andigena | Andigena_5 |
| 232 | And_253 | 15061380 | COLOMBIA | SANTANDER | GUACA | 6.88 | -72.87 | 2762.49 | Andigena | Andigena_2 |
| 233 | And_254 | 15061382 | COLOMBIA | NORTE DE SANTANDER | CHITAGA | 7.13 | -72.67 | 2293.73 | Andigena | Andigena_4 |
| 234 | And_255 | 15061383 | COLOMBIA | NORTE DE SANTANDER | HERRAN | 7.52 | -72.48 | 1916.70 | Andigena | Andigena_5 |
| 235 | And_256 | 15061384 | COLOMBIA | NORTE DE SANTANDER | MITISCUA | 7.28 | -72.77 | 3226.31 | Andigena | Andigena_5 |
| 236 | And_257 | 15061385 | COLOMBIA | NORTE DE SANTANDER | MITISCUA | 7.28 | -72.77 | 3226.31 | Andigena | Andigena_5 |
| 237 | And_258 | 15061386 | COLOMBIA | NORTE DE SANTANDER | MITISCUA | 7.28 | -72.77 | 3226.31 | Andigena | Andigena_3 |
| 238 | And_259 | 15061388 | COLOMBIA | NORTE DE SANTANDER | MITISCUA | 7.28 | -72.77 | 3226.31 | Andigena | Andigena_5 |
| 239 | And_260 | 15061389 | COLOMBIA | NORTE DE SANTANDER | MITISCUA | 7.28 | -72.77 | 3226.31 | Andigena | Andigena_3 |
| 240 | And_261 | 15061390 | COLOMBIA | NORTE DE SANTANDER | MITISCUA | 7.28 | -72.77 | 3226.31 | Andigena | Andigena_3 |
| 241 | And_262 | 15061391 | COLOMBIA | NORTE DE SANTANDER | MITISCUA | 7.28 | -72.77 | 3226.31 | Andigena | Andigena_3 |
| 242 | And_263 | 15061392 | COLOMBIA | NORTE DE SANTANDER | SILOS | 7.20 | -72.77 | 2472.37 | Andigena | Andigena_5 |
| 243 | And_265 | 15061394 | COLOMBIA | NORTE DE SANTANDER | SILOS | 7.20 | -72.77 | 2472.37 | Andigena | Andigena_5 |
| 244 | And_266 | 15061396 | COLOMBIA | SANTANDER | BUCARAMANGA | 7.11 | -73.12 | 948.10 | Andigena | Andigena_5 |
| 245 | And_267 | 15061397 | COLOMBIA | SANTANDER | BUCARAMANGA | 7.11 | -73.12 | 948.10 | Andigena | Andigena_5 |
| 246 | And_268 | 15062353 | COLOMBIA | BOYACA | COMBITA | 5.63 | -73.32 | 2746.94 | Andigena | Andigena_3 |
| 247 | And_271 | 15062099 | COLOMBIA | BOYACA | COMBITA | 5.63 | -73.32 | 2746.94 | Andigena | Andigena_5 |
| 248 | And_272 | 15062100 | COLOMBIA | BOYACA | COMBITA | 5.63 | -73.32 | 2746.94 | Andigena | Andigena_4 |
| 249 | And_273 | 15062101 | COLOMBIA | BOYACA | COMBITA | 5.63 | -73.32 | 2746.94 | Andigena | Andigena_3 |
| 250 | And_274 | 15062102 | COLOMBIA | BOYACA | COMBITA | 5.63 | -73.32 | 2746.94 | Andigena | Andigena_4 |
| 251 | And_275 | 15061404 | COLOMBIA | BOYACA | COMBITA | 5.63 | -73.32 | 2746.94 | Andigena | Andigena_3 |
| 252 | And_276 | 15061405 | COLOMBIA | BOYACA | TUNJA | 5.53 | -73.37 | 2796.80 | Andigena | Andigena_4 |
| 253 | And_277 | 15061406 | COLOMBIA | BOYACA | TUNJA | 5.53 | -73.37 | 2796.80 | Andigena | Andigena_5 |
| 254 | And_278 | 15061410 | COLOMBIA | NARIÑO | EL ENGAÑO | 1.17 | -77.17 | 2910.67 | Andigena | Andigena_4 |
| 255 | And_279 | 15061411 | COLOMBIA | NARIÑO | EL ENGAÑO | 1.17 | -77.17 | 2910.67 | Andigena | Andigena_5 |
| 256 | And_281 | 15062103 | COLOMBIA | NARIÑO | PASTO | 1.21 | -77.28 | 2541.46 | Andigena | Andigena_3 |
| 257 | And_282 | 15061414 | COLOMBIA | NARIÑO | Unknown | 1.29 | -77.36 | 2356.38 | Andigena | Andigena_3 |
| 258 | And_283 | 15062104 | COLOMBIA | NARIÑO | PASTO | 1.21 | -77.28 | 2541.46 | Andigena | Andigena_3 |
| 259 | And_284 | 15062105 | COLOMBIA | NARIÑO | PASTO | 1.21 | -77.28 | 2541.46 | Andigena | Andigena_5 |
| 260 | And_285 | 15061415 | COLOMBIA | NARIÑO | Unknown | 1.29 | -77.36 | 2356.38 | Andigena | Andigena_2 |
| 261 | And_286 | 15062106 | COLOMBIA | NARIÑO | PASTO | 1.21 | -77.28 | 2541.46 | Andigena | Andigena_3 |
| 262 | And_288 | 15061417 | COLOMBIA | NARIÑO | EL ENGAÑO | 1.17 | -77.17 | 2910.67 | Andigena | Andigena_3 |
| 263 | And_289 | 15061418 | COLOMBIA | NARIÑO | PASTO | 1.21 | -77.28 | 2541.46 | Andigena | Andigena_3 |
| 264 | And_290 | 15061419 | COLOMBIA | NARIÑO | PASTO | 1.21 | -77.28 | 2541.46 | Andigena | Andigena_5 |
| 265 | And_291 | 15061420 | COLOMBIA | NARIÑO | PASTO | 1.21 | -77.28 | 2541.46 | Andigena | Andigena_4 |
| 266 | And_292 | 15061421 | COLOMBIA | NARIÑO | Unknown | 1.29 | -77.36 | 2356.38 | Andigena | Andigena_5 |
| 267 | And_293 | 15062366 | COLOMBIA | NARIÑO | PASTO | 1.22 | -77.27 | 3030.00 | Andigena | Andigena_5 |
| 268 | And_294 | 15062107 | COLOMBIA | NARIÑO | PASTO | 1.21 | -77.28 | 2541.46 | Andigena | Andigena_5 |
| 269 | And_295 | 15062108 | COLOMBIA | NARIÑO | PASTO | 1.21 | -77.28 | 2541.46 | Andigena | Andigena_3 |
| 270 | And_296 | 15061422 | COLOMBIA | NARIÑO | PASTO | 1.21 | -77.28 | 2541.46 | Andigena | Andigena_5 |
| 271 | And_297 | 15061423 | COLOMBIA | NARIÑO | PASTO | 1.21 | -77.28 | 2541.46 | Andigena | Andigena_5 |
| 272 | And_298 | 15061424 | COLOMBIA | NARIÑO | PASTO | 1.21 | -77.28 | 2541.46 | Andigena | Andigena_5 |
| 273 | And_299 | 15061425 | COLOMBIA | NARIÑO | PASTO | 1.21 | -77.28 | 2541.46 | Andigena | Andigena_3 |
| 274 | And_300 | 15062109 | COLOMBIA | NARIÑO | PASTO | 1.21 | -77.28 | 2541.46 | Andigena | Andigena_3 |
| 275 | And_302 | 15062111 | COLOMBIA | NARIÑO | PASTO | 1.21 | -77.28 | 2541.46 | Andigena | Andigena_5 |
| 276 | And_303 | 15062112 | COLOMBIA | NARIÑO | PASTO | 1.21 | -77.28 | 2541.46 | Andigena | Andigena_5 |
| 277 | And_304 | 15061428 | COLOMBIA | NARIÑO | PASTO | 1.21 | -77.28 | 2541.46 | Andigena | Andigena_3 |
| 278 | And_305 | 15062354 | COLOMBIA | CUNDINAMARCA | SOACHA | 4.58 | -74.22 | 2558.22 | Andigena | Andigena_3 |
| 279 | And_306 | 15061951 | COLOMBIA | NARIÑO | PASTO | 1.21 | -77.28 | 2541.46 | Andigena | Andigena_3 |
| 280 | And_307 | 15061430 | COLOMBIA | NARIÑO | PASTO | 1.21 | -77.28 | 2541.46 | Andigena | Andigena_3 |
| 281 | And_308 | 15061434 | COLOMBIA | NARIÑO | IPIALES | 0.82 | -77.64 | 2868.92 | Andigena | Andigena_2 |
| 282 | And_309 | 15061434 | COLOMBIA | NARIÑO | IPIALES | 0.83 | -77.64 | 2901.73 | Andigena | Andigena_5 |
| 283 | And_310 | 15061435 | COLOMBIA | NARIÑO | IPIALES | 0.82 | -77.64 | 2868.92 | Andigena | Andigena_3 |
| 284 | And_311 | 15061436 | COLOMBIA | NARIÑO | IPIALES | 0.82 | -77.64 | 2868.92 | Andigena | Andigena_4 |
| 285 | And_312 | 15062114 | COLOMBIA | NARIÑO | IPIALES | 0.82 | -77.64 | 2868.92 | Andigena | Andigena_3 |
| 286 | And_313 | 15061952 | COLOMBIA | NARIÑO | PASTO | 1.21 | -77.28 | 2541.46 | Andigena | Andigena_5 |
| 287 | And_314 | 15062115 | COLOMBIA | NARIÑO | IPIALES | 0.82 | -77.64 | 2868.92 | Andigena | Andigena_3 |
| 288 | And_315 | 15061437 | COLOMBIA | NARIÑO | PASTO | 1.21 | -77.28 | 2541.46 | Andigena | Andigena_2 |
| 289 | And_316 | 15061438 | COLOMBIA | NARIÑO | PASTO | 1.21 | -77.28 | 2541.46 | Andigena | Andigena_3 |
| 290 | And_317 | 15061439 | COLOMBIA | NARIÑO | PASTO | 1.21 | -77.28 | 2541.46 | Andigena | Andigena_2 |
| 291 | And_318 | 15061441 | COLOMBIA | NARIÑO | PASTO | 1.21 | -77.28 | 2541.46 | Andigena | Andigena_3 |
| 292 | And_319 | 15061442 | COLOMBIA | NARIÑO | PASTO | 1.21 | -77.28 | 2541.46 | Andigena | Andigena_5 |
| 293 | And_320 | 15061443 | COLOMBIA | NARIÑO | PASTO | 1.21 | -77.28 | 2541.46 | Andigena | Andigena_4 |
| 294 | And_321 | 15062367 | COLOMBIA | NARIÑO | PASTO | 1.22 | -77.27 | 3030.00 | Andigena | Andigena_3 |
| 295 | And_322 | 15061444 | COLOMBIA | NARIÑO | IPIALES | 0.82 | -77.64 | 2868.92 | Andigena | Andigena_5 |
| 296 | And_323 | 15061445 | COLOMBIA | NARIÑO | IPIALES | 0.82 | -77.64 | 2868.92 | Andigena | Andigena_3 |
| 297 | And_324 | 15061953 | COLOMBIA | NARIÑO | IPIALES | 0.82 | -77.64 | 2868.92 | Andigena | Andigena_5 |
| 298 | And_325 | 15061447 | COLOMBIA | NARIÑO | IPIALES | 0.82 | -77.64 | 2868.92 | Andigena | Andigena_5 |
| 299 | And_326 | 15061449 | COLOMBIA | NARIÑO | IPIALES | 0.82 | -77.64 | 2868.92 | Andigena | Andigena_2 |
| 300 | And_327 | 15061453 | COLOMBIA | NARIÑO | IPIALES | 0.82 | -77.64 | 2868.92 | Andigena | Andigena_3 |
| 301 | And_328 | 15061454 | COLOMBIA | NARIÑO | IPIALES | 0.82 | -77.64 | 2868.92 | Andigena | Andigena_2 |
| 302 | And_329 | 15062117 | COLOMBIA | NARIÑO | IPIALES | 0.82 | -77.64 | 2868.92 | Andigena | Andigena_3 |
| 303 | And_330 | 15062118 | COLOMBIA | NARIÑO | IPIALES | 0.82 | -77.64 | 2868.92 | Andigena | Andigena_3 |
| 304 | And_331 | 15061458 | COLOMBIA | SANTANDER | EL CERRITO | 3.67 | -76.17 | 2270.56 | Andigena | Andigena_3 |
| 305 | And_332 | 15061459 | COLOMBIA | NARIÑO | IPIALES | 0.82 | -77.64 | 2868.92 | Andigena | Andigena_3 |
| 306 | And_333 | 15061461 | COLOMBIA | NARIÑO | IPIALES | 0.82 | -77.64 | 2868.92 | Andigena | Andigena_3 |
| 307 | And_334 | 15062368 | COLOMBIA | NARÑO | IPIALES | 0.83 | -77.64 | 2901.73 | Andigena | Andigena_3 |
| 308 | And_335 | 15062119 | COLOMBIA | NARIÑO | PASTO | 1.21 | -77.28 | 2541.46 | Andigena | Andigena_3 |
| 309 | And_336 | 15061462 | COLOMBIA | NARIÑO | PASTO | 1.21 | -77.28 | 2541.46 | Andigena | Andigena_2 |
| 310 | And_337 | 15061954 | COLOMBIA | NARIÑO | PASTO | 1.21 | -77.28 | 2541.46 | Andigena | Andigena_3 |
| 311 | And_338 | 15061463 | COLOMBIA | NARIÑO | Unknown | 1.29 | -77.36 | 2356.38 | Andigena | Andigena_5 |
| 312 | And_339 | 15061469 | COLOMBIA | NARIÑO | IPIALES | 0.82 | -77.64 | 2868.92 | Andigena | Andigena_5 |
| 313 | And_340 | 15061470 | COLOMBIA | NARIÑO | IPIALES | 0.82 | -77.64 | 2868.92 | Andigena | Andigena_1 |
| 314 | And_341 | 15061471 | COLOMBIA | NARIÑO | PASTO | 1.21 | -77.28 | 2541.46 | Andigena | Andigena_1 |
| 315 | And_342 | 15061475 | COLOMBIA | NARIÑO | IPIALES | 0.82 | -77.64 | 2868.92 | Andigena | Andigena_5 |
| 316 | And_343 | 15061476 | COLOMBIA | NARIÑO | IPIALES | 0.82 | -77.64 | 2868.92 | Andigena | Andigena_5 |
| 317 | And_344 | 15061477 | COLOMBIA | NARIÑO | GUALMATAN | 0.92 | -77.57 | 2917.03 | Andigena | Andigena_5 |
| 318 | And_345 | 15061478 | COLOMBIA | NARIÑO | GUALMATAN | 0.92 | -77.57 | 2917.03 | Andigena | Andigena_5 |
| 319 | And_346 | 15061479 | COLOMBIA | NARIÑO | GUALMATAN | 0.92 | -77.57 | 2917.03 | Andigena | Andigena_5 |
| 320 | And_347 | 15061480 | COLOMBIA | NARIÑO | CONTADERO | 0.91 | -77.55 | 2608.95 | Andigena | Andigena_3 |
| 321 | And_348 | 15061481 | COLOMBIA | NARIÑO | CONTADERO | 0.91 | -77.55 | 2608.95 | Andigena | Andigena_5 |
| 322 | And_349 | 15061482 | COLOMBIA | NARIÑO | CONTADERO | 0.91 | -77.55 | 2608.95 | Andigena | Andigena_4 |
| 323 | And_350 | 15061484 | COLOMBIA | NARIÑO | CONTADERO | 0.91 | -77.55 | 2608.95 | Andigena | Andigena_3 |
| 324 | And_351 | 15061485 | COLOMBIA | NARIÑO | CONTADERO | 0.91 | -77.55 | 2608.95 | Andigena | Andigena_4 |
| 325 | And_352 | 15061486 | COLOMBIA | NARIÑO | IPIALES | 0.82 | -77.64 | 2868.92 | Andigena | Andigena_5 |
| 326 | And_353 | 15061487 | COLOMBIA | NARIÑO | IPIALES | 0.82 | -77.64 | 2868.92 | Andigena | Andigena_2 |
| 327 | And_354 | 15061488 | COLOMBIA | NARIÑO | IPIALES | 0.82 | -77.64 | 2868.92 | Andigena | Andigena_3 |
| 328 | And_355 | 15061489 | COLOMBIA | NARIÑO | IPIALES | 0.82 | -77.64 | 2868.92 | Andigena | Andigena_3 |
| 329 | And_356 | 15061490 | COLOMBIA | NARIÑO | GUALMATAN | 0.92 | -77.57 | 2917.03 | Andigena | Andigena_3 |
| 330 | And_357 | 15062122 | COLOMBIA | NARIÑO | GUALMATAN | 0.92 | -77.57 | 2917.03 | Andigena | Andigena_5 |
| 331 | And_358 | 15061491 | COLOMBIA | NARIÑO | TUQUERRES | 1.09 | -77.62 | 3068.29 | Andigena | Andigena_3 |
| 332 | And_359 | 15061492 | COLOMBIA | NARIÑO | TUQUERRES | 1.09 | -77.62 | 3068.29 | Andigena | Andigena_3 |
| 333 | And_360 | 15061493 | COLOMBIA | NARIÑO | TUQUERRES | 1.09 | -77.62 | 3068.29 | Andigena | Andigena_2 |
| 334 | And_361 | 15061494 | COLOMBIA | NARIÑO | TUQUERRES | 1.09 | -77.62 | 3068.29 | Andigena | Andigena_2 |
| 335 | And_362 | 15061495 | COLOMBIA | NARIÑO | TUQUERRES | 1.09 | -77.62 | 3068.29 | Andigena | Andigena_2 |
| 336 | And_363 | 15061496 | COLOMBIA | NARIÑO | TUQUERRES | 1.09 | -77.62 | 3068.29 | Andigena | Andigena_2 |
| 337 | And_364 | 15061498 | COLOMBIA | NARIÑO | TUQUERRES | 1.09 | -77.62 | 3068.29 | Andigena | Andigena_3 |
| 338 | And_365 | 15062123 | COLOMBIA | NARIÑO | CUMBAL | 0.90 | -77.78 | 3090.99 | Andigena | Andigena_5 |
| 339 | And_366 | 15061499 | COLOMBIA | NARIÑO | CUMBAL | 0.90 | -77.78 | 3090.99 | Andigena | Andigena_5 |
| 340 | And_367 | 15061500 | COLOMBIA | NARIÑO | CUMBAL | 0.90 | -77.78 | 3090.99 | Andigena | Andigena_4 |
| 341 | And_368 | 15061501 | COLOMBIA | NARIÑO | CUMBAL | 0.90 | -77.78 | 3090.99 | Andigena | Andigena_5 |
| 342 | And_369 | 15061503 | COLOMBIA | NARIÑO | CUMBAL | 0.90 | -77.78 | 3090.99 | Andigena | Andigena_5 |
| 343 | And_370 | 15061504 | COLOMBIA | NARIÑO | CUMBAL | 0.90 | -77.78 | 3090.99 | Andigena | Andigena_4 |
| 344 | And_371 | 15061505 | COLOMBIA | NARIÑO | CUMBAL | 0.90 | -77.78 | 3090.99 | Andigena | Andigena_3 |
| 345 | And_372 | 15061507 | COLOMBIA | NARIÑO | CUMBAL | 0.90 | -77.78 | 3090.99 | Andigena | Andigena_5 |
| 346 | And_373 | 15061508 | COLOMBIA | NARIÑO | CUMBAL | 0.90 | -77.78 | 3090.99 | Andigena | Andigena_5 |
| 347 | And_374 | 15061509 | COLOMBIA | NARIÑO | CUMBAL | 0.90 | -77.78 | 3090.99 | Andigena | Andigena_5 |
| 348 | And_375 | 15061511 | COLOMBIA | NARIÑO | IPIALES | 0.82 | -77.64 | 2868.92 | Andigena | Andigena_5 |
| 349 | And_376 | 15061514 | COLOMBIA | NARIÑO | EL MANZANO | 1.30 | -77.16 | 2752.96 | Andigena | Andigena_5 |
| 350 | And_377 | 15062124 | COLOMBIA | NARIÑO | PASTO | 1.21 | -77.28 | 2541.46 | Andigena | Andigena_5 |
| 351 | And_378 | 15061515 | COLOMBIA | NARIÑO | PASTO | 1.21 | -77.28 | 2541.46 | Andigena | Andigena_5 |
| 352 | And_379 | 15061516 | COLOMBIA | NARIÑO | PASTO | 1.21 | -77.28 | 2541.46 | Andigena | Andigena_5 |
| 353 | And_380 | 15061517 | COLOMBIA | NARIÑO | PASTO | 1.21 | -77.28 | 2541.46 | Andigena | Andigena_5 |
| 354 | And_381 | 15061518 | COLOMBIA | NARIÑO | PASTO | 1.21 | -77.28 | 2541.46 | Andigena | Andigena_5 |
| 355 | And_383 | 15061520 | COLOMBIA | NARIÑO | PASTO | 1.21 | -77.28 | 2541.46 | Andigena | Andigena_5 |
| 356 | And_384 | 15061521 | COLOMBIA | NARIÑO | PASTO | 1.21 | -77.28 | 2541.46 | Andigena | Andigena_5 |
| 357 | And_385 | 15061522 | COLOMBIA | NARIÑO | PASTO | 1.21 | -77.28 | 2541.46 | Andigena | Andigena_5 |
| 358 | And_386 | 15061523 | COLOMBIA | NARIÑO | PASTO | 1.21 | -77.28 | 2541.46 | Andigena | Andigena_5 |
| 359 | And_387 | 15061524 | COLOMBIA | NARIÑO | PASTO | 1.21 | -77.28 | 2541.46 | Andigena | Andigena_2 |
| 360 | And_388 | 15061525 | COLOMBIA | NARIÑO | PASTO | 1.21 | -77.28 | 2541.46 | Andigena | Andigena_5 |
| 361 | And_389 | 15061526 | COLOMBIA | NARIÑO | PASTO | 1.21 | -77.28 | 2541.46 | Andigena | Andigena_5 |
| 362 | And_391 | 15061528 | COLOMBIA | NARIÑO | PASTO | 1.21 | -77.28 | 2541.46 | Andigena | Andigena_2 |
| 363 | And_392 | 15061529 | COLOMBIA | NARIÑO | PASTO | 1.21 | -77.28 | 2541.46 | Andigena | Andigena_5 |
| 364 | And_393 | 15061530 | COLOMBIA | NARIÑO | PASTO | 1.21 | -77.28 | 2541.46 | Andigena | Andigena_5 |
| 365 | And_394 | 15061533 | COLOMBIA | NARIÑO | PASTO | 1.21 | -77.28 | 2541.46 | Andigena | Andigena_5 |
| 366 | And_395 | 15061534 | COLOMBIA | NARIÑO | PASTO | 1.21 | -77.28 | 2541.46 | Andigena | Andigena_2 |
| 367 | And_396 | 15061535 | COLOMBIA | NARIÑO | PASTO | 1.21 | -77.28 | 2541.46 | Andigena | Andigena_2 |
| 368 | And_397 | 15061536 | COLOMBIA | NARIÑO | PASTO | 1.21 | -77.28 | 2541.46 | Andigena | Andigena_5 |
| 369 | And_398 | 15061537 | COLOMBIA | NARIÑO | PASTO | 1.21 | -77.28 | 2541.46 | Andigena | Andigena_5 |
| 370 | And_399 | 15062126 | COLOMBIA | NARIÑO | PASTO | 1.21 | -77.28 | 2541.46 | Andigena | Andigena_5 |
| 371 | And_400 | 15061538 | COLOMBIA | NARIÑO | PASTO | 1.21 | -77.28 | 2541.46 | Andigena | Andigena_5 |
| 372 | And_401 | 15061539 | COLOMBIA | NARIÑO | PASTO | 1.21 | -77.28 | 2541.46 | Andigena | Andigena_5 |
| 373 | And_402 | 15061540 | COLOMBIA | CAUCA | MERCADERES | 1.80 | -77.17 | 1135.25 | Andigena | Andigena_5 |
| 374 | And_403 | 15061542 | COLOMBIA | CAUCA | MERCADERES | 1.80 | -77.17 | 1135.25 | Andigena | Andigena_5 |
| 375 | And_404 | 15061543 | COLOMBIA | CAUCA | MERCADERES | 1.80 | -77.17 | 1135.25 | Andigena | Andigena_4 |
| 376 | And_405 | 15061544 | COLOMBIA | CAUCA | POPAYAN | 2.45 | -76.61 | 1727.84 | Andigena | Andigena_5 |
| 377 | And_406 | 15061546 | COLOMBIA | CAUCA | POPAYAN | 2.45 | -76.61 | 1727.84 | Andigena | Andigena_5 |
| 378 | And_407 | 15061548 | COLOMBIA | CAUCA | POPAYAN | 2.45 | -76.61 | 1727.84 | Andigena | Andigena_5 |
| 379 | And_408 | 15061549 | COLOMBIA | CAUCA | POPAYAN | 2.45 | -76.61 | 1727.84 | Andigena | Andigena_5 |
| 380 | And_409 | 15061550 | COLOMBIA | CAUCA | POPAYAN | 2.45 | -76.61 | 1727.84 | Andigena | Andigena_5 |
| 381 | And_410 | 15061551 | COLOMBIA | CAUCA | POPAYAN | 2.45 | -76.61 | 1727.84 | Andigena | Andigena_4 |
| 382 | And_412 | 15061553 | COLOMBIA | CAUCA | POPAYAN | 2.45 | -76.61 | 1727.84 | Andigena | Andigena_5 |
| 383 | And_414 | 15061555 | COLOMBIA | CAUCA | POPAYAN | 2.45 | -76.61 | 1727.84 | Andigena | Andigena_5 |
| 384 | And_415 | 15061556 | COLOMBIA | CAUCA | POPAYAN | 2.45 | -76.61 | 1727.84 | Andigena | Andigena_4 |
| 385 | And_417 | 15061957 | COLOMBIA | CAUCA | POPAYAN | 2.45 | -76.61 | 1727.84 | Andigena | Andigena_5 |
| 386 | And_418 | 15061557 | COLOMBIA | CAUCA | POPAYAN | 2.45 | -76.61 | 1727.84 | Andigena | Andigena_5 |
| 387 | And_419 | 15061560 | COLOMBIA | BOYACA | SOGAMOSO | 5.72 | -72.92 | 2584.94 | Andigena | Andigena_5 |
| 388 | And_420 | 15061561 | COLOMBIA | BOYACA | Unknown | 5.45 | -73.35 | 2379.10 | Andigena | Andigena_3 |
| 389 | And_422 | 15061564 | COLOMBIA | BOYACA | Unknown | 5.45 | -73.35 | 2379.10 | Andigena | Andigena_5 |
| 390 | And_423 | 15061565 | COLOMBIA | BOYACA | Unknown | 5.45 | -73.35 | 2379.10 | Andigena | Andigena_4 |
| 391 | And_424 | 15061566 | COLOMBIA | SANTANDER | Unknown | 6.64 | -73.65 | 705.23 | Andigena | Andigena_5 |
| 392 | And_425 | 15061567 | COLOMBIA | SANTANDER | Unknown | 6.64 | -73.65 | 705.23 | Andigena | Andigena_3 |
| 393 | And_426 | 15061568 | COLOMBIA | SANTANDER | Unknown | 6.64 | -73.65 | 705.23 | Andigena | Andigena_3 |
| 394 | And_427 | 15061571 | COLOMBIA | CAUCA | TOTORO | 2.50 | -76.39 | 2691.07 | Andigena | Andigena_5 |
| 395 | And_428 | 15061958 | COLOMBIA | CAUCA | TOTORO | 2.50 | -76.39 | 2691.07 | Andigena | Andigena_3 |
| 396 | And_429 | 15061575 | COLOMBIA | CAUCA | TOTORO | 2.50 | -76.39 | 2691.07 | Andigena | Andigena_3 |
| 397 | And_430 | 15062130 | COLOMBIA | CAUCA | TOTORO | 2.50 | -76.39 | 2691.07 | Andigena | Andigena_5 |
| 398 | And_431 | 15061576 | COLOMBIA | CAUCA | TOTORO | 2.50 | -76.39 | 2691.07 | Andigena | Andigena_1 |
| 399 | And_432 | 15061577 | COLOMBIA | CAUCA | TOTORO | 2.50 | -76.39 | 2691.07 | Andigena | Andigena_3 |
| 400 | And_433 | 15061578 | COLOMBIA | CAUCA | TOTORA | 2.50 | -76.39 | 2510.00 | Andigena | Andigena_3 |
| 401 | And_434 | 15061579 | COLOMBIA | CAUCA | COCONUCO | 2.34 | -76.49 | 2432.13 | Andigena | Andigena_3 |
| 402 | And_436 | 15062356 | COLOMBIA | CAUCA | COCONUCO | 2.34 | -76.49 | 2432.13 | Andigena | Andigena_2 |
| 403 | And_437 | 15061580 | COLOMBIA | CAUCA | COCONUCO | 2.34 | -76.49 | 2432.13 | Andigena | Andigena_5 |
| 404 | And_439 | 15061583 | COLOMBIA | CAUCA | PURACE | 2.25 | -76.42 | 3523.81 | Andigena | Andigena_4 |
| 405 | And_440 | 15061584 | COLOMBIA | CAUCA | PURACE | 2.25 | -76.42 | 3523.81 | Andigena | Andigena_4 |
| 406 | And_441 | 15061585 | COLOMBIA | CAUCA | PURACE | 2.25 | -76.42 | 3523.81 | Andigena | Andigena_1 |
| 407 | And_442 | 15061586 | COLOMBIA | CAUCA | PURACE | 2.25 | -76.42 | 3523.81 | Andigena | Andigena_1 |
| 408 | And_444 | 15061588 | COLOMBIA | CAUCA | PURACE | 2.25 | -76.42 | 3523.81 | Andigena | Andigena_4 |
| 409 | And_445 | 15062133 | COLOMBIA | CAUCA | Unknown | 2.57 | -76.78 | 1550.95 | Andigena | Andigena_2 |
| 410 | And_446 | 15061600 | COLOMBIA | CAUCA | LA VEGA | 2.00 | -76.78 | 2375.99 | Andigena | Andigena_5 |
| 411 | And_447 | 15061601 | COLOMBIA | CAUCA | LA VEGA | 2.00 | -76.78 | 2375.99 | Andigena | Andigena_5 |
| 412 | And_448 | 15061602 | COLOMBIA | CAUCA | LA VEGA | 2.00 | -76.78 | 2375.99 | Andigena | Andigena_5 |
| 413 | And_449 | 15061603 | COLOMBIA | CAUCA | LA VEGA | 2.00 | -76.78 | 2375.99 | Andigena | Andigena_5 |
| 414 | And_450 | 15061604 | COLOMBIA | CAUCA | LA VEGA | 2.00 | -76.78 | 2375.99 | Andigena | Andigena_4 |
| 415 | And_451 | 15061605 | COLOMBIA | CAUCA | LA VEGA | 2.00 | -76.78 | 2375.99 | Andigena | Andigena_4 |
| 416 | And_452 | 15061607 | COLOMBIA | CAUCA | LA VEGA | 2.00 | -76.78 | 2375.99 | Andigena | Andigena_5 |
| 417 | And_453 | 15061608 | COLOMBIA | CAUCA | SAN SEBASTIAN | 1.84 | -76.77 | 2143.23 | Andigena | Andigena_1 |
| 418 | And_454 | 15061609 | COLOMBIA | CAUCA | SAN SEBASTIAN | 1.84 | -76.77 | 2143.23 | Andigena | Andigena_5 |
| 419 | And_455 | 15061610 | COLOMBIA | CAUCA | SAN SEBASTIAN | 1.84 | -76.77 | 2143.23 | Andigena | Andigena_5 |
| 420 | And_456 | 15061612 | COLOMBIA | CAUCA | SAN SEBASTIAN | 1.84 | -76.77 | 2143.23 | Andigena | Andigena_5 |
| 421 | And_457 | 15061613 | COLOMBIA | CAUCA | SAN SEBASTIAN | 1.84 | -76.77 | 2143.23 | Andigena | Andigena_4 |
| 422 | And_458 | 15061960 | COLOMBIA | CAUCA | SAN SEBASTIAN | 1.84 | -76.77 | 2143.23 | Andigena | Andigena_3 |
| 423 | And_459 | 15061614 | COLOMBIA | CAUCA | SAN SEBASTIAN | 1.84 | -76.77 | 2143.23 | Andigena | Andigena_4 |
| 424 | And_460 | 15061961 | COLOMBIA | CAUCA | SAN SEBASTIAN | 1.84 | -76.77 | 2143.23 | Andigena | Andigena_5 |
| 425 | And_461 | 15062136 | COLOMBIA | CAUCA | SILVIA | 2.61 | -76.34 | 3091.81 | Andigena | Andigena_5 |
| 426 | And_462 | 15062137 | COLOMBIA | CAUCA | SILVIA | 2.61 | -76.34 | 3091.81 | Andigena | Andigena_3 |
| 427 | And_463 | 15061619 | COLOMBIA | CAUCA | SILVIA | 2.61 | -76.34 | 3091.81 | Andigena | Andigena_3 |
| 428 | And_464 | 15061621 | COLOMBIA | CAUCA | SILVIA | 2.61 | -76.34 | 3091.81 | Andigena | Andigena_5 |
| 429 | And_465 | 15061622 | COLOMBIA | CAUCA | SILVIA | 2.61 | -76.34 | 3091.81 | Andigena | Andigena_5 |
| 430 | And_466 | 15061623 | COLOMBIA | CAUCA | SILVIA | 2.61 | -76.34 | 3091.81 | Andigena | Andigena_5 |
| 431 | And_467 | 15061624 | COLOMBIA | CAUCA | SILVIA | 2.61 | -76.34 | 3091.81 | Andigena | Andigena_5 |
| 432 | And_468 | 15062138 | COLOMBIA | VALLE DEL CAUCA | EL CERRITO | 3.67 | -76.17 | 2270.56 | Andigena | Andigena_5 |
| 433 | And_469 | 15061626 | COLOMBIA | VALLE DEL CAUCA | EL CERRITO | 3.67 | -76.17 | 2270.56 | Andigena | Andigena_5 |
| 434 | And_470 | 15061627 | COLOMBIA | VALLE DEL CAUCA | EL CERRITO | 3.67 | -76.17 | 2270.56 | Andigena | Andigena_5 |
| 435 | And_471 | 15061628 | COLOMBIA | VALLE DEL CAUCA | PALMIRA | 3.58 | -76.25 | 1060.55 | Andigena | Andigena_5 |
| 436 | And_472 | 15061629 | COLOMBIA | CUNDINAMARCA | TAUSA | 5.20 | -73.88 | 2936.09 | Andigena | Andigena_5 |
| 437 | And_473 | 15061630 | COLOMBIA | CUNDINAMARCA | TAUSA | 5.20 | -73.88 | 2936.09 | Andigena | Andigena_2 |
| 438 | And_474 | 15061631 | COLOMBIA | CUNDINAMARCA | TAUSA | 5.20 | -73.88 | 2936.09 | Andigena | Andigena_2 |
| 439 | And_475 | 15061632 | COLOMBIA | CUNDINAMARCA | TAUSA | 5.20 | -73.88 | 2936.09 | Andigena | Andigena_4 |
| 440 | And_476 | 15062139 | COLOMBIA | CUNDINAMARCA | TAUSA | 5.20 | -73.88 | 2936.09 | Andigena | Andigena_2 |
| 441 | And_477 | 15061633 | COLOMBIA | CUNDINAMARCA | TAUSA | 5.20 | -73.89 | 3380.00 | Andigena | Andigena_4 |
| 442 | And_478 | 15061634 | COLOMBIA | CUNDINAMARCA | TAUSA | 5.20 | -73.88 | 2936.09 | Andigena | Andigena_5 |
| 443 | And_479 | 15061962 | COLOMBIA | CUNDINAMARCA | TAUSA | 5.20 | -73.88 | 2936.09 | Andigena | Andigena_4 |
| 444 | And_480 | 15061637 | COLOMBIA | CUNDINAMARCA | TAUSA | 5.20 | -73.88 | 2936.09 | Andigena | Andigena_5 |
| 445 | And_481 | 15061638 | COLOMBIA | CUNDINAMARCA | TAUSA | 5.20 | -73.88 | 2936.09 | Andigena | Andigena_5 |
| 446 | And_482 | 15061639 | COLOMBIA | CUNDINAMARCA | TAUSA | 5.20 | -73.88 | 2936.09 | Andigena | Andigena_4 |
| 447 | And_483 | 15061640 | COLOMBIA | CUNDINAMARCA | TAUSA | 5.20 | -73.88 | 2936.09 | Andigena | Andigena_5 |
| 448 | And_484 | 15061641 | COLOMBIA | CUNDINAMARCA | TAUSA | 5.20 | -73.88 | 2936.09 | Andigena | Andigena_4 |
| 449 | And_485 | 15061642 | COLOMBIA | CUNDINAMARCA | TAUSA | 5.20 | -73.88 | 2936.09 | Andigena | Andigena_5 |
| 450 | And_486 | 15061645 | COLOMBIA | BOYACA | RAMIRIQUI | 5.40 | -73.33 | 2312.34 | Andigena | Andigena_5 |
| 451 | And_487 | 15061963 | COLOMBIA | BOYACA | RAMIRIQUI | 5.40 | -73.33 | 2330.68 | Andigena | Andigena_3 |
| 452 | And_488 | 15061646 | COLOMBIA | CUNDINAMARCA | UBATE | 5.31 | -73.82 | 2563.00 | Andigena | Andigena_5 |
| 453 | And_489 | 15061647 | COLOMBIA | CUNDINAMARCA | UBATE | 5.31 | -73.82 | 2563.00 | Andigena | Andigena_5 |
| 454 | And_490 | 15061648 | COLOMBIA | CUNDINAMARCA | UBATE | 5.31 | -73.82 | 2563.00 | Andigena | Andigena_3 |
| 455 | And_491 | 15061649 | COLOMBIA | CUNDINAMARCA | UBATE | 5.31 | -73.82 | 2563.00 | Andigena | Andigena_4 |
| 456 | And_492 | 15061650 | COLOMBIA | BOYACA | CHIQUINQUIRA | 5.62 | -73.82 | 2562.44 | Andigena | Andigena_4 |
| 457 | And_493 | 15061651 | COLOMBIA | BOYACA | CHIQUINQUIRA | 5.62 | -73.82 | 2562.44 | Andigena | Andigena_5 |
| 458 | And_494 | 15061652 | COLOMBIA | BOYACA | CHIQUINQUIRA | 5.62 | -73.82 | 2562.44 | Andigena | Andigena_4 |
| 459 | And_495 | 15061653 | COLOMBIA | CUNDINAMARCA | CHOCONTA | 5.14 | -73.69 | 2652.31 | Andigena | Andigena_3 |
| 460 | And_496 | 15061654 | COLOMBIA | CUNDINAMARCA | CHOCONTA | 5.14 | -73.69 | 2652.31 | Andigena | Andigena_4 |
| 461 | And_497 | 15061655 | COLOMBIA | CUNDINAMARCA | CHOCONTA | 5.14 | -73.69 | 2652.31 | Andigena | Andigena_4 |
| 462 | And_498 | 15061656 | COLOMBIA | CUNDINAMARCA | CHOCONTA | 5.14 | -73.69 | 2652.31 | Andigena | Andigena_4 |
| 463 | And_499 | 15061658 | COLOMBIA | CUNDINAMARCA | CHOCONTA | 5.14 | -73.69 | 2652.31 | Andigena | Andigena_4 |
| 464 | And_500 | 15061659 | COLOMBIA | BOYACA | VENTAQUEMADA | 5.41 | -73.50 | 3043.18 | Andigena | Andigena_1 |
| 465 | And_501 | 15061661 | COLOMBIA | BOYACA | VENTAQUEMADA | 5.41 | -73.50 | 3043.18 | Andigena | Andigena_4 |
| 466 | And_502 | 15061662 | COLOMBIA | BOYACA | VENTAQUEMADA | 5.41 | -73.50 | 3043.18 | Andigena | Andigena_2 |
| 467 | And_503 | 15062357 | COLOMBIA | BOYACA | VENTAQUEMADA | 5.41 | -73.50 | 3043.18 | Andigena | Andigena_5 |
| 468 | And_504 | 15062142 | COLOMBIA | BOYACA | VENTAQUEMADA | 5.41 | -73.50 | 3043.18 | Andigena | Andigena_4 |
| 469 | And_505 | 15061665 | COLOMBIA | BOYACA | SORACA | 5.50 | -73.32 | 2928.56 | Andigena | Andigena_4 |
| 470 | And_506 | 15061666 | COLOMBIA | VALLE DEL CAUCA | TULUA | 4.08 | -76.20 | 979.22 | Andigena | Andigena_5 |
| 471 | And_507 | 15061668 | COLOMBIA | VALLE DEL CAUCA | TULUA | 4.08 | -76.20 | 979.22 | Andigena | Andigena_4 |
| 472 | And_508 | 15062143 | COLOMBIA | VALLE DEL CAUCA | TULUA | 4.08 | -76.20 | 979.22 | Andigena | Andigena_3 |
| 473 | And_509 | 15061667 | COLOMBIA | VALLE DEL CAUCA | TULUA | 4.08 | -76.20 | 979.22 | Andigena | Andigena_3 |
| 474 | And_510 | 15061669 | COLOMBIA | VALLE DEL CAUCA | TULUA | 4.08 | -76.20 | 979.22 | Andigena | Andigena_3 |
| 475 | And_511 | 15061670 | COLOMBIA | VALLE DEL CAUCA | TULUA | 4.08 | -76.20 | 979.22 | Andigena | Andigena_4 |
| 476 | And_512 | 15061671 | COLOMBIA | VALLE DEL CAUCA | TULUA | 4.08 | -76.20 | 979.22 | Andigena | Andigena_3 |
| 477 | And_513 | 15061673 | COLOMBIA | VALLE DEL CAUCA | TULUA | 4.09 | -76.20 | 978.71 | Andigena | Andigena_4 |
| 478 | And_514 | 15061676 | COLOMBIA | CUNDINAMARCA | USME | 4.47 | -74.12 | 2806.95 | Andigena | Andigena_5 |
| 479 | And_515 | 15061677 | COLOMBIA | CUNDINAMARCA | USME | 4.47 | -74.12 | 2806.95 | Andigena | Andigena_3 |
| 480 | And_516 | 15061678 | COLOMBIA | CUNDINAMARCA | USME | 4.47 | -74.12 | 2806.95 | Andigena | Andigena_3 |
| 481 | And_517 | 15061679 | COLOMBIA | CUNDINAMARCA | USME | 4.47 | -74.12 | 2806.95 | Andigena | Andigena_3 |
| 482 | And_518 | 15061680 | COLOMBIA | CUNDINAMARCA | USME | 4.47 | -74.12 | 2806.95 | Andigena | Andigena_4 |
| 483 | And_519 | 15061682 | COLOMBIA | CUNDINAMARCA | USME | 4.47 | -74.12 | 2806.95 | Andigena | Andigena_5 |
| 484 | And_520 | 15061683 | COLOMBIA | CUNDINAMARCA | USME | 4.47 | -74.12 | 2806.95 | Andigena | Andigena_5 |
| 485 | And_521 | 15061684 | COLOMBIA | CUNDINAMARCA | UBATE | 5.31 | -73.82 | 2563.00 | Andigena | Andigena_4 |
| 486 | And_522 | 15061685 | COLOMBIA | CUNDINAMARCA | UBATE | 5.31 | -73.82 | 2563.00 | Andigena | Andigena_3 |
| 487 | And_523 | 15061686 | COLOMBIA | CUNDINAMARCA | CAQUEZA | 4.41 | -73.95 | 1701.93 | Andigena | Andigena_5 |
| 488 | And_524 | 15061687 | COLOMBIA | CUNDINAMARCA | CAQUEZA | 4.41 | -73.95 | 1701.93 | Andigena | Andigena_5 |
| 489 | And_525 | 15061702 | Unknown | Unknown | Unknown | Unknown | Unknown | Unknown | Andigena | Andigena_5 |
| 490 | And_526 | 15061688 | COLOMBIA | CUNDINAMARCA | CAQUEZA | 4.41 | -73.95 | 1701.93 | Andigena | Andigena_5 |
| 491 | And_527 | 15062144 | COLOMBIA | CUNDINAMARCA | CAQUEZA | 4.41 | -73.95 | 1701.93 | Andigena | Andigena_5 |
| 492 | And_528 | 15061689 | COLOMBIA | CUNDINAMARCA | CAQUEZA | 4.41 | -73.95 | 1701.93 | Andigena | Andigena_3 |
| 493 | And_529 | 15061690 | COLOMBIA | CUNDINAMARCA | FOSCA | 4.33 | -73.95 | 2494.18 | Andigena | Andigena_3 |
| 494 | And_530 | 15062145 | COLOMBIA | CUNDINAMARCA | GUTIERREZ | 4.25 | -74.00 | 2154.24 | Andigena | Andigena_3 |
| 495 | And_531 | 15061699 | Unknown | Unknown | Unknown | Unknown | Unknown | Unknown | Andigena | Andigena_3 |
| 496 | And_532 | 15061700 | Unknown | Unknown | Unknown | Unknown | Unknown | Unknown | Andigena | Andigena_4 |
| 497 | And_533 | 15061701 | Unknown | Unknown | Unknown | Unknown | Unknown | Unknown | Andigena | Andigena_3 |
| 498 | And_534 | 15061703 | Unknown | Unknown | Unknown | Unknown | Unknown | Unknown | Andigena | Andigena_3 |
| 499 | And_535 | 15061704 | COLOMBIA | QUINDIO | SALENTO | 4.63 | -75.57 | 1934.53 | Andigena | Andigena_5 |
| 500 | And_537 | 15061706 | COLOMBIA | CALDAS | MANIZALES | 5.07 | -75.52 | 2138.07 | Andigena | Andigena_4 |
| 501 | And_538 | 15061707 | COLOMBIA | CALDAS | MANIZALES | 5.07 | -75.52 | 2138.07 | Andigena | Andigena_3 |
| 502 | And_539 | 15061708 | COLOMBIA | TOLIMA | MURILLO | 4.87 | -75.17 | 3380.00 | Andigena | Andigena_4 |
| 503 | And_540 | 15062146 | COLOMBIA | CALDAS | MANIZALES | 5.07 | -75.52 | 2138.07 | Andigena | Andigena_2 |
| 504 | And_541 | 15061709 | COLOMBIA | CALDAS | MANIZALES | 5.07 | -75.52 | 2138.07 | Andigena | Andigena_3 |
| 505 | And_542 | 15061710 | COLOMBIA | TOLIMA | MURILLO | 4.87 | -75.18 | 3009.85 | Andigena | Andigena_2 |
| 506 | And_543 | 15061712 | COLOMBIA | TOLIMA | MURILLO | 4.87 | -75.18 | 3009.85 | Andigena | Andigena_2 |
| 507 | And_544 | 15062369 | COLOMBIA | BOYACA | CIENEGA | 5.41 | -73.30 | 2750.00 | Andigena | Andigena_2 |
| 508 | And_545 | 15061713 | COLOMBIA | TOLIMA | MURILLO | 4.87 | -75.18 | 3009.85 | Andigena | Andigena_5 |
| 509 | And_546 | 15061714 | COLOMBIA | CUNDINAMARCA | ANOLAIMA | 4.77 | -74.47 | 1592.57 | Andigena | Andigena_2 |
| 510 | And_547 | 15062147 | COLOMBIA | NARIÑO | TUQUERRES | 1.09 | -77.62 | 3068.29 | Andigena | Andigena_2 |
| 511 | And_548 | 15061717 | COLOMBIA | NARIÑO | TUQUERRES | 1.09 | -77.62 | 3068.29 | Andigena | Andigena_5 |
| 512 | And_549 | 15061718 | COLOMBIA | NARIÑO | TUQUERRES | 1.09 | -77.62 | 3068.29 | Andigena | Andigena_2 |
| 513 | And_550 | 15061964 | COLOMBIA | NARIÑO | TUQUERRES | 1.09 | -77.62 | 3068.29 | Andigena | Andigena_5 |
| 514 | And_551 | 15061719 | COLOMBIA | NARIÑO | TUQUERRES | 1.09 | -77.62 | 3068.29 | Andigena | Andigena_5 |
| 515 | And_552 | 15061720 | COLOMBIA | NARIÑO | TUQUERRES | 1.09 | -77.62 | 3068.29 | Andigena | Andigena_2 |
| 516 | And_553 | 15062358 | COLOMBIA | NARIÑO | TUQUERRES | 1.09 | -77.62 | 3068.29 | Andigena | Andigena_3 |
| 517 | And_554 | 15061721 | COLOMBIA | NARIÑO | TUQUERRES | 1.09 | -77.62 | 3068.29 | Andigena | Andigena_5 |
| 518 | And_555 | 15061722 | COLOMBIA | NARIÑO | EL ESPINO | 0.91 | -77.68 | 3186.11 | Andigena | Andigena_5 |
| 519 | And_556 | 15061723 | COLOMBIA | NARIÑO | POTOSI | 0.81 | -77.57 | 2747.63 | Andigena | Andigena_5 |
| 520 | And_557 | 15061724 | COLOMBIA | NARIÑO | POTOSI | 0.81 | -77.57 | 2747.63 | Andigena | Andigena_5 |
| 521 | And_558 | 15062149 | COLOMBIA | NARIÑO | POTOSI | 0.81 | -77.57 | 2747.63 | Andigena | Andigena_2 |
| 522 | And_559 | 15061725 | COLOMBIA | NARIÑO | POTOSI | 0.81 | -77.57 | 2747.63 | Andigena | Andigena_2 |
| 523 | And_560 | 15061726 | COLOMBIA | NARIÑO | LA VICTORIA | 1.45 | -77.08 | 2009.26 | Andigena | Andigena_4 |
| 524 | And_561 | 15061729 | COLOMBIA | NARIÑO | IPIALES | 0.82 | -77.64 | 2868.92 | Andigena | Andigena_5 |
| 525 | And_562 | 15061730 | COLOMBIA | NARIÑO | Unknown | 1.29 | -77.36 | 2356.38 | Andigena | Andigena_5 |
| 526 | And_563 | 15061731 | COLOMBIA | NARIÑO | Unknown | 1.29 | -77.36 | 2356.38 | Andigena | Andigena_3 |
| 527 | And_564 | 15061733 | COLOMBIA | NARIÑO | EL ENGAÑO | 1.17 | -77.17 | 2910.67 | Andigena | Andigena_3 |
| 528 | And_565 | 15061734 | COLOMBIA | NARIÑO | EL ENGAÑO | 1.17 | -77.17 | 2910.67 | Andigena | Andigena_3 |
| 529 | And_566 | 15061735 | COLOMBIA | NARIÑO | EL ENGAÑO | 1.17 | -77.17 | 2910.67 | Andigena | Andigena_2 |
| 530 | And_567 | 15062150 | COLOMBIA | NARIÑO | TUQUERRES | 1.09 | -77.62 | 3068.29 | Andigena | Andigena_5 |
| 531 | And_568 | 15062151 | COLOMBIA | NARIÑO | TUQUERRES | 1.09 | -77.62 | 3068.29 | Andigena | Andigena_5 |
| 532 | And_569 | 15061736 | COLOMBIA | NARIÑO | TUQUERRES | 1.09 | -77.62 | 3068.29 | Andigena | Andigena_5 |
| 533 | And_570 | 15061737 | COLOMBIA | NARIÑO | TUQUERRES | 1.09 | -77.62 | 3068.29 | Andigena | Andigena_5 |
| 534 | And_571 | 15062152 | COLOMBIA | NARIÑO | TUQUERRES | 1.09 | -77.62 | 3068.29 | Andigena | Andigena_3 |
| 535 | And_572 | 15061738 | COLOMBIA | NARIÑO | TUQUERRES | 1.09 | -77.62 | 3068.29 | Andigena | Andigena_3 |
| 536 | And_574 | 15061740 | COLOMBIA | NARIÑO | TUQUERRES | 1.09 | -77.62 | 3068.29 | Andigena | Andigena_2 |
| 537 | And_575 | 15062153 | COLOMBIA | NARIÑO | TUQUERRES | 1.09 | -77.62 | 3068.29 | Andigena | Andigena_3 |
| 538 | And_576 | 15061742 | COLOMBIA | NARIÑO | TUQUERRES | 1.09 | -77.62 | 3068.29 | Andigena | Andigena_5 |
| 539 | And_577 | 15061744 | COLOMBIA | NARIÑO | TUQUERRES | 1.09 | -77.62 | 3068.29 | Andigena | Andigena_4 |
| 540 | And_578 | 15061965 | COLOMBIA | NARIÑO | TUQUERRES | 1.09 | -77.62 | 3068.29 | Andigena | Andigena_5 |
| 541 | And_579 | 15061747 | COLOMBIA | NARIÑO | TUQUERRES | 1.09 | -77.62 | 3068.29 | Andigena | Andigena_5 |
| 542 | And_580 | 15061751 | COLOMBIA | NARIÑO | CUMBAL | 0.90 | -77.78 | 3090.99 | Andigena | Andigena_1 |
| 543 | And_581 | 15061752 | COLOMBIA | NARIÑO | CUMBAL | 0.90 | -77.78 | 3090.99 | Andigena | Andigena_2 |
| 544 | And_582 | 15061753 | COLOMBIA | NARIÑO | CUMBAL | 0.90 | -77.78 | 3090.99 | Andigena | Andigena_3 |
| 545 | And_583 | 15061754 | COLOMBIA | NARIÑO | CUMBAL | 0.90 | -77.78 | 3090.99 | Andigena | Andigena_4 |
| 546 | And_584 | 15061755 | COLOMBIA | NARIÑO | CUMBAL | 0.90 | -77.78 | 3090.99 | Andigena | Andigena_3 |
| 547 | And_585 | 15061756 | COLOMBIA | NARIÑO | GUACHUCAL | 0.97 | -77.73 | 3037.09 | Andigena | Andigena_3 |
| 548 | And_586 | 15061757 | COLOMBIA | NARIÑO | GUACHUCAL | 0.97 | -77.73 | 3037.09 | Andigena | Andigena_3 |
| 549 | And_587 | 15061758 | COLOMBIA | NARIÑO | GUACHUCAL | 0.97 | -77.73 | 3037.09 | Andigena | Andigena_5 |
| 550 | And_588 | 15061759 | COLOMBIA | NARIÑO | GUACHUCAL | 0.97 | -77.73 | 3037.09 | Andigena | Andigena_2 |
| 551 | And_589 | 15061760 | COLOMBIA | NARIÑO | GUACHUCAL | 0.97 | -77.73 | 3037.09 | Andigena | Andigena_5 |
| 552 | And_590 | 15061761 | COLOMBIA | NARIÑO | GUACHUCAL | 0.97 | -77.73 | 3037.09 | Andigena | Andigena_5 |
| 553 | And_591 | 15061762 | COLOMBIA | NARIÑO | GUACHUCAL | 0.97 | -77.73 | 3037.09 | Andigena | Andigena_2 |
| 554 | And_592 | 15061763 | COLOMBIA | NARIÑO | GUACHUCAL | 0.97 | -77.73 | 3037.09 | Andigena | Andigena_3 |
| 555 | And_593 | 15061764 | COLOMBIA | NARIÑO | GUACHUCAL | 0.97 | -77.73 | 3037.09 | Andigena | Andigena_5 |
| 556 | And_594 | 15061765 | COLOMBIA | NARIÑO | GUACHUCAL | 0.97 | -77.73 | 3037.09 | Andigena | Andigena_5 |
| 557 | And_595 | 15061766 | COLOMBIA | NARIÑO | GUACHUCAL | 0.97 | -77.73 | 3037.09 | Andigena | Andigena_5 |
| 558 | And_596 | 15061767 | COLOMBIA | NARIÑO | GUACHUCAL | 0.97 | -77.73 | 3037.09 | Andigena | Andigena_3 |
| 559 | And_597 | 15061768 | COLOMBIA | NARIÑO | GUACHUCAL | 0.97 | -77.73 | 3037.09 | Andigena | Andigena_5 |
| 560 | And_598 | 15061769 | COLOMBIA | NARIÑO | GUACHUCAL | 0.97 | -77.73 | 3037.09 | Andigena | Andigena_3 |
| 561 | And_599 | 15061771 | COLOMBIA | NARIÑO | ALDANA | 0.88 | -77.70 | 3014.11 | Andigena | Andigena_3 |
| 562 | And_600 | 15061772 | COLOMBIA | NARIÑO | IPIALES | 0.82 | -77.64 | 2868.92 | Andigena | Andigena_3 |
| 563 | And_601 | 15061773 | COLOMBIA | NARIÑO | IPIALES | 0.82 | -77.64 | 2868.92 | Andigena | Andigena_2 |
| 564 | And_602 | 15061966 | COLOMBIA | NARIÑO | CAUPUERAN | 0.94 | -77.69 | 3202.46 | Andigena | Andigena_2 |
| 565 | And_603 | 15061776 | COLOMBIA | NARIÑO | CUMBAL | 0.90 | -77.78 | 3090.99 | Andigena | Andigena_4 |
| 566 | And_604 | 15061777 | COLOMBIA | NARIÑO | CUMBAL | 0.90 | -77.78 | 3090.99 | Andigena | Andigena_3 |
| 567 | And_605 | 15061778 | COLOMBIA | NARIÑO | CUMBAL | 0.90 | -77.78 | 3090.99 | Andigena | Andigena_3 |
| 568 | And_606 | 15061784 | COLOMBIA | CAUCA | VEGA | 2.00 | -76.78 | 2375.99 | Andigena | Andigena_3 |
| 569 | And_607 | 15061785 | COLOMBIA | CAUCA | VEGA | 2.00 | -76.78 | 2375.99 | Andigena | Andigena_5 |
| 570 | And_608 | 15062359 | COLOMBIA | CAUCA | VEGA | 2.00 | -76.78 | 2375.99 | Andigena | Andigena_4 |
| 571 | And_609 | 15062370 | COLOMBIA | CAUCA | LA VEGA | 2.00 | -76.78 | 3240.00 | Andigena | Andigena_3 |
| 572 | And_610 | 15061788 | COLOMBIA | VALLE DEL CAUCA | PALMIRA | 3.58 | -76.25 | 1060.55 | Andigena | Andigena_2 |
| 573 | And_611 | 15061967 | COLOMBIA | VALLE DEL CAUCA | PALMIRA | 3.58 | -76.25 | 1060.55 | Andigena | Andigena_5 |
| 574 | And_612 | 15061789 | COLOMBIA | CAUCA | Unknown | 2.57 | -76.78 | 1550.95 | Andigena | Andigena_4 |
| 575 | And_613 | 15061790 | COLOMBIA | CAUCA | Unknown | 2.57 | -76.78 | 1550.95 | Andigena | Andigena_3 |
| 576 | And_614 | 15061792 | HOLANDA | WAGENINGEN | WAGENINGEN | 51.97 | 5.67 | 12.31 | Andigena | Andigena_4 |
| 577 | And_615 | 15061968 | COLOMBIA | ANTIOQUIA | LA UNION | 5.97 | -75.36 | 2476.24 | Andigena | Andigena_3 |
| 578 | And_616 | 15061795 | COLOMBIA | ANTIOQUIA | LA UNION | 5.97 | -75.36 | 2476.24 | Andigena | Andigena_2 |
| 579 | And_617 | 15061797 | COLOMBIA | ANTIOQUIA | LA CEJA | 6.03 | -75.43 | 2145.15 | Andigena | Andigena_3 |
| 580 | And_618 | 15061798 | COLOMBIA | ANTIOQUIA | LA CEJA | 6.03 | -75.43 | 2145.15 | Andigena | Andigena_5 |
| 581 | And_619 | 15061799 | COLOMBIA | ANTIOQUIA | LA CEJA | 6.03 | -75.43 | 2145.15 | Andigena | Andigena_3 |
| 582 | And_620 | 15061800 | COLOMBIA | ANTIOQUIA | LA CEJA | 6.03 | -75.43 | 2145.15 | Andigena | Andigena_5 |
| 583 | And_621 | 15062360 | COLOMBIA | ANTIOQUIA | RIONEGRO | 6.14 | -75.41 | 2095.16 | Andigena | Andigena_4 |
| 584 | And_622 | 15062361 | COLOMBIA | ANTIOQUIA | RIONEGRO | 6.14 | -75.41 | 2095.16 | Andigena | Andigena_5 |
| 585 | And_623 | 15061801 | COLOMBIA | ANTIOQUIA | RIONEGRO | 6.14 | -75.41 | 2095.16 | Andigena | Andigena_4 |
| 586 | And_624 | 15062371 | COLOMBIA | ANTIOQUIA | GUARNE | 6.28 | -75.44 | 2141.64 | Andigena | Andigena_1 |
| 587 | And_625 | 15061969 | COLOMBIA | BOYACA | RAMIRIQUI | 5.40 | -73.33 | 2330.68 | Andigena | Andigena_4 |
| 588 | And_626 | 15061808 | ECUADOR | Unknown | CHUINA | -0.18 | -78.49 | 2785.48 | Andigena | Andigena_4 |
| 589 | And_627 | 15061810 | ESTADOS UNIDOS | WISCONSIN | Unknown | 43.78 | -88.79 | 320.93 | Andigena | Andigena_2 |
| 590 | And_628 | 15061811 | COLOMBIA | CAUCA | SAN SEBASTIAN | 1.84 | -76.77 | 2143.23 | Andigena | Andigena_5 |
| 591 | And_629 | 15062362 | COLOMBIA | CAUCA | Unknown | 2.57 | -76.78 | 1550.95 | Andigena | Andigena_5 |
| 592 | And_630 | 15061813 | COLOMBIA | ANTIOQUIA | SAN PEDRO | 6.46 | -75.56 | 2484.79 | Andigena | Andigena_4 |
| 593 | And_631 | 15061814 | COLOMBIA | ANTIOQUIA | SAN PEDRO | 6.46 | -75.56 | 2484.79 | Andigena | Andigena_4 |
| 594 | And_632 | 15061815 | COLOMBIA | BOYACA | EL COCUY | 6.42 | -72.42 | 3479.20 | Andigena | Andigena_4 |
| 595 | And_633 | 15062372 | COLOMBIA | BOYACA | EL COCUY | 6.41 | -72.44 | 3200.00 | Andigena | Andigena_5 |
| 596 | And_634 | 15061826 | COLOMBIA | CUNDINAMARCA | ZIPAQUIRA | 5.03 | -74.01 | 2666.04 | Andigena | Andigena_5 |
| 597 | And_635 | 15062373 | COLOMBIA | CUNDINAMARCA | ZIPAQUIRÁ | 5.03 | -74.00 | 3350.00 | Andigena | Andigena_4 |
| 598 | And_636 | 15062031 | COLOMBIA | QUINDIO | PIJAO | 4.34 | -75.70 | 1657.08 | Andigena | Andigena_4 |
| 599 | And_637 | 15061893 | COLOMBIA | BOYACA | EL COCUY | 6.42 | -72.42 | 3479.20 | Andigena | Andigena_4 |
| 600 | And_638 | 15061894 | COLOMBIA | BOYACA | EL COCUY | 6.42 | -72.42 | 3479.20 | Andigena | Andigena_5 |
| 601 | And_639 | 15061970 | COLOMBIA | Unknown | Unknown | Unknown | Unknown | Unknown | Andigena | Andigena_4 |
| 602 | And_640 | 15062311 | COLOMBIA | Unknown | Unknown | Unknown | Unknown | Unknown | Andigena | Andigena_3 |
| 603 | And_641 | 15062375 | COLOMBIA | Unknown | Unknown | Unknown | Unknown | Unknown | Andigena | Andigena_3 |
| 604 | And_642 | 15061971 | COLOMBIA | Unknown | Unknown | Unknown | Unknown | Unknown | Andigena | Andigena_5 |
| 605 | And_643 | 15062157 | COLOMBIA | Unknown | Unknown | Unknown | Unknown | Unknown | Andigena | Andigena_2 |
| 606 | And_644 | 15062158 | COLOMBIA | Unknown | Unknown | Unknown | Unknown | Unknown | Andigena | Andigena_3 |
| 607 | And_645 | 15062160 | COLOMBIA | Unknown | Unknown | Unknown | Unknown | Unknown | Andigena | Andigena_3 |
| 608 | And_646 | 15062313 | COLOMBIA | Unknown | Unknown | Unknown | Unknown | Unknown | Andigena | Andigena_3 |
| 609 | And_647 | 15062161 | COLOMBIA | Unknown | Unknown | Unknown | Unknown | Unknown | Andigena | Andigena_3 |
| 610 | And_648 | 15062162 | COLOMBIA | Unknown | Unknown | Unknown | Unknown | Unknown | Andigena | Andigena_4 |
| 611 | And_649 | 15061972 | COLOMBIA | Unknown | Unknown | Unknown | Unknown | Unknown | Andigena | Andigena_5 |
| 612 | And_650 | 15062316 | COLOMBIA | Unknown | Unknown | Unknown | Unknown | Unknown | Andigena | Andigena_3 |
| 613 | And_651 | 15062164 | COLOMBIA | Unknown | Unknown | Unknown | Unknown | Unknown | Andigena | Andigena_3 |
| 614 | And_652 | 15062318 | COLOMBIA | Unknown | Unknown | Unknown | Unknown | Unknown | Andigena | Andigena_3 |
| 615 | And_653 | 15062319 | COLOMBIA | Unknown | Unknown | Unknown | Unknown | Unknown | Andigena | Andigena_3 |
| 616 | And_654 | 15062166 | COLOMBIA | Unknown | Unknown | Unknown | Unknown | Unknown | Andigena | Andigena_2 |
| 617 | And_655 | 15062167 | PERÚ | SANTA RITA | VALLE DE URUBAMBA | -12.10 | -76.94 | 254.07 | Andigena | Andigena_3 |
| 618 | And_656 | 15062168 | PERÚ | Unknown | Unknown | -9.99 | -75.96 | 3654.76 | Andigena | Andigena_3 |
| 619 | And_657 | 15062376 | COLOMBIA | Unknown | Unknown | Unknown | Unknown | Unknown | Andigena | Andigena_1 |
| 620 | And_658 | 15061975 | COLOMBIA | Unknown | Unknown | Unknown | Unknown | Unknown | Andigena | Andigena_4 |
| 621 | And_659 | 15062377 | COLOMBIA | Unknown | Unknown | Unknown | Unknown | Unknown | Andigena | Andigena_5 |
| 622 | And_660 | 15062324 | COLOMBIA | Unknown | Unknown | Unknown | Unknown | Unknown | Andigena | Andigena_4 |
| 623 | And_661 | 15064977 | COLOMBIA | Unknown | Unknown | Unknown | Unknown | Unknown | Andigena | Andigena_3 |
| 624 | And_662 | 686() | COLOMBIA | Unknown | Unknown | Unknown | Unknown | Unknown | Andigena | Andigena_4 |
| 625 | And_663 | 15062327 | COLOMBIA | Unknown | Unknown | Unknown | Unknown | Unknown | Andigena | Andigena_5 |
| 626 | And_664 | 15062328 | COLOMBIA | Unknown | Unknown | Unknown | Unknown | Unknown | Andigena | Andigena_4 |
| 627 | And_665 | 15062378 | COLOMBIA | Unknown | Unknown | Unknown | Unknown | Unknown | Andigena | Andigena_4 |
| 628 | And_666 | 15062379 | Unknown | Unknown | Unknown | Unknown | Unknown | Unknown | Andigena | Andigena_5 |
| 629 | And_667 | 15062380 | Unknown | Unknown | Unknown | Unknown | Unknown | Unknown | Andigena | Andigena_5 |
| 630 | And_668 | 15062381 | Unknown | Unknown | Unknown | Unknown | Unknown | Unknown | Andigena | Andigena_2 |
| 631 | And_669 | 15062382 | Unknown | Unknown | Unknown | Unknown | Unknown | Unknown | Andigena | Andigena_2 |
| 632 | And_670 | 15062383 | Unknown | Unknown | Unknown | Unknown | Unknown | Unknown | Andigena | Andigena_2 |
| 633 | And_671 | 15062384 | Unknown | Unknown | Unknown | Unknown | Unknown | Unknown | Andigena | Andigena_5 |
| 634 | And_672 | 15062385 | Unknown | Unknown | Unknown | Unknown | Unknown | Unknown | Andigena | Andigena_5 |
| 635 | And_673 | 15062386 | Unknown | Unknown | Unknown | Unknown | Unknown | Unknown | Andigena | Andigena_5 |
| 636 | And_674 | 15062387 | Unknown | Unknown | Unknown | Unknown | Unknown | Unknown | Andigena | Andigena_5 |
| 637 | And_675 | 15062388 | Unknown | Unknown | Unknown | Unknown | Unknown | Unknown | Andigena | Andigena_4 |
| 638 | And_676 | 15062389 | Unknown | Unknown | Unknown | Unknown | Unknown | Unknown | Andigena | Andigena_4 |
| 639 | And_677 | 15062390 | Unknown | Unknown | Unknown | Unknown | Unknown | Unknown | Andigena | Andigena_4 |
| 640 | And_678 | 15062391 | Unknown | Unknown | Unknown | Unknown | Unknown | Unknown | Andigena | Andigena_5 |
| 641 | And_680 | 15062393 | Unknown | Unknown | Unknown | Unknown | Unknown | Unknown | Andigena | Andigena_4 |
| 642 | And_681 | 15062394 | Unknown | Unknown | Unknown | Unknown | Unknown | Unknown | Andigena | Andigena_1 |
| 643 | And_682 | 15062395 | Unknown | Unknown | Unknown | Unknown | Unknown | Unknown | Andigena | Andigena_4 |
| 644 | And_683 | 15062396 | Unknown | Unknown | Unknown | Unknown | Unknown | Unknown | Andigena | Andigena_4 |
| 645 | And_685 | 15062398 | Unknown | Unknown | Unknown | Unknown | Unknown | Unknown | Andigena | Andigena_4 |
| 646 | And_686 | 15062399 | Unknown | Unknown | Unknown | Unknown | Unknown | Unknown | Andigena | Andigena_4 |
| 647 | And_687 | 15062400 | Unknown | Unknown | Unknown | Unknown | Unknown | Unknown | Andigena | Andigena_4 |
| 648 | And_688 | 15062401 | Unknown | Unknown | Unknown | Unknown | Unknown | Unknown | Andigena | Andigena_4 |
| 649 | And_689 | 15062402 | Unknown | Unknown | Unknown | Unknown | Unknown | Unknown | Andigena | Andigena_4 |
| 650 | And_690 | 15062403 | Unknown | Unknown | Unknown | Unknown | Unknown | Unknown | Andigena | Andigena_4 |
| 651 | And_691 | 15062404 | Unknown | Unknown | Unknown | Unknown | Unknown | Unknown | Andigena | Andigena_3 |
| 652 | And_692 | 15062405 | Unknown | Unknown | Unknown | Unknown | Unknown | Unknown | Andigena | Andigena_4 |
| 653 | And_694 | 15061711 | COLOMBIA | TOLIMA | MURILLO | 4.87 | -75.17 | 3380.00 | Andigena | Andigena_4 |
| 654 | And_696 | 15061022 | PERU | LIBERTAD | SANTIAGO DE CHUCO | -8.22 | -78.20 | 3500.00 | Andigena | Andigena_5 |
| 655 | And_697 | 15060812 | Unknown | Unknown | Unknown | Unknown | Unknown | Unknown | Andigena | Andigena_4 |
| 656 | And_698 | 15060045 | COLOMBIA | NARIÑO | PASTO | 1.21 | -77.28 | 2541.46 | Andigena | Andigena_5 |
| 657 | And_700 | 15060142 | PERU | CONCEPCION | ESTACION ESPERIMENTAL LA MOLINA | -11.92 | -75.31 | 3286.00 | Andigena | Andigena_4 |
| 658 | And_701 | 15062312 | Unknown | Unknown | Unknown | Unknown | Unknown | Unknown | Andigena | Andigena_3 |
| 659 | And_702 | 15062314 | Unknown | Unknown | Unknown | Unknown | Unknown | Unknown | Andigena | Andigena_3 |
| 660 | And_703 | 15062315 | Unknown | Unknown | Unknown | Unknown | Unknown | Unknown | Andigena | Andigena_3 |
| 661 | And_704 | 15062321 | PERÚ | SANTA RITA | VALLE DE URUBAMBA | -12.10 | -76.94 | 254.07 | Andigena | Andigena_3 |
| 662 | And_706 | ALGODONA | Unknown | Unknown | Unknown | Unknown | Unknown | Unknown | Andigena | Andigena_2 |
| 663 | And_707 | UNICA | Unknown | Unknown | Unknown | Unknown | Unknown | Unknown | Andigena | Andigena_4 |
| 664 | And_708 | TOCANA ROJA | Unknown | Unknown | Unknown | Unknown | Unknown | Unknown | Andigena | Andigena_5 |
| 665 | And_709 | PARDA BASICA | Unknown | Unknown | Unknown | Unknown | Unknown | Unknown | Andigena | Andigena_5 |
| 666 | And_710 | PARDA BILINGUE | Unknown | Unknown | Unknown | Unknown | Unknown | Unknown | Andigena | Andigena_4 |
| 667 | And_711 | UVA | Unknown | Unknown | Unknown | Unknown | Unknown | Unknown | Andigena | Andigena_4 |
| 668 | And_712 | UVILLA PLOMA | Unknown | Unknown | Unknown | Unknown | Unknown | Unknown | Andigena | Andigena_5 |
| 669 | And_713 | CURIPAMBA 1 | Unknown | Unknown | Unknown | Unknown | Unknown | Unknown | Andigena | Andigena_5 |
| 670 | And_714 | NARAMPUERA | Unknown | Unknown | Unknown | Unknown | Unknown | Unknown | Andigena | Andigena_4 |
| 671 | And_715 | RUBI ROJA | Unknown | Unknown | Unknown | Unknown | Unknown | Unknown | Andigena | Andigena_5 |
| 672 | And_716 | CURIQUINGA | Unknown | Unknown | Unknown | Unknown | Unknown | Unknown | Andigena | Andigena_2 |
| 673 | And_717 | COLLABABLANCA | Unknown | Unknown | Unknown | Unknown | Unknown | Unknown | Andigena | Andigena_4 |
| 674 | And_718 | RUBI NEGRA 1 | Unknown | Unknown | Unknown | Unknown | Unknown | Unknown | Andigena | Andigena_5 |
| 675 | And_719 | ROJA ICA HUILA | Unknown | Unknown | Unknown | Unknown | Unknown | Unknown | Andigena | Andigena_5 |
| 676 | Cha_1 | 15060205 | COLOMBIA | NARIÑO | CUMBAL | 0.90 | -77.78 | 3090.99 | Phureja | Phureja_1 |
| 677 | Cha_2 | 15062516 | COLOMBIA | NARIÑO | CUMBAL | 0.91 | -77.79 | 3131.09 | Phureja | Phureja_1 |
| 678 | Cha_6 | 15062520 | COLOMBIA | NARIÑO | CUMBAL | 0.91 | -77.79 | 3131.09 | Phureja | Phureja_1 |
| 679 | Cha_7 | 15062521 | COLOMBIA | NARIÑO | CUMBAL | 0.91 | -77.79 | 3131.09 | Phureja | Phureja_1 |
| 680 | Cha_9 | 15062522 | COLOMBIA | NARIÑO | PASTO | 1.22 | -77.27 | 3030.00 | Phureja | Phureja_3 |
| 681 | Cha_10 | 15062523 | COLOMBIA | NARIÑO | IPIALES | 0.83 | -77.64 | 2901.73 | Phureja | Phureja_1 |
| 682 | Cha_11 | 15062524 | COLOMBIA | NARIÑO | POTOSI | 0.81 | -77.57 | 2739.01 | Phureja | Phureja_1 |
| 683 | Cha_12 | 15062525 | Unknown | Unknown | Unknown | Unknown | Unknown | Unknown | Phureja | Phureja_3 |
| 684 | Cha_13 | 15062526 | Unknown | Unknown | Unknown | Unknown | Unknown | Unknown | Phureja | Phureja_2 |
| 685 | Cha_15 | 15062528 | COLOMBIA | NARIÑO | CUMBAL | 0.91 | -77.79 | 3131.09 | Phureja | Phureja_2 |
| 686 | Cha_16 | 15062529 | Unknown | Unknown | Unknown | Unknown | Unknown | Unknown | Phureja | Phureja_2 |
| 687 | Cha_17 | 15062530 | COLOMBIA | NARIÑO | Unknown | 1.22 | -77.27 | 3030.00 | Phureja | Phureja_2 |
| 688 | Cha_18 | 15062531 | COLOMBIA | NARIÑO | CUMBAL | 0.91 | -77.79 | 3131.09 | Phureja | Phureja_2 |
| 689 | Cha_19 | 15062532 | COLOMBIA | NARIÑO | Unknown | 1.22 | -77.27 | 3030.00 | Phureja | Phureja_1 |
| 690 | Cha_20 | 15062533 | Unknown | Unknown | Unknown | Unknown | Unknown | Unknown | Phureja | Phureja_1 |
| 691 | Cha_21 | 15062534 | COLOMBIA | NARIÑO | Unknown | 1.22 | -77.27 | 3030.00 | Phureja | Phureja_1 |
| 692 | Cha_24 | 15062536 | COLOMBIA | NARIÑO | Unknown | 1.22 | -77.27 | 3030.00 | Phureja | Phureja_1 |
| 693 | Cha_25 | 15062537 | COLOMBIA | NARIÑO | Unknown | 1.22 | -77.27 | 3030.00 | Phureja | Phureja_3 |
| 694 | Cha_27 | 15062539 | COLOMBIA | NARIÑO | Unknown | 1.22 | -77.27 | 3030.00 | Phureja | Phureja_1 |
| 695 | Cha_28 | 15062540 | COLOMBIA | NARIÑO | Unknown | 1.22 | -77.27 | 3030.00 | Phureja | Phureja_2 |
| 696 | Cha_29 | 15062541 | COLOMBIA | NARIÑO | Unknown | 1.22 | -77.27 | 3030.00 | Phureja | Phureja_1 |
| 697 | Cha_30 | 15062542 | COLOMBIA | NARIÑO | Unknown | 1.22 | -77.27 | 3030.00 | Phureja | Phureja_1 |
| 698 | Cha_32 | 15062544 | COLOMBIA | NARIÑO | Unknown | 1.22 | -77.27 | 3030.00 | Phureja | Phureja_1 |
| 699 | Cha_33 | 15062545 | COLOMBIA | NARIÑO | Unknown | 1.22 | -77.27 | 3030.00 | Phureja | Phureja_1 |
| 700 | Cha_34 | 15062546 | COLOMBIA | NARIÑO | Unknown | 1.22 | -77.27 | 3030.00 | Phureja | Phureja_3 |
| 701 | Cha_35 | 15062547 | COLOMBIA | NARIÑO | Unknown | 1.22 | -77.27 | 3030.00 | Phureja | Phureja_1 |
| 702 | Cha_36 | 15062548 | COLOMBIA | NARIÑO | Unknown | 1.22 | -77.27 | 3030.00 | Phureja | Phureja_1 |
| 703 | Cha_37 | 15062549 | COLOMBIA | NARIÑO | Unknown | 1.22 | -77.27 | 3030.00 | Phureja | Phureja_1 |
| 704 | Cha_39 | 15062551 | COLOMBIA | NARIÑO | Unknown | 1.22 | -77.27 | 3030.00 | Phureja | Phureja_3 |
| 705 | Cha_40 | 15062552 | COLOMBIA | NARIÑO | Unknown | 1.22 | -77.27 | 3030.00 | Phureja | Phureja_3 |
| 706 | Cha_41 | 15062553 | COLOMBIA | NARIÑO | Unknown | 1.22 | -77.27 | 3030.00 | Phureja | Phureja_3 |
| 707 | Cha_42 | 15062554 | COLOMBIA | NARIÑO | Unknown | 1.22 | -77.27 | 3030.00 | Phureja | Phureja_1 |
| 708 | Cha_43 | 15062555 | COLOMBIA | NARIÑO | Unknown | 1.22 | -77.27 | 3030.00 | Phureja | Phureja_1 |
| 709 | Cha_45 | 15062557 | COLOMBIA | NARIÑO | Unknown | 1.22 | -77.27 | 3030.00 | Phureja | Phureja_1 |
| 710 | Cha_46 | 15062558 | COLOMBIA | NARIÑO | Unknown | 1.22 | -77.27 | 3030.00 | Phureja | Phureja_1 |
| 711 | Cha_47 | 15062559 | COLOMBIA | NARIÑO | Unknown | 1.22 | -77.27 | 3030.00 | Phureja | Phureja_1 |
| 712 | Cha_49 | 15062561 | Unknown | Unknown | Unknown | Unknown | Unknown | Unknown | Phureja | Phureja_2 |
| 713 | Cha_50 | 15062562 | Unknown | Unknown | Unknown | Unknown | Unknown | Unknown | Phureja | Phureja_2 |
| 714 | Cha_52 | 15062564 | Unknown | Unknown | Unknown | Unknown | Unknown | Unknown | Phureja | Phureja_1 |
| 715 | Cha_55 | 15062567 | Unknown | Unknown | Unknown | Unknown | Unknown | Unknown | Phureja | Phureja_1 |
| 716 | Cha_56 | 15062568 | Unknown | Unknown | Unknown | Unknown | Unknown | Unknown | Phureja | Phureja_1 |
| 717 | Cha_57 | 15062569 | Unknown | Unknown | Unknown | Unknown | Unknown | Unknown | Phureja | Phureja_2 |
| 718 | Cha_59 | 15062571 | Unknown | Unknown | Unknown | Unknown | Unknown | Unknown | Phureja | Phureja_1 |
| 719 | Cha_60 | 15062572 | Unknown | Unknown | Unknown | Unknown | Unknown | Unknown | Phureja | Phureja_1 |
| 720 | Cha_61 | 15062573 | Unknown | Unknown | Unknown | Unknown | Unknown | Unknown | Phureja | Phureja_1 |
| 721 | Cha_62 | 15062574 | Unknown | Unknown | Unknown | Unknown | Unknown | Unknown | Phureja | Phureja_1 |
| 722 | Cha_63 | 15062575 | Unknown | Unknown | Unknown | Unknown | Unknown | Unknown | Phureja | Phureja_1 |
| 723 | Cha_66 | 15060630 | PERÚ | LA LIBERTAD | AGALLPAMPA | -7.98 | -78.54 | 3124.95 | Phureja | Phureja_1 |
| 724 | Cha_67 | PCH-OB-33 | Unknown | Unknown | Unknown | Unknown | Unknown | Unknown | Phureja | Phureja_1 |
| 725 | Phu_1 | 15060002 | COLOMBIA | VALLE DEL CAUCA | CALI | 3.46 | -76.52 | 982.58 | Phureja | Phureja_1 |
| 726 | Phu_2 | 15060077 | COLOMBIA | NARIÑO | CUMBAL | 0.90 | -77.78 | 3090.99 | Phureja | Phureja_1 |
| 727 | Phu_3 | 15060204 | COLOMBIA | NARIÑO | CUMBAL | 0.90 | -77.78 | 3090.99 | Phureja | Phureja_1 |
| 728 | Phu_4 | 15061362 | COLOMBIA | NORTE DE SANTANDER | PAMPLONA | 7.39 | -72.66 | 2431.17 | Phureja | Phureja_1 |
| 729 | Phu_5 | 15061456 | COLOMBIA | NARIÑO | IPIALES | 0.82 | -77.64 | 2868.92 | Phureja | Phureja_1 |
| 730 | Phu_6 | 15061780 | COLOMBIA | CAUCA | SAN SEBASTIAN | 1.84 | -76.77 | 2143.23 | Phureja | Phureja_1 |
| 731 | Phu_7 | 15061781 | COLOMBIA | CAUCA | SAN SEBASTIAN | 1.84 | -76.77 | 2143.23 | Phureja | Phureja_1 |
| 732 | Phu_9 | 15061980 | COLOMBIA | NARIÑO | Unknown | 1.22 | -77.27 | 3030.00 | Phureja | Phureja_3 |
| 733 | Phu_11 | 15061982 | COLOMBIA | NARIÑO | Unknown | 1.22 | -77.27 | 3030.00 | Phureja | Phureja_1 |
| 734 | Phu_13 | 15061984 | COLOMBIA | NARIÑO | Unknown | 1.22 | -77.27 | 3030.00 | Phureja | Phureja_1 |
| 735 | Phu_14 | 15061985 | COLOMBIA | NARIÑO | Unknown | 1.22 | -77.27 | 3030.00 | Phureja | Phureja_1 |
| 736 | Phu_15 | 15061986 | COLOMBIA | NARIÑO | Unknown | 1.22 | -77.27 | 3030.00 | Phureja | Phureja_1 |
| 737 | Phu_16 | 15061987 | COLOMBIA | NARIÑO | Unknown | 1.22 | -77.27 | 3030.00 | Phureja | Phureja_1 |
| 738 | Phu_17 | 15061988 | COLOMBIA | NARIÑO | Unknown | 1.22 | -77.27 | 3030.00 | Phureja | Phureja_1 |
| 739 | Phu_18 | 15061989 | COLOMBIA | NARIÑO | Unknown | 1.22 | -77.27 | 3030.00 | Phureja | Phureja_1 |
| 740 | Phu_20 | 15061991 | COLOMBIA | NARIÑO | Unknown | 1.22 | -77.27 | 3030.00 | Phureja | Phureja_3 |
| 741 | Phu_21 | 15061992 | COLOMBIA | NARIÑO | Unknown | 1.22 | -77.27 | 3030.00 | Phureja | Phureja_1 |
| 742 | Phu_22 | 15061994 | COLOMBIA | NARIÑO | Unknown | 1.22 | -77.27 | 3030.00 | Phureja | Phureja_3 |
| 743 | Phu_23 | 15061996 | COLOMBIA | NARIÑO | Unknown | 1.22 | -77.27 | 3030.00 | Phureja | Phureja_1 |
| 744 | Phu_24 | 15061997 | COLOMBIA | NARIÑO | Unknown | 1.22 | -77.27 | 3030.00 | Phureja | Phureja_2 |
| 745 | Phu_25 | 15061998 | COLOMBIA | NARIÑO | Unknown | 1.22 | -77.27 | 3030.00 | Phureja | Phureja_1 |
| 746 | Phu_26 | 15061999 | COLOMBIA | NARIÑO | Unknown | 1.22 | -77.27 | 3030.00 | Phureja | Phureja_1 |
| 747 | Phu_27 | 15062000 | COLOMBIA | NARIÑO | Unknown | 1.22 | -77.27 | 3030.00 | Phureja | Phureja_1 |
| 748 | Phu_28 | 15062001 | COLOMBIA | NARIÑO | Unknown | 1.22 | -77.27 | 3030.00 | Phureja | Phureja_1 |
| 749 | Phu_29 | 15062002 | COLOMBIA | NARIÑO | Unknown | 1.22 | -77.27 | 3030.00 | Phureja | Phureja_1 |
| 750 | Phu_30 | 15062005 | COLOMBIA | NARIÑO | Unknown | 1.22 | -77.27 | 3030.00 | Phureja | Phureja_3 |
| 751 | Phu_32 | 15062009 | PERU | Unknown | LIMA | -12.04 | -77.04 | 138.30 | Phureja | Phureja_1 |
| 752 | Phu_33 | 15062010 | PERU | Unknown | LIMA | -12.04 | -77.04 | 138.30 | Phureja | Phureja_1 |
| 753 | Phu_34 | 15062015 | COLOMBIA | NARIÑO | IPIALES | 0.82 | -77.64 | 2868.92 | Phureja | Phureja_1 |
| 754 | Phu_35 | 15062020 | BOLIVIA | Unknown | LA PAZ | -16.50 | -68.15 | 3781.17 | Phureja | Phureja_1 |
| 755 | Phu_36 | 15062021 | ESTADOS UNIDOS | VIRGINIA | Unknown | 37.43 | -78.66 | 213.64 | Phureja | Phureja_1 |
| 756 | Phu_37 | 15062025 | COLOMBIA | NARIÑO | PASTO | 1.21 | -77.28 | 2541.46 | Phureja | Phureja_3 |
| 757 | Phu_38 | 15062026 | COLOMBIA | CAUCA | SILVIA | 2.61 | -76.34 | 3091.81 | Phureja | Phureja_1 |
| 758 | Phu_39 | 15062027 | COLOMBIA | CAUCA | SILVIA | 2.61 | -76.34 | 3091.81 | Phureja | Phureja_2 |
| 759 | Phu_40 | 15062028 | COLOMBIA | BOYACA | VIRACACHA | 5.44 | -73.30 | 2517.05 | Phureja | Phureja_3 |
| 760 | Phu_41 | 15062029 | COLOMBIA | CAUCA | SAN SEBASTIAN | 1.84 | -76.77 | 2143.23 | Phureja | Phureja_3 |
| 761 | Phu_42 | 15062031 | COLOMBIA | QUINDIO | PIJAO | 4.33 | -76.69 | 1723.93 | Phureja | Phureja_2 |
| 762 | Phu_43 | 15062507 | COLOMBIA | NARIÑO | Unknown | 1.22 | -77.27 | 3030.00 | Phureja | Phureja_1 |
| 763 | Phu_45 | 15062509 | COLOMBIA | CUNDINAMARCA | CHOCONTA | 5.14 | -73.68 | 2652.31 | Phureja | Phureja_2 |
| 764 | Phu_47 | 15062511 | COLOMBIA | NARIÑO | ALDANA | 0.88 | -77.70 | 3015.49 | Andigena | Andigena_5 |
| 765 | Phu_48 | 15062512 | COLOMBIA | CAUCA | SAN SEBASTIAN | 1.84 | -76.77 | 2134.43 | Phureja | Phureja_2 |
| 766 | Phu_51 | 15062515 | Unknown | Unknown | Unknown | Unknown | Unknown | Unknown | Phureja | Phureja_3 |
| 767 | Phu_59 | 15062591 | Unknown | Unknown | Unknown | Unknown | Unknown | Unknown | Phureja | Phureja_1 |
| 768 | Phu_60 | 15062592 | Unknown | Unknown | Unknown | Unknown | Unknown | Unknown | Phureja | Phureja_2 |
| 769 | Phu_61 | 15062593 | Unknown | Unknown | Unknown | Unknown | Unknown | Unknown | Phureja | Phureja_2 |
| 770 | Phu_62 | 15062594 | Unknown | Unknown | Unknown | Unknown | Unknown | Unknown | Phureja | Phureja_3 |
| 771 | Phu_63 | 15062595 | Unknown | Unknown | Unknown | Unknown | Unknown | Unknown | Phureja | Phureja_1 |
| 772 | Phu_64 | 15062596 | Unknown | Unknown | Unknown | Unknown | Unknown | Unknown | Phureja | Phureja_3 |
| 773 | Phu_66 | 15062598 | Unknown | Unknown | Unknown | Unknown | Unknown | Unknown | Phureja | Phureja_1 |
| 774 | Phu_67 | 15062599 | Unknown | Unknown | Unknown | Unknown | Unknown | Unknown | Phureja | Phureja_2 |
| 775 | Phu_68 | 15062600 | Unknown | Unknown | Unknown | Unknown | Unknown | Unknown | Phureja | Phureja_2 |
| 776 | Phu_69 | 15062601 | Unknown | Unknown | Unknown | Unknown | Unknown | Unknown | Phureja | Phureja_1 |
| 777 | Phu_70 | 15062582 | Unknown | Unknown | Unknown | Unknown | Unknown | Unknown | Phureja | Phureja_2 |
| 778 | Phu_71 | 15062583 | Unknown | Unknown | Unknown | Unknown | Unknown | Unknown | Phureja | Phureja_3 |
| 779 | Phu_73 | 15061047 | PERÚ | JUNIN | TARMA | -11.42 | -75.69 | 3056.41 | Phureja | Phureja_3 |
| 780 | Phu_74 | ILS 2334 | Unknown | Unknown | Unknown | Unknown | Unknown | Unknown | Phureja | Phureja_1 |
| 781 | Phu_75 | ILS 1045 | COLOMBIA | ANTIOQUIA | EL CARMEN DE VIBORAL | 6.08 | -75.34 | 2153.09 | Phureja | Phureja_1 |
| 782 | Phu_76 | Colecta 2 Guican | Unknown | Unknown | Unknown | Unknown | Unknown | Unknown | Phureja | Phureja_1 |
| 783 | Phu_77 | CIP 703293 | PERÚ | LIMA | Unknown | -12.04 | -77.04 | 138.30 | Phureja | Phureja_1 |
| 784 | Phu_78 | CIP 703308 | PERÚ | LIMA | Unknown | -12.04 | -77.04 | 138.30 | Phureja | Phureja_1 |
| 785 | Phu_79 | Cip 703511 | PERÚ | LIMA | Unknown | -12.04 | -77.04 | 138.30 | Phureja | Phureja_1 |
| 786 | Phu_81 | Colecta 2 Paño de Mano Quinua | Unknown | Unknown | Unknown | Unknown | Unknown | Unknown | Phureja | Phureja_1 |
| 787 | Phu_83 | Colecta 26 Paño de Mano | Unknown | Unknown | Unknown | Unknown | Unknown | Unknown | Phureja | Phureja_1 |
| 788 | Phu_85 | Colecta 3 Guican | Unknown | Unknown | Unknown | Unknown | Unknown | Unknown | Phureja | Phureja_1 |
| 789 | Phu_88 | Colecta 10 Guican | Unknown | Unknown | Unknown | Unknown | Unknown | Unknown | Phureja | Phureja_1 |
| 790 | Phu_91 | Colecta 9 Guican | Unknown | Unknown | Unknown | Unknown | Unknown | Unknown | Phureja | Phureja_1 |
| 791 | Phu_94 | Chaucha Botella | COLOMBIA | NARIÑO | Unknown | 1.22 | -77.27 | 3030.00 | Phureja | Phureja_1 |
| 792 | Phu_97 | 15062312 | Unknown | Unknown | Unknown | Unknown | Unknown | Unknown | Phureja | Phureja_1 |
| 793 | Phu_98 | 15062314 | Unknown | Unknown | Unknown | Unknown | Unknown | Unknown | Phureja | Phureja_3 |
| 794 | Phu_108 | 15062314 | Unknown | Unknown | Unknown | Unknown | Unknown | Unknown | Phureja | Phureja_1 |
| 795 | Phu_109 | COLOMBIA | COLOMBIA | NARIÑO | Unknown | 1.22 | -77.27 | 3030.00 | Phureja | Phureja_3 |
| 796 | Phu_110 | Bola De Sal | COLOMBIA | NARIÑO | Unknown | 1.22 | -77.27 | 3030.00 | Phureja | Phureja_1 |
| 797 | Phu_111 | Leona | COLOMBIA | NARIÑO | Unknown | 1.22 | -77.27 | 3030.00 | Phureja | Phureja_1 |
| 798 | Phu_112 | Chaucha Uvilla | COLOMBIA | NARIÑO | Unknown | 1.22 | -77.27 | 3030.00 | Phureja | Phureja_3 |
| 799 | Phu_113 | Borrega | COLOMBIA | NARIÑO | Unknown | 1.22 | -77.27 | 3030.00 | Phureja | Phureja_1 |
| 800 | Phu_114 | Purita | COLOMBIA | NARIÑO | Unknown | 1.22 | -77.27 | 3030.00 | Phureja | Phureja_1 |
| 801 | Phu_115 | 15061767 | COLOMBIA | NARIÑO | GUACHUCAL | 0.97 | -77.73 | 3037.09 | Phureja | Phureja_1 |
| 802 | Phu_116 | Tornilla Roja | COLOMBIA | NARIÑO | Unknown | 1.22 | -77.27 | 3030.00 | Phureja | Phureja_1 |
| 803 | Phu_117 | Ratona Negra | COLOMBIA | NARIÑO | Unknown | 1.22 | -77.27 | 3030.00 | Phureja | Phureja_1 |
| 804 | Phu_118 | Yema de Huevo | COLOMBIA | NARIÑO | Unknown | 1.22 | -77.27 | 3030.00 | Phureja | Phureja_3 |
| 805 | Phu_119 | Rubi Negra | COLOMBIA | NARIÑO | Unknown | 1.22 | -77.27 | 3030.00 | Andigena | Andigena_5 |
| 806 | Phu_120 | Yema de Huevo | COLOMBIA | NARIÑO | Unknown | 1.22 | -77.27 | 3030.00 | Phureja | Phureja_3 |
| 807 | Phu_121 | Aguacata | COLOMBIA | NARIÑO | Unknown | 1.22 | -77.27 | 3030.00 | Phureja | Phureja_1 |
| 808 | Phu_122 | Morasurco | COLOMBIA | VALLE DEL CAUCA | EL CERRITO | 3.68 | -76.31 | 995.40 | Andigena | Andigena_5 |
| 809 | Phu_123 | Guaneña | COLOMBIA | Unknown | Unknown | Unknown | Unknown | Unknown | Phureja | Phureja_1 |

*In latitude and longitude information were showed only two decimals
