# Supplementary material for: Genetic diversity and association mapping in the Colombian Central Collection of Solanum tuberosum L. Andigenum group using SNPs markers
Source: PLoS One. 2017 Mar 3;12(3):e0173039. doi: 10.1371/journal.pone.0173039 (PMC5336250; doi:10.1371/journal.pone.0173039)
Supplement: S4 Table — (DOC) [file pone.0173039.s005.doc]

**S4 Table. Pairwise genetic differentiation (F_ST_) values between populations of *S. tuberosum*** in the Colombian Central Collection.

| **Population** | **Subpopulations** |  |  |  |  |  |
| --- | --- | --- | --- | --- | --- | --- |
| **Phureja** |  | **Phureja_1** | **Phureja_2** | **Phureja_3** |  |  |
|  | **Phureja_1** | - |  |  |  |  |
|  | **Phureja_2** | 0.161***** | - |  |  |  |
|  | **Phureja_3** | 0.235***** | 0.435***** | - |  |  |
|  |  |  |  |  |  |  |
| **Andigena** |  | **Andigena_1** | **Andigena_2** | **Andigena_3** | **Andigena_4** | **Andigena_5** |
|  | **Andigena_1** | - |  |  |  |  |
|  | **Andigena_2** | 0.195***** | - |  |  |  |
|  | **Andigena_3** | 0.216***** | 0.080***** | - |  |  |
|  | **Andigena_4** | 0.122***** | 0.071***** | 0.077***** | - |  |
|  | **Andigena_5** | 0.181***** | 0.049***** | 0.031***** | 0.057***** | - |

* Significance at *p* = 0.000 at 1023 permutations
