# Supplementary material for: Genetic diversity and association mapping in the Colombian Central Collection of Solanum tuberosum L. Andigenum group using SNPs markers
Source: PLoS One. 2017 Mar 3;12(3):e0173039. doi: 10.1371/journal.pone.0173039 (PMC5336250; doi:10.1371/journal.pone.0173039)
Supplement: S5 Table — (DOC) [file pone.0173039.s006.doc]

***S5 Table. Summary of characteristics for the six groups identified by the morphological analysis in tetraploid accessions of the Colombian Central Collection of*** S. tuberosum.

| **Group** | **Number accessions** | **Variables** | | | | | | | | | | | | | | |
| --- | --- | --- | --- | --- | --- | --- | --- | --- | --- | --- | --- | --- | --- | --- | --- | --- |
|  |  | **ST** | **BC** | **BS** | **PTSC** | **PTSIC** | **STSC** | **DSTSC** | **PTFC** | **STFC** | **DSTFC** | **GTS** | **PFC** | **PFIC** | **SFC** | **DSFC** |
| **Group 1** | **108** | green with few spots | green with many points whites | globose | yellow | Pallid | Purple | dotted spots | Cream | absent | absent | compressed | purple | dark | white | white acumen-back sides leaves |
| **Group 2** | **59** | green with few spots | green with pigmented areas | globose | purple | Dark | Yellow | scattered spots | cream | purple | this in the vascular ring is narrow | compressed | purple | dark | white | white acumen-back sides leaves |
| **Group 3** | **119** | pigmented with few green | green with many points whites | globose | red | Dark | Absent | Absent | cream | absent | absent | compressed | purple | dark | white | white acumen-back sides leaves |
| **Group 4** | **32** | green with few spots | green with pigmented areas | globose | purple | Pallid | Absent | Absent | cream | absent | absent | compressed | white | absent | absent | Absent |
| **Group 5** | **159** | green with few spots | green with many points whites | globose | purple | Dark | Yellow | scattered spots | cream | absent | absent | round | purple | dark | white | white acumen-back sides leaves |
| **Group 6** | **147** | green with few spots | green with many points whites | globose | purple | Pallid | Yellow | Absent | cream | absent | absent | compressed | purple | dark | white | white acumen-back sides leaves |

ST (Stem Color); BC (Berry Color); BS (Berry Shape); PTSC (Primary Tuber Skin Color); PTSIC (Primary Tuber Skin Intensity Color); STSC (Secondary Tuber Skin Color); DSTSC (Distribution of Secondary Tuber Skin Color); PTFC (Primary Tuber Flesh Color); STFC (Secondary Tuber Flesh Color); DSTFC (Distribution of Secondary Tuber Flesh Color); GTS (General Tuber Shape); PFC (Primary Flower Color); PFIC (Primary Flower Intensity Color); SFC (Secondary Flower Color); DSFC (Distribution of Secondary Flower Color)
